# Supplementary material for: Identification of Homeobox Transcription Factors in a Dimorphic Fungus Talaromyces marneffei and Protein-Protein Interaction Prediction of RfeB
Source: J Fungi (Basel). 2024 Sep 30;10(10):687. doi: 10.3390/jof10100687 (PMC11508405; doi:10.3390/jof10100687)
Supplement: Supplementary file 1 [file jof-10-00687-s001.zip › Supplemental Data S1.pdf]

Supplemental Data S1.
Method:

The FTFD pipeline (http://ftfd.snu.ac.kr/index.php?a=view) was used to identify homeodomain-containing transcription factors in selected fungal species. A total of 141 proteins were retrieved and listed below.

>**HOMEO_Pm0001 (EEA19358.1)**
MEDNDPFMFGPDASTDMETLHAIYYANNDDNNNNNGAYNPNNLHDISTTSCYLDALTSTSDDAATDWSNLQLSLNGISSPTSSQSSVTHEGSMFQEAFLS
SHHNPHVSNLTLAIQGMNNNAGIFDAIPASTSGSDELAMNNNLPYMDDKPSRMTKQSLRALKDWKAQHGDATKPTMQQIHRLQRRTNLSSEQILNWFSHT
KRRADTFGYSHSQQLGTLSQDFSANATGAATMERPSTPAVRDIIYNTPNTPLGDMDPLERWENSPPEHEAASFSDIAKALTHSPLVSITTVPNKSQTSTR
SASRADDSASTRSSINGYHRDPSTSSLDSDMPGSITWSDHSGTSAQSGRSESKPEYRRRARKRRTNPLSRGLNINSLRSNNVPKTFQCTFCTDSFKTKYD
WSRHEKSLHLALESWMCSPFGAVSHEDNEDEPKCAYCNTSSPTEEHLHTHRYLACDGRPESERTYYRKDHLRQHLRLVHGCRLIPSMEKWKHTPELVRSR
CGFCDKVLTTWQARIDHLAAHFRAGASMAEWVGGWGFEPHISRLVENGIPPFLIHQERNTVAPFTGSIALQSKKQTAKVSSHDNGNGNDDDNDAVDKGEE
EEKSKPYINQYSYPATGEGSWYDCFETQLATYINTVIATEGRIPSDKEIQDQGRRFVFDDDDPWHQTIAENVMWLEYFKERHGFFVDRPLI
>HOMEO_Pm0002 (EEA20547.1)
MNYLHHPAYPYGVHAGIHLDQPGLVHPAIANNIDGYVLTRPAYELADYYTHMPLMEDYEEYSENLSRPRLTKEQVDTLEAQFQAHPKPNSNKKRELAVQT
NLSLPRVANWFQNRRAKAKQQKRQEEFERMQREAKEKEDQSKSIKDEEQDYGLPGCDQKSPIHKDDNSHGTTKSPTPTQASNYTKDRPQTSDSSSLSRPK
HQKTGSDLAQEKTYASLQRAISAAVAARDQYTGPSDDHISVGPTDRAFDVRQTNIPVPSANNTPQTSANSTLSEWGSSRDSSIAWTPSQSPEEGYEFGSL
NNVPFASEVAQMDNSPNPDVSTSHGFGSITSRSQQMWNPQFQGRAEIHASDPMYGSLSYSSLQPPSATSSRRPSASEELADSISGIGINTAALAGSMDSS
MWRRPEKELDIAARRKRPRPAAIGTAHHRLSTNPSMVSPNARMATFGAPHTIRHAKSSHTLGSRYAGVRKLSATQRSPLGYSSFAEAATAAAANASSESR
QKHRLHTSASVGNLAPPTPLTPEDFQHMLLTPTTSDTQMNFSTPHLTDTQGNFPVTQSMQINVASPPETPLTLDVFSAMQYQNMAPPLSATPQYASFTDY
SPITSEPLTGVSWAVSTPDASLFPSSLQSQRQQPPIYIYEQDDDEHQDPKWTLSGDDGSSLYGSTKASATPPANMMTVSEEHDPNGMTQFHIHEFPKQQE
AHRNVAQQLAPQIPKNYTFSNQTPSDF
>HOMEO_Pm0003 (EEA23074.1)
MSSIDSQPAVAGSNVATPTNSTPSPSMTSPAASAPSSSASRRPPRKSTLTQQQKNQKRQRATQDQLVTLEQEFNKNPTPTAAVRERIAQEINMTERSVQI
WFQNRRAKIKMIAKKSIETGEDCDSIPDSMRQYLAMHFDPHKPGARELFGRPNGLGGLSNGYGMETTPGKIVIQHFTCRTLSIGSWRRIGQNAMDLVIFY
SPDKATMTYYINNDSAGYKIEYPFSYIKSVMLDNGDLTPNANGMPTRPGGLVIELNRPPIFWMDCSNSGGFFQCGDFTEDQQASRVMTHHLGGHPKVLSV
QLAKLVSLETFQNRLDFNYSVSAPITPPQDIHRPASQPNRFTLAQVGIYPDSHLSVNQMPRGHKRQRSRSVPAAIDLSYLHTPIAPFPLQHPQVSHMAPN
PNMYAPIPQNPNALHALGNDLRINTAATFVDPQSYPMSATTMSDFAVASPSFFSAAPPTESIAITGNPGDQFNMPYVSPSPMLDQSKMMNQPPTSMANFS
HADPLIANHSPPLSTLHNTISNDMFNFGADQQQGMTEDGFVLSEMYAKHHINQFPDGPSFDFSMNALSETQSPMPSDMHGFETIQTTSA
>HOMEO_Pm0004 (EEA23091.1)
MAVTYTGGYSAPGSQTLPSFRELLPEHLHNEIDQAAYYSSQSSPTDRHRTPDPTSYNKSRSSLPYPHSSATSGGGRGPSPILPPLRDLQSSSRHHDRPMS
SYDDAPSRSGSRAGRGYRTSMEEDPRYRSTSGGAAMYGNQNAFDPRYAQQQQSYHGSSYPSSNHSNQSNDYEYPASSPMAGHSHTLPASSGNFGVMGAGG
DPLDSRGKRRRGNLPKPVTDVLRAWFHEHLDHPYPTEEDKQIFMSRTGLSISQISNWFINARRRQLPALRNQLRNSDGEHIPRGHSPMSDVETSMSPPHS
HMASPTHSRHR
>**HOMEO_Pm0006 (EEA24489.1)** StlA
MAPPQKPETFMLSSEAQQSLPQDAQVALQQVDNLKYFLISAPVDWSPDQLIRRFLLPTGDYISCVLWNNLFHISGTDIVRCLSFRFQAFGRPVKNSKKFE
EGIFSDLRNLKSGTDASLEEPKSPFLDFLYKNNCIRTQKKQKVFYWYSVPHDRLFLDALERDLKREKMGQEATTVAVAEPALSFEFDSSQSLYEQLTKAQ
QANSSSFSAHASTTYGQPTSPIVRSIDVMPPPQMAPSTMTMLPEDTGSSVLYNQVPMAMKRENDYAQIQYERSTSIPFNRLHQRHASMPSYMEYSPAPSF
VSSQFEDYSNRGISFEPITPPQQPMLGAEPAYIANEETGLYTAIPDVGSAAYHPMMQLPPSNFANPLGATSRHFQANVFSVIEGSPTYKQRRRRSSIPPG
ITNAIAAAQVSGAQPQPTSYAAYRPSDLRRSISNSVGPVTEITDSNDQSAAGSLQSDYTSTVASQKNMLHEVSRTGTPLPSLEEGVEHSHTMMQQSQDEL
ASLGNQDSIDISVPHSLLGRSDRPGPVRRARSATMMELGPYPQKSHSCPIPSCGRLFKRLEHLKRHVRTHTQERPYPCPYCNKAFSRSDNLAQHRRTHET
QQDGHNSQQSLSDDFLENEEKEFGSLEEESPGSDSHSFIHPSVVSMSTMTPMSVASTMSMQASMSTMVAPHLISPQYLQQQM
>HOMEO_Pm0007 (EEA24568.1)
MSIIGVYRSAPSPQSDLYRHPLAPVSDSIALRHSNQGAQESKMSLSSGAPIFAPSAATPVKENAPSSSGNAQGESSSATVLPNPAETGQFPSQFSNSNLF
ERAAVQVEKSEVVTEQNRPADSWGAPGSNMSVSQNTQADEPESHGEENDDDLAEDFSNGEEGEGGQGRSAGDEDNSKKTKRFRLTHNQTRFLMSEFTRQA
HPDAAHRERLSREIPGLSPRQVQVWFQNRRAKLKRLTSQDRDRVLKSRALPDHFDRTQMLQQPYNPRHSANTSPTSPTTRSSFSTGHKPLAVNNIKRNPG
DEYPISPASAYGNYVSSPGISEPFSPTNNTGHPATLPRVPGVHPPHSHEYNRSHSFSSSYANWQYPQRLHMPPSESGIKTEHTMNPLHRPTASYPGLAGT
IPEGAYDRHSSQASSVDHGTTTQTNSPLPSAMAYQAGQHSHTNEQYPSSNESAYNTPVGYRHVLSLQTGQLPPPQEYQVSPFTPSYNFDSFYQQYTHENS
STVSLPASYMRSSQQSTYEPASGTYSYDNQDMSHRLAASQPGGTR
>**HOMEO_Pm0008 (EEA26382.1)**
MEYFNFEEASYASNNVQDDVASDCQEVDEVEAVENYESLFFDKNFQLHPVSGLTADGEVGNLHDQGLDPQLRANYPDVPEPTPGSQYPMFRSPLPCDFCR
QMGFDCFLIQRGILAAGCTCCVSLYRECSFTHAKTPGKFLHTLHPVGEDDVTSTGGLTGRRALKSVGSTKFDHLESRGRKSGARFSREAVRILKNWLSEH
YQHPYPNEAEKDALKERTGLKRSQIANWLANARRRGKVLPSTRDSSPTPGALDIPGSTRTSSASSTTSTSSSSTLEYALMTPLERWKHSPPEHEAASTSA
IIRAMASASLPPPSPSHAQQQQQHQAYYPPAMSGNGRSFSRKTGSSNDSTLSFQAPSVSSYETENKSSISDYSFASAFSHRSSQHSLNSADRKERRRRRK
QMPPVPAFDRQPVRGARIFQCTFCIDSFPAKYDWQRHEKSVHLALEKWTCAPHGGIITISDGKKACAFCRAPNPTDDHLESHNYLTCQEKTVQERTFYRK
DHLNQHLRLMHDVKYDSSMDQWRSTTNDIKSRCGLCDTNFTTWKERVDHIAQHFKNGADMSKWKGDWGFEPYVQRLVENGIPPYLIASERANLNQDRVPG
SGSQKKVIDSFTGSSSVVLLVPTDETSFTRLESELKAYIGGQLLDGSTPTDKEIQDQARMIVYGSNDLWNQTCADNIVWLSVLKRDCGMEDLPGLENTNL
EDLEMQPPFAGRMQHPPLETNTFAAGNVRHSWFSGASLGSAAVSASGLQSPAFISSSGFPSAAASMPGSLAGSFSGSIGVSSAGPSSSVIPGLSSGWSSN
FSVGERSASDWAGSAAFADPMAQSNFDLELLQQLNGSESHNTDIDTSMFGMEGLTSQSEIMRTVAAKTPQSFDPSTMTTAETQPISIPTTTAAADMHSYF
EPSFSSQPGYS
>HOMEO_Pm0009 (EEA26588.1)
MVAADSTPITATSSSPSQPFNCSSPANLHYAFLVHSQKTLTQNLPPRVDNKLLARQKRRRTSPEDHAVLEAEYRKNPKPDKVARASIVSQVSLGEKEVQI
WFQNRRQNDRRKSKPLEPHELVGPRALDGIPNESPSNHGTSSPRDGEQETPEQPEGNFEKDDEDALADPSNNTESNEVEIGMPAGSLKPETSFSSLGSAT
LGNDVEIMRDVTGLGIHVNEKKRRLSDTGGGPREHDGECVQVTIKSPPSLRISMSFDGEALLRKQGEPTPSPPKARTAVRISLSSDGEALIRTQDEPSPS
KNRMRLIPGRVPRQTGLRRSVSAINFGTPRASPSIREGSGMKPFGRSRDARMWETYCDNDARSALSTPIGSQPSTSIRTPGLYRSGSHRSLARHSFSKPN
LSSPAIGSNNPQDVAEPSREKRRKLSRTVSSLGRLESVQQSIHMTQDSSSGLKSTHQTGKGVERWDLEAGDSDKENWLPGTYHRPDPRRHQRAKSHSQRA
VLKENGVRKNTVLDDLALTKQGRLCRGPRGSKDVKASSQTIAKLDAEVAAFMTKPGSSSREEDLDCIQGLLSLSQGAWQ
>**HOMEO_Pm0010 (EEA26628.1)**
MNPDQPEPSQGEGPTPEEMLHQVLHQMTAMRTDMADMRGRINHLEQTPAPSAQNPEPPVVPNVPPQAPNPPVLQPRHATNHPEKYDDEDRSKFMPFILEL
ESKLIVDGPAIGDAYAQLVYAYGRFTGKARTKVYPWMRIHGSAQVMGTVTFVTLTEFFRHIRILFEDQQLVERANSELSRLRQGATPFQEFITEFERLLL
LARGQAWPDDIQISRLKPALNQEIRKACIGKSMPILYEAYCEELHRVANDLEEYQRIENLRRNRRVNRPHYPHTNDQPAAATAPPLYPNAMDISNANPAR
PPLTCYNCQQLGHIARLCNNAYVPRYAAGPQTRRPVAPSNRRTPVTNNVNPITLPAPANIPTSATMPRDEELDLENE
>**HOMEO_Pm0011 (EEA28535.1)**

MIQSKQQKPITLFTMSSQDGTVEVNPARAKKRSTAASTTTSQNGPPQSQSFFFVDPASSTREKRAHVMRHHIQAKRKQNMLASHSDRHSRREPRVYPWMK
KSNSNSDENDSKPLREVITTQKSTNYKREESSNAVAQPTDSLTGLRKDPFATLAFDASLPWAEEHLADLWTSRLTYWSGPNSHMKNRIFQGAMRNRTTFE
AVVLGYCARWEFNLATPREDGYKDSSRVQFYESRVRQALTAKSPHRSSQAFDEEALAMTLTGLALQEERFGDKDKACEYAEQAKELQLYQQRQSISAANA
VNAVCRPFLLYVLGTMDPHSSAVSSDEMAQMVDFLHMAHQSMATDKDENYLKEVPQRSAAFQFDSPLFQLLSSGPRPSQVPMENRNFVVNINKPTNERAR
TAALIYIVLALSEFRQRKSKVVRFLGHLLQLVADYDLDRNPACESFMYFLMEETFDSDLRQPDRAWQSNDLLQIHKRLPFELQFQFNEMLLGYLMLLPPV
TTVDAFEKGLQDMKHTNVR
>**HOMEO_Af0011 (Afu1g10580)**
MNYLHHPYAFGGHNAVQFDQSIAYDPAMAHHAMVHPMDGYLYPHPPYEMVDYYHQPIMDYEEYAENLSRPRLTKEQVETLEAQFQAHPKPSSNVKRQLAA
QTNLSLPRVANWFQNRRAKAKQQKRQEEFERMQKAKAEAEEAARNKADSADDTSDSNQSDSTTKEETDKANDTKTSESGGPGEQTKTSTSSRSKHQKTRS
ESAREATFASLQRALNAAVAAREHYGSECEQDRNDGNSVGGSASPTTFPLSARGNDHDDSESAHSALNTPFSAWESGKDSSASWTSRGAQEPFAYTGLAT
PSFPTMESSLQPVSQGEEWASQLATSAESLPGHRAPMDVDSSSYGAIHYSVKPELSRQGSSEDLASTLQGIGIDTSGSPHGLSQLTGRSSWKDAGKELDL
AARRKRPRPAAIGTSRSSSMLAGSTSMSPTMRLPNGAGHAVRQSKSAQSLGSRYAGVRKASTAQRSPFNLATFAEAGTLNCKPEMSSMLQPVTTGGLAPP
TPLTPENLHHLLPATPSDGGYCLSAQPTSHLFPTTQPMQINIASPPATPLAVDMMSSYSYHGVAPPMSAPAHYTTFPEYSSCDGTPLTGRSWADATSIPP
SDQSFAARCQMEMPSVTYEQAVDPTCATADIGMFSSKDIDMHMSTGAPAEDARPTEFQIQEFPEQQEIHRFVAQQLPSHKPKAYTFAANSTPHNFQN
>**HOMEO_Af0012 (Afu1g12475)**
MNENISQLLTAPSKPIILTERNDWPAWYKEIRLASMCKNIWPYVDPDTKDAPVVPDEPAFPAYGDYQIGALRYSDLDEKNRVEYDHALRMYPWMRDDVRR
IRGDVAYIALVIATSVSKYARGFIVDEDDPREMLRLLKGPFEPSL
>**HOMEO_Af0013 (Afu1g15550)**
MEYFDFDGASFGSHAPDDEVASDRFELDENDAIENYESLLREQPLDFPNDLPEQDALNPQTEAPSVNAQSNNALPIHRAKEPCDFCRHMNLDCFISDRGA
LQNGCTCCVILFRECSFTHAKKPGKYMETLQSISENADIPTVGPKGRKTLKSLTGMTISEDMEGRGRKSSSRLSRDAVRILKTWLLEHLDHPYPSEQEKD
ELKKRTGLKRSQISNWLANARRRGKARPLPPSNSSVPGAINIPGQQQQQQPNIALMTPLERWKYSPPENEPASTTDILRALVNNPLDSTRQPGHVRSLSR
KGSSNDSSHANSNIFKAPSISSLGESRSVSRSSVSDLSFASAFSHRSSLESFGSMERKERRRRRKPSTALNTFNQQKARNSRIFQCTFCTETFATKYDWQ
RHEKSLHLALDKWTCSPQGGVVYVNGANRCVFCMASDPDTDHLESHCYSTCQEKTLAERTFYRKDHLNQHLRLMHNVKFNSYMNQWQSTTTELKSRCGFC
GTTLTTWKDRVEHLAAHFKNGADMTQWQGDWGFEPFVQGLVENAMPPYLIGQDRKTLNPYTTSKFLGQSCCSPTTASPGLIEPNDVNCFHRLQRELTAYI
HKQVAEGIIPTDQMIQDQARIVIYGNTDPWNQTSADNPVWLSILKRDTGLEVVPDSEHIQLDNLNMQPPFAAHGGLRQPPVEGNPLARSLCDKMPLKPES
FSPALRSPTFAGTGRSSAAPSMPGSSAASFTGSFGIAPSGVNSGLSTDWGSNFSAGVSSFSTPATGSVDQFVQMGFEPEFLQQLNDRYGEMHIDEMDGMM
FGVEQEHTRNSALVPEGDVYKLAGSTDAAAPLSNVGSAPINIPSPKQTGAVAQQDQASIGDPFYPSAVGV
>HOMEO_Af0014 (Afu2g00360)
MDHPDYLHSLTSHSAPDPAENGTWFNADDTVDPTLFGINLQHDSTLDTNQLNNMYGLSSESWFDGNGPEASELFPSAATQDPGVLQAAGSTTEEIRSEMQ
TQTPATAIVSQSMLNVTQWLDGAHRPPQPCSYCRKHRLQCLILRTTPANPNPITACSSCVALFRECSLARGEKRQPSRFETLSPVMGHLHGVTELEEDEG
DQAEASAPDVNCVDRQKESKQFVRRGVRILKEWFRDHRDFPYPSEDEKARLVRETGFSRKRMSTWFANARRRQKERFDTPPAAQICRSGSPMPASRLTLM
TPMERWQNSPPEEEAVPESAILNAISSWEVAPRSENEDAMFDSFLNLDETSSHLGSSLSSMGSRRSESSTSVSSAWSHHSGDSSLPFPLHHPRPRVRRRT
RPRGPMNEGQYQCTFCTQSFKKKHDWLRHEKSVHLQLDAWICTPDLNDLQPGNLPSGCRFCDHSASSIDHWNDHEFEVCAQKPIADRSFSRKDYLWQHLR
KFHGCTKLPIENLDQWHSARSDVRSRCGFCDASLPTWAARGDHLADHFKQGCRMHQWVGDWGLDPDMLGALQNAVLPSERTMEATTDLPLSAG
>HOMEO_Af0015 (Afu3g12160)
MSDPEPSAAACSSSPPTVQTPESATLNYAFLVHSQKTLTQNLPPRVDNKLLARQKRRRTSPEDHAVLEAEYQRNPKPDKAARANIVSRVSLGEKEVQIWF
QNRRQNDRRKSKPLQPHELLAPRSDVSSPLRQATSDDSPSGEQVSLSGGEQYDGREKDRDLAELLEDGVPQSSYESVESGGRDNTAQLSLSSQTSLSQAS
EISQELHKAVPDATSTQQDNDTPSDDTQLSAKRKRSISDLRGDVPTTLQQQQQQPTPGGLQVLKSPPSLRLSLSFDGEAMVRREGELTPSPPKGRNSLRI
AMSSDGKAVIRTEDEPSPSKGRISMFSTRSSRFAGLRRSSSAVALGTPRAGAIEKEKAFGRSRDPRNWESFFDTDARSALSTPTSSQSAPNSASPNLMFA
PGQRSLTRSLSARHTNMSTSTTHDYLNTPIPQHAGEKRRKLSRTVSSLGRLESSFSNLNRTPSGAYNISKVRDTMKDKDDLDIECGDSDKENWIPGTRVS
HVRQRAASHHQSHRPVLKEANGRDGRINRNLAATGGRSRISQPSHRKSIKSMPELDADVSAFMAGGGVASQEEDLDCVQGLLSLSQGAWR
>HOMEO_Af0016 (Afu4g04320)
MSISGPCVWLSSLSPPATSHLSAITNHLPATSWNSEQVLSRSRVSPPRLPYPGEKGGHSVQKLAVDMQPSAVLKAENALSASTVVATSNHVRSSEAEMPP
PKEAESRSTEETLSRMRLDQLNHSESHPSGVSTPASPSVSKAASLEAKKRPQEETRGDEKDDGEAMGISSDEGGDQKNSSDAKTDKKKMKRFRLTHNQTR
FLMSEFTRQAHPDAAHRERLSREIPGLTPRQVQVWFQNRRAKLKRLTSNDRERMLKSRALPDDFDTTQVLRTPFDNKPLSETPVASPRNYMASTTDSNSL
KMLLTDGLQRVNDDEYVVSPLSSSSTTGNCFPSTGPDRTPDHFSQHGILGPRPVATLPELQRNTRGTFPFPRSSSFSEVSYPTGLHLPGRFSRPGVEPLN
HPGLPYARRPIDYGIPRPANGMVMGYDHSRAMEGSVSPTGQQEQQLPYNVDNNNQQIPSYQTTLTMPAPKGFGSIEMNSHMQPRHMPALHSIPVSDAPDY
RPYSYEHHPYSMQTALPYSQANASSMSLPASFPPDTTHIPQVAVSTEDRINQSPQILDPMRGKFGNPSYDYTSYM
>**HOMEO_Af0017 (Afu4g10110)**
MSQHGEVDDESGKMAISYASGLSADHTQSLPSFRETRLPGNGVQSAAARGPSPILPSIRDLQAIPDCSLKAPATGLPDHRGPSRTEAYPVQEFRGGAVGA
PSYSPTGLPGSMGDRRDLYAGNSIPAAVHAQPQYPSYPGVIYQSDSEQASSQSLPPSQQSNFGILGDSVDSKNKRRRGNLPKPVTDILRAWFHEHLDHPY
PSEEDKQMFMTRTGLTISQISNWFINARRRQLPALRNQMRNGASDLDSQRQSPFSDMEQTSSEAPNRLNSTTKH
>HOMEO_Af0018 (Afu4g10220)
MSLSRLCPLSTGALSLSHNSLHSFVSLVSHSFFFLSSSSFLLTPPVSCQPSLFFSFPLSLFFTLHSFVSSILSLYTSDPSTMSTTSTAANTASSSSAAST
PSPSNSSVTTAPSTSTSRRPPRKSTLTQQQKNNKRQRATQDQLVTLELEFNKNPTPTAATRERIAQEINMTERSVQIWFQNRRAKIKMLAKKSIETGEGC
DSIPESMRQYLAMQFDPSKAGARDPFGRTGAYGANGAYPSESAPSGKVVIHHFTCRSLTIGSWRRIGQNAMDLVVFYSPEKACMTYYINNDSAGYKIEYP
FSYIKNITLESGDANAQPNGVPTRPAGLLVELNRPPLFYMDSSNSGGFYQCGDFTEDQQASQILVHHLGGHPKVLSVQLAKLVSLESFQNRLAYSNLTMA
PPMSPHFIQRPASQPNQFAPAFMNMYQDQSTLNIPVARGHKRQRSRSVPVAVDFSAMQMSHFPSYNMPQTPTPYHNADSGIFAPVPQSAQPLALNLRIDT
SPSYGFDPRGHPMSATTTGSPSDFASPSIFATSATGESTPVATHMGPSFSLPFVAPSVDSSSMGHAAPSYSNVSHADPMIAEHSPPLSNMAHTPQDMYTL
RTEQQTNLGDDVMGLNEMFVKQSMDYSVPTTMGLECNTYDLPMQTLSGQTSPLPATNYQSMEGVDLNSMAEKLSCSKLQLGVSGPSVQVNKCLYRRHIFS
ILATDKFSLLFHLFIFLFWSFNVVMPGSEQNGRYLGNFHRFWSSPGRRVRNFVTPTSWNLFRERYAAQAACWLPGTADKILPVFKCLLTWRHEAS
>**HOMEO_Af0019 (Afu5g06190)**
MYTQHGAQMAPPQKPETFMLSNEAQQSLPHDAQVALQQVDNLKYFLLSAPVDWPRDQLIRRYLLPTGDYISCVLWNNLFHISGTDIVRCLAFRFQAFGRP
VKNSKKFEEGIFSDLRNLKAGTDATLEEPKSPFLDFLYKNNCIRTQKKQKVFYWYSVPHDRLFLDALERDLKREKMGQEATTLAVSEPALSFEFDSSQSL
YEQLTKAQQANSSSFHAGTTFGQSNSPIVRTVDAMPPPQMAPPAMTVLQDDAGNQAMYSTMPMAHPLTQAIIKREPDYGQIQYDCNGIPIRIHQRHASMP
SFVVEYSPAPSFVSSQYEDYSNRGLSFEPVTPPQHSVPLGPEPAYIANEDTGLYTAIPDITSAATFNPMMQLPPSNLASASYPMPARTYHSNVYSVIEGS
PTYKQRRRRSSIPPSAAANGHAQTTSAPSQQIAYAAHRPSDLRRSVSNSVVPAAEGEDTHEASNHGANGYTSAVLPQRNLLHDMSRNGTPLSNVEENPES
NPQTTGHQDDLVTLPNGEAYEGGVQNNVSNKVERFTPGPIRRARSATMMELGPYPQKSHSCPIPSCGRLFKRLEHLKRHVRTHTQERPYPCPYCNKAFSR
SDNLAQHRRIHEAQQDGQQPLPTHDEDLENEENELGSDRESSPGEAIPSSMANVMGLTSMPSTMPLTSSMPSMMAPQMIAPQLLQQQI
>**HOMEO_An0001 (ANID_00885)**
MEFFDFNEAASGSHVPDDDVASDHIEMDENDVVETYQSLLQDRSEIPDFLPGQSASEEVMSETPDPEGIYPMGRAKEPCDFCRNMGLDCFIAKRGVMQKS
GCTCCISLYRECSFTQTMPQGRFAGVDTLHPISENIYIPTGGLTGKKALKSFSGIAEDVDARARKSSSRLSREAVRILKAWLNDHSDHPYPTEEEKEELK
LRTGLKRTQITNWLANARRRGKIRPSPRSSSPVAGAIEIPRQPVIDHTLMTPLERWKYSPPENEPAALSNILRALEDTPLEIRQGSHSGHVRSQSRRTGS
SNDSSHANSNVFDASSLVSSHDASQSASHSSVSDLSFASAFSHRSSLGSFGSMERKERRRRRKPSLPTNTFNQQKAKGARIYQCTFCADSFQTKYDWQRH
EKSLHLALEKWTCAPHGGVAFINGANRCVFCMAVDPDNDHLESHNYSTCAEKSSAERTFYRKDHLNQHLRLMHNVRFHPSMDQWRSHTTEIISRCGFCGI
TLTTWKGRADHLATHFKNGADMVQWKGDWGFEPVVQDLVENAMPPYLIGHERNTLDPYKPSAHKTAPGLAVPMDANCYERLRMELNAYIQESVTKGIVPT
DQMLQDHGRRVIYGTDDPWNQTCADNPVWLSVLKRDAGLEPAQGSEHIQFSNLGMQPPYASQEGLRRPPACARQPYGSGYPTSGFQSPAIPGTGRSSAAP
SIPGSSAGSFSGSAGMFTAPGPSGLSLDWGSNASAGVSSFSTPLSTSADPFVQMGFDPEFLQQLNHRYEEEVPLESLQGLSFGVDDGGGFDSIDPVGTAA
VTSSATSPIPIPASKQPDIRLPDAVPPDLHFEGTGRTGIYMMNLGYFR
>**HOMEO_An0002 (ANID_01217)**

MNYIHHPYPFAGHPSVPMEQHLAYDTTMVHPTMMHHPMDGYYYAQPPFDMVDYYHQPMMDYEEYAENLSRPRLTKEQVETLEAQFQAHPKPSSNVKRQLA
QQTHLSLPRVANWFQNRRAKAKQQKRQEEYERMQKAKAEAEEAAKRKSESSVPESSDSQRSAEAKDEKKQDDSKAPTPKPSKPASDDQKQSEAPAESNHQ
QTRSESNRVASLASLQRAMDAAAQYQGGQGTTSMGGSGSVSPTTSLPNDADSAVWSSVNSTNGELSVPGLENSQSFSDYRSASDAGASYNSMQFALQADA
ANARRASRSLETGQTARPGKKPARSLISLPAETDRGLPRVGTSRSTSMLSTSIMSPTTRGQNYGTVKQSKSAQNLGSRYAGVRKPSAQRSPLNLSTFAEA
GVLSSAKTELSTMLQPVTTNSLAPPTPLTPEDLHHLLPTTPSTDGYCLSAQPTAHLFPTTQPMQINIASPPATPLGMDIMSSYPYHSVAPPMSAPANFTS
FPDYSCDGSFQGRNWEATSMPSPEVPFQSQCHQMNFSSIPYDHALDQSQSENGPSQSPFGDADIQAPGDASKATEFHLYEFPDQEEAHRFVAQQLPNQKP
KAYTFADNRTPTNFG
>**HOMEO_An0004 (ANID_02020)**
MAIPYATGLPADNQPLPSFRELLPPHLHEEIESTSYFNSQHNSRQPRERPASSHELGLNSVPREHASSRSSRPSPVLPPIRDLQSYPDRATGVYPDPRGL
PPPPEITARPVGPHGYPHAAPAVPGPLADRNADAYRGVPPMHGQVRYHYPSMAYQSDPDHASVPSLSHAPQSNFGILGDSTDARNRRRRGNLPKPVTEIL
KAWFHAHLDHPYPSEEDKQMLMSRTGLTINQISNWFINARRRHLPALRNQRRTGGSDLDERQSLSDMEQTSPEPSPHRRL
>**HOMEO_An0005 (ANID_02290)**
MYSQHGAPMAPPQKPETFMLSNEAQQSLPHDAQVALQQVDNLKYFLLSAPVDWQPDQLIRRFLLPTGDYISCVLWSNLFHISGTDIVRCLAFRFQAFGRP
VKNSKKFEEGIFSDLRNLKAGTDATLEEPKSPFLDFLYKNNCIRTQKKQKVFYWYSVPHDRLFLDALERDLKREKMGQEATTVAVSEPALSFEFDSSQSL
YEQLTKAQQANSSSFAAHASTTYGQSASPVVRTVDAMPPPQMAPQMAPPSIPLLTDESANSQMYTSIPMPNPIPQNLIKREADYGAIQYDRNGMPIARIH
QRHSSMPTFVEYSPAPSFVSSQYEDYSNRGLSFEPVTPPQHSSHIGPEPAYIANEDTGLYTAIPEMSTGSGFNPMMHLPPSNLASAHFPTPARTFHSNVY
SVLEGSPTYKQRRRRSSIPPGAANPVTTGTHSPAPALSYAAHKPSDLRRSVSSSVAPGDTDDSRHESLHRSVNSTYTATLPQKNLMHEMSRNGTPLSSVG
EHREQSSIPLTQAEDLPALPANGTVESGAPNGVGHKSDRYATGPVRRARSATMMELGPYPQKSHSCPIPSCGRLFKRLEHLKRHVRTHTQERPYPCPYCN
KAFSRSDNLAQHRRIHEAQQDGQPLVHEDDLENDDNESVSHDEDESPSESVHPAVPGVHGMTSMPSSVALQSTMGSMMGSHMIAPQLLQQQI
>HOMEO_An0006 (ANID_05048)
MSDPEPSAVILSSSPPSTQNSDSSTASSSYAFLVHSQKTLTQNLPPRVDNKLLARQKRRRTSPEDYAILEAEYQRNPKPDKISRASIVSRVSLGEKEVQI
WFQNRRQNDRRKSKPLQPHELLPSSQSDDSKTKCDDSILVEGSSRLEQDHDHERAHGSRPSMPEGDSFASDAVDKDERGQVEESSQTSVAISESPNSSQQ
AADSSLVELNAMQSPIDASSNALHPNSAKRKRSGSDLGETNKESATPSMLDSLKSPPSLRLSLSFDGEAMVRKQGELTPSPPKGRNSLRISMSADGQAVI
RANGEPSPSKNRISMFPTRTPRLAGLRRSNSAVVLGTPRSLEREKSFGRSRDPRNWESIFDTDARSALATPFSSQSTPKSALLSGGKRSLTRSLSARHPS
LLTPNSDTQGTPITQAMRQKRQKLSRTVSSLGRLESGRKALGEKFPSTLKNSKSAKDDLELEAGDSDKENWIPGTRVSQSRRRTVSSHTHRPVLKDSNGR
DGRPHDASTSGRSRWSQPSHRKTVGPSKSLSMSGLEGDVSAFMGGNAASQEEDLDCIQGLLSLSQGAWR
>**HOMEO_An0007 (ANID_07012)**
MSVSGPCAWVSTIPSPAAAHPSSITSSWASVSWNSEPALTKSRADLSALSGLGKTVGEPDKAPPTEMQPAAFKAENAFSTQPAAGSSNSVQYSGVDVLSK
GLENRNTNAHSDSSLTANPERQNADIQGSSESQTNDELSSKVKSEEVHEDEKDHGEANAGSSDDDGSIADEKKSSQDVKLDKKKMKRFRLTHNQTRFLMS
EFTRQAHPDAAHRERLSREIPGLTPRQVQVWFQNRRAKLKRLTSNDRERMLKSRALPDDFDTTQVLRTPFGSKGPSEASVESLRLALYTQTKVSQGLLTE
GLPRLNDDDYVISPLSSASTSGPGYPPTSSDRGFENYQNRGAAATVPDLRSSRANFPFPRSSSFSEPSFNTSLQYPGRFSRPGDAVGHPGISYRRPMDYV
MNRPANGMMVGYGQHRPLEGSVSPSGQQEAQLTYGMDNTNQIHNYQPPLSMPAPKSYGGIEMNSHMHHPGRSMPTLQHLPVSETPDYRPYPYEHHPYSMG
TGIPFTQANASSLSLPASFSSEQGHVAVSSPADDRMTTSPQVMDPLRAKYSQGYDYANYL
>**HOMEO_An0009 (ANID_09328)**
MGNDKGATAHVTIFSLPRMTLDSSDNLQSGLLSASLILNDEFTGIWPSPDIGLTPPETPIFANGSRSTPFDDSSAACLDGTPGTVFPGPGRELPNLTEYK
VLQPHGRRGMGVGILKKWFDQHCDKPYPSKEEKTELASRSGLTLTQISTWFANTRRRRKNKSPCPNPLLTSTRHSRRPLEGELSLMSPMERWRNSPPEAE
GASLDAIMGAVVSSDYPAHQDPYIRYSEHSTPSDARSAASSNASGSAVSNNSVSSTHSIGSHSSAGSFYRFYSAEAPRRRRRRKRATVYPKRAAKTQEPR
PYQCTFCTDTFRTKYDWTRHEKTLHLSLESYTCCSSGPTYTDTAGSQRCCFCDHLQPTDTHIESHYYSRCQEKPVVLRTFYRKDHLVQHLRLVHGVSQFL
PGMEGWKMQVDNINSRCGLCDTTFVSWSARNEHIGQHFRNGALMKDWKGSRGLDPSVAFAVENAMPPYLIGIEAVTPDPFSASRLPNQSYPGDSMPTTWP
QLQSGAKRSPFNHFTVDLTNFVREAQFLNQPITDTVLQTAGRRILFDDEDTWNQTPADNPEWLQMFKRAVCLECEGEDNLSSGRPPLSVEDFDFFPSSAE
PWAVWNIFDANLRTGESTDAMELGISLPWRDSGTGAASRNSCTEDKRTEPESLPELTSASLVELQQCYRH
>HOMEO_An0010 (ANID_10334)
MSLQHLQAEDERGVTAPAAPAGGGWPTEEDIFFDWELYGIQDPSCNPEFLPYEQASRSTGTLDFTATQFEPQRASLSGGDSASAIASNPEPEGWGCLQVG
PGTTSTPIVPSGFNNVAEWLDGAYRPPTPCDHCRRHRLQCLVLRRTSHNPNPVPSCSSCVGLFRPCSFGRGEKRQPSGFETLSPVLGHLHGVIEEGNENE
CGERKLDSKESKQFVRKGARVLRDWFYQNEHCPYPSEEEKARLAAETGFSRQRISTWFANARRRHKQQKQARTSTRVFRAGSPMPTSELASMTPMERWQA
SPPDEEPVSEAMIREAIATSSGSDVSGCRSRFESPLTDFSSGLEGSSLASSVSSFGVLASDASDSSSSAWSYKSGDGPLRPRPYRRPSSGRRGGRKRVTE
DGHYQCTFCMQSFKKKHDWSRHEKSVHLPLDVWICTPNLSELEDTNIPFGKCRFCDHQSSSPEHWESHDFRECASKPLPERSFSRKDYLWQHLRKFHGCT
RYPVPNLEAWRSAQTDIQSRCGFCAASLPSWSARADHLAIHFKDGCRMSQWAGDWGFEFSVLANLRNAVLPMERSLLSMAST
>**HOMEO_BDE0001 (BDCG_00961)**
MEYFDFEQAALDDDVDSNCQVIDESDFIEKYDTLIVEDAPVLPSPVPPTTAQPDNSQEQEALPSILDRGVFPVTRAEEPCDLCRSRGLDCFMAQRGVMQN
NGCTCCISLYRECSFTRTKPHENGLDTLHVVSEDSYVPIGSFTGKKLLRSFHGGTCSTTFEETEPSGRKSGSRFPREAIRVLKAWLTEHASHPYPSDKEK
DELKLKTGLKRSQICNWLANARRRGKVRPSLRDRSPPPTGALDIPRKQLAPGVDISELTPLERWKHSPPEHEPASATDIIRAMANAPFAPERNPSSGGEK
NPSSAGRFRAHSRQTGSSTDGSGLSNVLPARSVSSYSLDTTQSSISDMSFASAFSHRSSRASFNSFDGKDRRRRRHKSAVGQNAFQNARAARIFQCTFCT
DSFSTKYDWRRHEKSLHLALEKWTCTLQGGVVTINGAVVCAFCKSPNPDDDHLESHNFKACQEKSLQERTFYRKDHLSQHLRLMHRVKFGSWMESWRSAT
TEIRSRCGFCSAIFNTWKDRVDHLSIHFKAGNDMSQWKGDWGFEPSVQRLVENGMPPYLIGEERKTPDPWVARPPAKKSSYDALRRGSNPGSSAESGDIP
VPGDANCFRRLGIELSAYISRQVASGIIPTDSDILSEARQIVYGSDDPWNQTCADNPIWLSILKRDNGLCEPPGVENIQLDDLGMQPPFAAPGLSQPPMH
KFLTIPPNLTNAGVNSSGFQSPAFYSTSFNSGAPSIHGSFMDSATGSVGVSSAGFPSGGWPGLTTSNALSSSAPVSAGFDLGIQNTHHYLQQPPGSVQGD
LGAHFSDMHIDRFEPGGHYRMGNNEGKFGAIDRHSLSEAPSYFEVPSTMPIDIPSSVAPVSGTHIASVWDDSWVQKFDYIIEE
>**HOMEO_BDE0002 (BDCG_02041)**
MYSQHGASMAPPQKPETFMLSNEAQQSLPRDAQVALQQVDNLKYFLISAPVDWAPDQLIRRFLLPTGDYVSNNLFHISGTDIVRCLSFRFQAFGRPVKNT
KKFEEGIFSDLRNLKSGTDASLEEPKSAFLDFLYKNNCIRTQKKQKVFYWYSVPHDRLFLDALERDLKREKMGQEATTVAVNEPALSFEFDSSQSLFEQL
TKAQQANSSSFSAHASTAYTHSTSPMGRATDSMPPPQLPQSMPVLQEDTSNQGLYNSMGMPNNSLTNGMSKPEQDLGQFSYDRNGTPVSRIHQRHTSMPT
FMEYSPAPSFVSSHYDEYGARGISFEPITPPQHSLHLGQEPAYIANEDTGLYTAIPDIGNPTGFAPMMQLPPSNLVGPQFSTATRTFPTTNVYSVIEGSP
TYKQRRRRSSIPASITHAIAAAAGQSTQSHGHSHAHAHAHAHAHAHAHAHAHSVTRPSELRRSMSRSVPAVMEGEESNNDSPPGLTNSYTSTVVNQKDLL
HELSRTGTPLPSLEESPPEHPLSLIQAHDELSSLPNGDQIDNAIAASSANGKGDRPGPVRRARSATMMELGPYPQKSHSCPIPSCGRLFKRLEHLKRHVR
THTQERPYPCPYCNRAFSRSDNLAQHRRTHETQQSGQQIVNNYSDEDREQEHEEQEFGSLGDTESPRSSSDPNSHSHNHSNSHTNRNRNTNNTDHHHHSS
SNNNNNGSNNNNTKNNGNNNNYMPTSMVNMANVTSMPSSMVMQTSLPTMVTPQMITPHLLQQHI
>**HOMEO_BDE0003 (BDCG_03256)**
MIDAEELRPAPSQRNKITMTLPERIPKASDKSWRIAGQDLLSQKSHQLKVIPATEPVIFLMISLNLFDIYPWMTGDNEIGREIQDFPMILSFYTKSSYSN
LKRSHQYKV
>HOMEO_BDE0005 (BDCG_05449)
MSYLHHPFPFGGHAIPVDQPVGYGVPIRHPQLGHSVDTYLLPNAPIDLADYYHQAALLEDFEQYTENLSRPRLTKQQVDTLEAQFQAHPKPNSNVKRQLA
AQTNLTLPRVANWFQNRRAKAKQQKRQEEFERMQASEKDEQWQNNDAPQKEGESKQQSELPESSTTPTQRPASISSCSSPLSPAKQEEQQQASDFTTKTG
CPESPQKAMNVSMPSSAQFNQPVENNGPDRHESSAITYHLSQSPSGKNGGSTFTSSLSGWNGMEDNHAIWPSPQGAEDSFDFGHLNRHHDNPLEIRVQGS
PHFPSIQFSHEPDGWGYHSMPHLVGEQRHNSQDSGDGFYQIPFHALQSPLYPEEPRRDSYSSEQSELADTLFHTEINNVIELSPQNTPHLNLAQLHHQAE
PSTNWRYPEKEVDIAGRRKRPRPAAIGTSSMSRSYGPSSMSPTTRIHGMGAGHVLRHAKSTQNLSPSHTSRYPGIRKASAPQRSPLGITSFAEASLFNCA
NATDMMSTVPGLVTTTLAPPTPLTPEDLRTLLPPTPNDSQYCVSPTDDMGCARFFPISQPMQVHIESPPTTPLHLGVPSHLQYQSMGVPMSASSQRTTFQ
EYALSVPTNPMNGGLWSDVSSMPSPETSHLQQPTIHMPQPTHISPITYEESFDSGNPALVEDTVTSQSESPQCTVKSCGTPTTSSPQGSTPGSQKVTEFL
IQEFPEQQEAHRRAAEQLPPQKPMNYTFSNHTPNDF
>HOMEO_BDE0006 (BDCG_05795)

MSRKVESEEESKNMVLSYSSEFRNGGQTQTLPPFRDLLPQYLHEEIDSTPYSAPTTPHHQGSPSFISSPVHPRSILGASLSRSHSATKIDSYNEIPLRTD
RFPKYQPHGGVPSLKRQASDTIGPSTSISNSCFNSGGALPPISDIHSIPTDRSPYNRGQVGLEPNSLPPEYQNSPSQNTPSFDSRPTSSHLTGCQPLHVI
PPNPILPSEPLRLNHPYESPRYGSHPHSFRHEFDYSPISTSSLQDRSFVTQAEPSDSKNKKRRGNLPKSVTDVLRAWFHEHLDHPYPSEEDKQMFISRTG
LTISQISNWFINARRRQLPALRNQVRASESDRSGHRQSPLSDNEQTSSPSSM
>HOMEO_BDE0007 (BDCG_05805)
MSSTTEPVATSITARATTMPHDPTNNISINSSTGVTPNNTNTNNTTPTISTPSPSSTNASAVSVSSATQNRASIRRPPRKSTLTQQQKNQKRQRATQDQL
VTLEMEFNKNPTPTAAVRERIAEEINMTERSVQIWFQNRRAKIKMIAKKGIETGEDCDAIPESMRQYLALHFDPSKAHARNLFHKGPGYGPNEMHEPTSS
GKIVIHHFACRSLRIGSWRRVGQNAMDLVIFYSPEKSCMTYYINNDSAGYKIEYPFSFIKNIVLESGDPGPSADGTAPKPGGLVIELNRPPNFYMDSSNS
GGFYQCRDFTEDQQATKSMVHYLGGHPKVLSVQLAKLVSLESFQNRLMQYDFNGYAVSAPVSPHIVHRPASQPNHLARPNSSIYQENSNHFGMSLHPGRG
HKRQRSRSVPIPVDFAAMGHHMPSFHSQQHHPPAQQFHPDPNIFAPIPQGRMPHIPAVSNDLRLDNPTGYGMDFQSYPMPAVTTTHPTATTGTTSSEFAS
PPFFATSAPPNQMPTQSQHQQQPPTAPAPTVGTPYSLPFLSPSPMVDAPNMMSQQQLPPPPQEQQQQQQQHSPSPLSHVSHATAESMIADQSPPSMTKLQ
LHPSSSASADDIFTLSVADHDQDHDDHMAGMSGLGAVGVVDEDGSDDATGMLLSEMYSKQNLNQHHNHSHNNNHNMSMSMPMSMAMPMSMPLHQHHHSPPM
EMDDDSFVLALQGLSGDHDHHQQQQEQHALGHGHSPAGESVADYHGMLPFETVDPSSLAATAAEA
>HOMEO_BDE0008 (BDCG_06899)
MSDPAQPSPAGVNPSTGPPAGDSTNNNYAFLVHSQKSLTQNLPPKVDNKALARQKRRRTSPDDQKILEAEYQRNPKPDRATRAEIVNSVSLGEKEVQIWF
QNRRQNDRRRSKPLNQEDLRAPKSSGSDQDTSNGDNPTNPKSQSSQLPKAGQGTGGQISTDARREYICISPGSSYRSPMADLYGAAFSSQTSNSQTTVLS
QHEETHVEVGKQKESTGGSKEGSATLINEKLHIESISRKRSRSEPEAPNTNKYSPPTSSTFIPPPLRISLSFDGEAVVRKEGENTPSPQKPLDSIRISMS
ADGEALIRAANEESPIKNRTSLFHNTRPAFGGLRRSISAFPLGALKGIKEQQDPKPFGRSRDSRNWELYCDTDARTALQGSKSSLLSSTSRSVKLSRRKS
DLGHGRKALMPRTNLPNTLPLTEEPEGKRKKLSRAMSSLARLETGHKVSTSKMGVGEGAMDYHTGDSDKENWIPGTQMSAARRAPSNKQKARRGVLQKPN
QIQNTSNRQVTGSNKTQRGYQGRLLNPNPNTTSEDADDGTEKSYGELGEISRLSQDEDLDCIQGLLSLSQGAWK
>HOMEO_BDE0010 (BDCG_08683)
MSTETSSMRFDLRQSGLNVLGVDRETAITYFYGHHYNFLRLFTSLPKWVRASKRLVLVGADIPPFRSQGKSTTRFDIQNLPPRVLPPAAAKPPGLILYPA
MTRLTLVGISTTGTRPTAAQPRNFRPFPTVMNDRDLLQIDPIDRKRFELEPRAHLSVAPASEIKETQNEIRNIKTILSETETEYLEGNSEFSMPSTSANW
ISGAYLTSNPVESHSIKTNDNDAEEGDCENELAIGICDDQLERESHEGNGQRQPGGGGHAKVPADWQTGKGKMKRFRLSHNQTRFLMNEFARQAHPDAAH
RERLSKEIPGLSPRQVQVWFQNRRAKLKRLTNDDRERVLISKALADGFDIARSIHSPYGSWHQSSHTLASPGSYLNANQEGGEEDILTPLIVDIVGRLSD
EDYAISPLSASSNYDSYFPSPASASASGSELDMPPITIGGDIASHCPSFSNPQTSSFQYMSPCTSPGSFSHSTSQTSHHHIQAMEQQDTTRQRAGSLRSP
PRASISSTHAYMGLDMPCNTHSYDNITSQTFMDTNVSHARDDGLQSAPLPDMHILREDQLSPFSTTTTTITTVGIGPFDISYPERNSSSLSLPASFIPY
>HOMEO_Hc0018 (HCB01829.1)
MCDPEQPSSAGAKAATVPPLKDATNNNYAFLVHSQKSLTQNLPPKVDNKALARQKRRRTSPDDQKILEAEYQRNPKPDRATRADIVNRVTLGEKEVQIWF
QNRRQNDRRRSKPLNPEDFKVPKPSGPDQDASNGNNPVNSESQATQPSKSFHGADRRTSTDTRRVNLSTSPDSSFGSESAVEHESTLSSQPPNSQTTVIS
LQREELRDEGQTGGSKEGGATLIADELHIEPFSRKRSRSQPEAANTNKYSPPTSSTFIPPSLRISLSFDGEAVVRKEGESTPSPKKPLDAIRISMSADGE
ALIRTASEESPIMKNRTPLLYNTRPVARGLRRSISAFPLGALKGTKEGQDPKPFGRSRDSRIWELHCDTDARTALLGSKGSLLSSTARTRKLSHRKSDLG
HGRKALMPRTNLPNVAPLAEEPTGKRKKLSRAMSSLARLETGHKGLISKMEEGVMDCHTGDSDKENWIPGTQMSAARRVPSNKQKTRRVGLQKPNRLSNE
QSASYRPIAGSNKMQRGHRGVLLHPSPHSTQEGGNDVTGKFHGPPDEISQLSQDEDLDCIQGLLSLSQGAWR
>**HOMEO_Hc0019 (HCB02177.1)**
MTAHNGTTNDVNSTSGVTPDNTTSAISTPSPSSTSASAVSGSSATQNRASIRRPPRKSTLTQQQKNQKRQRATQDQLVTLETEFNKNPTPTAAVRERIAE
EINMTERSVQIWFQNRRAKIKMIAKKGIETGEDCDAIPESMRQYLALHFDTSKAHARNLFDRYPGYGPNEMHEPPVSSGKIVIHHFACRSLRIGTWRRVG
QNAMDLVIFYSPEKSCMTYYINNDSAGYKIEYPFSYIKNIVLESGDPGPNADGTPPKPGGLVIELNRPPNFYMDSSNSGGFYQCRDFTEDQQATKSMIHY
LGGHPKVLSVQLAKLVSLESFQNRLMQYDFSGFAVSAPVSPHIVHRPASQPNHLARPNSSMYQENNNQFGMNLHPGRGHKRQRSRSVPVPVDFAAMGYHV
PSFHIQQSHPSAQQFNPDPNIFAPIPQSRMPHIPPVSDDMRIDAPPGYAMDFQPYPMSATTTTHTATTPATSEFANPPFFATAAPADQKPIQMQTHQQQP
NTSTQLLGSPYSLPFLSPSPMVDAPNMMPQQQQHSPSPLSHVSHVATEPMIADQSPPSVTKLQLNPSPTPSADDLFSLPLADHDAKQGNDEHMAGMPGLG
PVGVVGEDGSDDAAGMLLSEMYSKQDLNQNHDHNNQDMSMSMPMSMPIPMHHDHSPPMEMDDDSFVLALQGFGADHDHRQQQQHGLGHSPPNESVVDYHG
MLPFDTVDPSSLAVTAAEA
>**HOMEO_Hc0020 (HCB02186.1)**
MFRKAKTVEESNNMVVSYSNEFRTGGQTQTLPPFRDLLPQYLHEEIDSTPYLSPTTPHQQGSSSHVSSPVHPRSILGANLSRSQPVTKFDSYEEYPSRTE
RFPKYQPQGGVAPLKRHASDTNSPSSTIPSSRVNSAAALPPINDIHSFPANRSLYTRGAQPNNFSEYHYSPTRESPSFSSRPPDSHFTGCQPLHVIPANS
LLPSEPMKLNHQYESPRYGSYPQSFRPELDYSPISPGGFHDQSFVTQTEPIDSKNKKRRGNLPKSVTDVLRAWFHEHLDHPYPSEEDKQMFISRTGLTIS
QISNWFINARRRQLPALRNQARASESGRSGLRQSPLSDNEPTSSPSSV
>**HOMEO_Hc0021 (HCB03346.1)**
MYSQHGASMAPPQKPETFMLSNEAQQSLPQDAQVALQQVDNLKYFLISAPVDWTPDQLIRRFLLPTGDYVSCVLWNNLFHISGTDIVRCLSFRFQAFGRP
VKNTKKFEEGIFSDLRNLKSGTDASLEEPKSAFLDFLYKNNCIRTQKKQKVFYWYSVPHDRLFLDALERDLKREKMGQEATTVAVNEPALSFEFDSSQSL
FEQLTKAQQANSSSFSAHASTTFSHSTSPMGRATDSMPPPQLPPSMPVLQEDTSNQGMYNSVGMPNNSMNNGMSKSEQDMSHFNYDRNGTPVSRIHQRHT
SMPTFMEYSPAPSFVSSHYDEFGPRGISFEPITPPQHALHLGQEPAYIANEDTSLYMGVPDLGNSTDFTPTMQLPPSNLAGPQFSTATRTFPTTNVYSVI
EGSPTYKQRRRRSSISSSITNAIAAATGHAQQHSNPSHGHSHAYSVTQPSELRRSMSSSVPAVMEGEESNGDSPPGLTNSFTSTVVNQKDLLHEISRTGT
PLPSLEESPREHQVSMIQGPDELSSLPSGDQLGKSVAANSANWKTDRPGPVRRARSATMMELGPYPQKSHSCPIPSCGRLFKRLEHLKRHVRTHTQERPY
PCPYCNRAFSRSDNLAQHRRTHETQQNGQQVVASDSDEDREQEHEEHEPAGSSQTSSSDPHNHSHTIHSNNTNRQHRSSSSSSSSNNNSNNNNNGNNNYM
ATGIVNMANVTSLPSSMVMQSGLPTLVTPQMIARSGLV
>**HOMEO_Hc0022 (HCB04274.1)**
MKDHSPLQIDVIEQKEQKVHLSVPPVCESTDTLALHFKDNGDSEAQNEINNKRSVLPQAKMKYLEGEPEFSMRSTSTNSTSGASLVFNRIESDNIETNDD
DTGDDININIGVDQFKTHVGHEGQGQRQSVGQSLAKATIDRLKAKRRMKRFRQTRFLMSEFTRQAHPDAAHRERLSKEIPGLSPRQVQVWFQNRDDREKA
LIPRTLPDGFDITEDIYSLYGSWHQSSNNTLTPAGIYSNPIQEGGDIFTPLISVITRKPCDEDYTTSPLSASSACDGYFPSPTSALATGCHFPAVPDRQF
EPNAPNQPPSRLHLNMACWKRGDHGLQSDPLPHNTAHEDQISPLDSTFSSNPCGVSYGERDFSSLSLLASLIPF
>**HOMEO_Hc0024 (HCB05524.1)**
MEYFDFEQAAQENDVASNFPVIGESEVAGKHDSLIVKDVSLLPAEPPYTEPDIPQKEDPSSNLYPITQPEEPCDLCRSRGLNCVMAKRGVMQNNGCTCCI
SLYRECSFTRTKPHEKGLDTLHVVSEDSYVPIGSFTGKKLLRSFHGGTSSTTFEETDPNGRKSGARFPREAIRVLKTWLTNHANHPYPSDKEKDELKLKT
GLKRSQICNWLANARRRGKVRPLLRDISPPPTGPLDIPRKQLAPGVDISELTPLERWKHSPPEHEPASATDIIRAMENAPYAPERNLASGGERNPSSAGR
FPPHSRKTGSSTDGSGLSNVIPARSVSSYSLDTTKSSISDMSFASAFSHRSSRSSFNSMDGKDHRRRRRHKSAIGQNLFQKARAARIFQCTFCTDSFTTK
HDWQRHEKSLHLALEKWTCTPQGGVVTINGAVVCAFCKSPNPDDDHLESHNFNACQEKSLQERTFYRKDHLSQHLRLMHSVKFGSWMESWRSATTEIRSR
CGFCSAIFNTWKERVDHLTAHFKAGTDMSQWKGDWGFEPVVQRLVENGMPPYLIGEERNTLDPWVAKPPAKKSSYDVLRKDSNLGSTGESSDIPVPGDAN
CFRRLEIELSAYIKRNVSQGVIPTDQDILSEAQQIVYGSDDPWNQTCADNPIWLSILKRDNGLCDPPEVQNIQLDDLDMQPPFAAPGLSQPPMHKLLTST
SNLSNAGVNSSGFQSPAFYSTSFNSGAPSVHDSFIGSTAGSVGVNSAGYPSGGWPDLTTSNTLSSSAPVSTGLDQGSHFGFYAQYLQQLPDNVRGNLGAN
FNDMHIDAFEPGGHPGNNSKVSTINQHSLSGAAPSFEVPSTTPIDIPSSSVPVSGTHIASGSFEPYFQNIFDYHFDMS
>HOMEO_Hc0025 (HCB07673.1)
MSYLHHPFPFSGHHAIPVDQPFDYRAPIRHPQLSHPVDTYLLPNAPIDLAEYYHQAAALEDFEEYTENLSRPRLTKDQVDTLEAQFQAHPKPNSNVKRQL
AAQTNLTLPRVANWFQNRRAKAKQQKRQEEFERMQASGNGEQWKNNDTKQKEVASKEQSERLESSATPTQQPDPSSSSLNPSEVENEKQQQASNSISKPG
LPEPPQKAMKATMPVSAQFSQPEEDNVPRHGPSDIKYHFPHSSLGNDDCGTFTSNLCGWNGIEDNHAIWSSSQDIEDNIVFPHLNKPHGNPLEICVSESQ
QFPCIQFNHEPGEWECHSMPHLMGKQRNSSQDLSEGFHQIPFHALQSPLYPDEHRRGSSSSEQSELADTLFHTEINSMIETSPQNTPHLNMAQLRHQVDP
TTNWRYPEKEVDIAARRKRPRPAAIGTPAMSRSYGPSSVSPTTRIQGMGAGHVLRHAKSTQNLSPNRYPGIRKASVAQRSPLGITSFGEANRFNCANTAD
LMSTLPGLVTTSLAPPTPLTPEDLQTLLPPTPNDSQYCVSPTDDMGCARLFPMSQPVQVHIESPPTTPLHLGVQSHLQFQSMGVPMSTPSQHTPFQEYQL
SIPTNPMSGGHWPDASSMSSPETSHLQQPTIHMPQPTHISPITYGESLDSGNPALVEDIMTSQRESPHCAVKNCNTPTASSPQGSSPGSRRVTEFLIQEF
PEQQEAHRRAAEQLPPQKPMNYTFSNHTPNDF

>**HOMEO_Mc0001 (MCYG_00398)**
MEYFDFDEAARQNGETFRYIGVAEIDRCAPVPADCDGAVSPSTPPFSQPGDIQRPSNLDGVYPMHRVRDPCYFCRFMGLDCVVAQRGVMQNACTCCIALY
RECSFTRALSKGSHIGTLRSEPEDGSTKRSPSIQQLEKPSNSNSEDIAQQPRKPGVRFPLKAVKVLKSWLSEHASHPYPTDAEKDELKLKTGLNRTQISS
WLANARRRGKVRPPPRSTSPYPGAVTIPGKRLPPGVDISELNPLERWKHSPPENEAASARDIIQAMATSSFISHAESDFPHLKSHSRQTDSSNDDSFSNK
FTAHSISGYSADTNKSSISDMSFASAFSHRSRGSWNSIETRERRRRRHKSAAAPNPFQKSRVARIFQCTFCTDSFPTKYDWQRHEKSLHLALDKWTCAPN
GGVITSSNGQLVCSFCHHIDPDEDHLESHNFSTCQEKTVQERTFYRKDHLNQHLRLMHGVKLAPWMDSWKSTTTKIKSRCGFCSATFSTWKERVDHLATH
FKAGTEISQWRGDWGFEPHIEDCVENSMPPYLIGQERNTLDPWVASKAFNLAKYGRNFNKEHKVAPSEVSGVSPFGMPFRTDASCFRRLEKELSAYIARL
FDQGIVPTDKLLQSKARLIVYGSDDTWNQTCADNPTWLKVLKRNNGLDDSSDTENVQLEDLGMQPPFAADGGLRQAPGNSTKSPQALSGTLSPPSGLGST
TTSNPESVSGSARNHSDLSFKGGGGNSVHNFTPSPGAHSPHNLNFPSPVSANGVPNDNTSVDPLLEMGFDAEFLQQLNDGYGDIGRVIDGLELQSLGYAG
NNMHNGAFNVKNNHNFDNVSTTDSTPVTPLSGMAMTSPEALFGIVSSGGSVEPGSESYRGEIN
>**HOMEO_Mc0003 (MCYG_03024)**
MSDSRQTAHVGDAPVSTVATEADSGSYSFLVHSNETLTQDLPPKVDSKVYIRQRRRRTRYVFLDPSSVGRDKKVTNYVYCSPEDHAILEAEYRLNPKPDK
ATRASIVSRVSLGDKEVQIWFQNRRQNDRRKSKPIHPNELSPDTREPESSQKAVDGSPPNTQDQNSNSSAPHKHYNHTLDTPSQSLPNSQCEHATISSQL
ESSQTSTSSTKSQTDRCSFCSPSSEPIVPPQLSSGKRKRADSGVSGLGIGGLEHGRRSLGSTHSPSLRLSLSFDGEAVVRGEDEKTPSPPKIRDSLRIAF
SADGEAVVRTAGEPSPSPPRRHTSTPVSLQSRLRSLRRTSSAVSLGTPGFKSPDPANIFGRSRDSRRWELYCDTDARSALSSPHIGAGPALDRPGGAKPS
HRRTSNANTRILSPCANLPNTLLPDGPPQKRKKLSRAVSSLGRLETDPLDTSSTTKLSSSQRNRGQRSKPKTDFHSGDSDKENWLPGTQTAGPRRRRPQS
TAQAQNRVLQTSGDARREGNRIIGRGSMSPRKGQGRHKDDEYGEKPSVPGSGSNQVEDLDCIQGLLSLSQGAWK
>**HOMEO_Mc0004 (MCYG_03787)**
MNYLQAPYQYSVPMDQPMGYGMPISHHPYDQPMDQCYVPYHQNDIHGFYTTGAIEEYEEYVENLSRPRLTKEQVEILESQFQAHPKPNSNTKRQLALQTS
LTLPRVANWFQNRRAKAKQQKRQEEFEKMQAKEKAMAAEGSENQQQQSESSDEQQKSEQDQKNSILTNTRGATSSQGEHGLQTPAEEKPEPRFDAAGHQS
KVQAEVPNPEPIKLIPSPPMKEDQEMSGNVVDMHNTAQPPFSQPGVDSHFQQWGPTPVPQIEVRVGSADAHPVATSAPIMELDSNGYWSPDSSGIVNQPF
TDLLCNGSSPEELPCQVQPDTTPFSQSFMQSDMPLYSSQNLVNQRHVSLPMEQGDISPRSGPNSDSELTHSEGRQLPRLHISTSDNAIGLAARRKRPRPA
AIGTSGLSRALGGPPSMSPTRRVSSAAWGGVRKSSQLAELSPRYASVRKLSGSPRSPFPYSLEGRQHALSNTDLAVPSSTTSSIPPATPLTPDEMQYLLP
PTPIDNQYCLSPQEEMGYAHSFPTSQSMNFDENQESKRQPPPFVMVGMPHAQSYQSFTEPMSAPPNFTTFNELIPDCGQQQQQQQALTSSEADPSLNVIH
MPRPTHISPVAYDDQMQGEQQVEDSAATEDWQGQGQQSTQSTSPNSPISESGQGYNSTGKGASTEFYIQEFPQQDEALKMAAQQLPPQRARTYTFTNQTP
NDFYRTAIFPPI
>**HOMEO_Mc0005 (MCYG_05206)**
MAMETSSVTAATTTTISTAMSTTATPTISIMSDVPFATSAGISTPTDAMSLATPPPTATSQASSTSSSITTSTTTAAPMASANATASDSPSTRPASRRPP
RKSTLTQQQKNHKRQRATQDQLVTLEMEFKKNPTPTAAVREQIAENINMTERSVQIWFQNRRAKIKMIAKRGIETGEDCDAVPESMRRYLALQLDPASSN
ARNMLGRAGGYPSNEPMMHSATSSKVGAVIQHFACRSLRIGTWRRVGQNTMDLVIFYSPDQACMTYYINNDSAGYKIEYPFSSIKNIILEPGDPVPPLDG
ALPRSGGLVIELNRPPNFYMDSSNSGGFYQCGDFTEEQQATKSLIHHLGGHPKVLSVQLAKLVSLETFQNRHSPAYPFTPNTPFIDGSPQFVHVNRPASQ
PNHLSRAQNRLSVDNHLSVGLHPNRGHKRQRSRSVPGPVDFAALHAMPSFHFQSTNHFQPSTPQHQEHHQPDIFAPIPLAHSQFHLPLNMSEPDLHVDTS
ATQPPPPFTAPPITLSPLELHTACPSSHPHQLWTSKLIPILSTFPSNIRPKQPRTLIRPH
>**HOMEO_Mc0006 (MCYG_05212)**
MTVAYSTVLSDHRSEKLPSFRELLPDHLHDEIEGGVFYTQSNQNWRRGSLQDTMPPPTPQHTDRLPSRLFARDSRFDDVSLRPDRFLGNNAKSPNIQQPH
ISEPKSTASAPHSRPRLSLSEINRSLPPPIPSPSREYLVKVSPNANSNAIYAPMNSRGRNDGELSNYSISSPRSQFSFDSSSFSHSSSPVNMVSPCDCRD
QLPYPASYEQPHACDRGYPQSSFGHFRPASGFCLSECELGDMREKKRRGNLPKPVTDMLRAWLWEHLDHPYPTEEDKQIFMSRTGLTISQISNWFINARR
RQVASLRNQVKARNNSETDGSSSRRNSSPRQSDDDSDYLRTPSEKHSSLP
>**HOMEO_Mc0007 (MCYG_06904)**
MYPHGAPMPPPQKPETFMLSNEAQQSLPQDVQVALQQVDNYIMVDAHNLNSEIFPHLCSSRAWTPDQLIRRFLLPTGDYVSCVLWNNLFHISGTDIAFGR
PVRNTKKFEEGIFSDLRNLKAGTDASLEEPKSPFLDFLYKNNCIRTQKKQKVFYWYSVPHDRLFLDALERDLKREKMGQEATTVAVNEPALSFEFDSSQS
LYEQLTKAQQANSSSFSAAHVSSAFASSHSTSPMPRATDSMPPPQMAPPSMPVVHEDQQSNHAMYHGSVGSDSINGHIVKTDPDYQQFQYDRNGMVSKVH
QRHTSMPTYMEYSPAPSFVSSHYDEYANRGISFEPLTPPQHAMHLSSEPAYIANEDTGLYTAIPDIGSASFNPMMQLPPSNVAAPHYSTASRSFPSSSVY
SVIEGSPTYKQRRRRSSIPPSVTSAITAATNNLHAQAQSQDMHQPQVPVQPHAVHRPSDLRRSMSTSVALEGDPSHEDSPPRAMYSQPSVHHSRTTTPLP
SLEENPVQVSVPISMGETTVSALSNMETLDGTEAMHLMNGHQGERPGPIRRARSATMMELGPYPQKSHSCPIPSCGRLFKRLEHLKRHRRVHETQQSSTT
GSQPPSFKEDEHDNDREQSVAVSQHEQQPQQLQQAPTSNATYLPTTVVNMANVTSMHAMGTHSNLPSMVAPQMIQPQMVQPQMLQQQM
>HOMEO_Ci0001 (CIMG_02405.2)
MPRPPSPPEQSKDMAVSYSSGHSPGHTPKLPSFRELLPDYLHDEIDSASYHLQTGSHQQQHPHHQHSSRLPPVSRQEPPSRRGSGTRHHPGSVGRGQDYD
SSLMRQNAYLEGQSRYHSHHLPQERQYYDDRAPLTPSSRSSSRHLDPSDPRNFLPPLRNYEALPGQIPSPVGRHDDRGYERAYDAERASYASSRSSTLSS
MTHGTAGPYPSAYNSPIHGSYQGYYGGDLESTAHSAYMDTKPTKYDPLGDTADSKSKKRRGNLPKPTTDILRAWFYEHLDHPYPSEQDKQMFMTRTGLTI
SQISNWFINARRRHLPALRNQGRIPDPDRMRPSSSMSEDDPDYDTSPSRR
>**HOMEO_Ci0002 (CIMG_03328.2)**
MEYFDFDQAARDIDTASNCPEIDHQDFDALMNDDNVVVSATDALNDVAEGEKGWEPSFKLGGPWPMYRAPEPCFFCRSMGLDCFVAQRGVLQNRCTCCIA
LYRQCSFTNAEPQQKCLETLHVVGEDSYVPTGSLTGKRALKSYRGPSTGTLLEEPEQRGRKSGARFPKEAVRVLKSWLSEHTAHPYPTDEEKDALKAKTG
LRRSQISNWLANARRRGKVRPSPRHPSPHPTGVPIPGKELPPGVDFADLNPLERWKHSPPENEAASARDIIQAMATAPFHPNGSNDTSGPVRSNSRRTGS
SNGDSSFSNVRPAGSWSSYSLDTTQSSVSDMSFASVFSHRSSRASFGSMDTRERRRRRHKSAIGQNPFQKSRAARIYQCTFCTDSFATKYDWQRHEKSLH
LALDKWTCAPQGGVILENGESVCAFCRYLNPDETHLESHNYTTCQEKSIQERTFYRKDHLNQHLRLMHNVKLGSWMDQWKSATTEVKSRCGFCSATFTTW
KDRVDHLAAHFKAGAEMRQWKGDWGFESCIQRCVENSMPPYLIGEERNTLNPWVAQVPSLSAAKKPNASTAGTSAAQIPVPTDASCFKRLEVELSAYIGR
LAAQGTVPTDAMIQAEARNTIYGSDDPWNQTCADNPTWLALLKRDTGIYDLPGTQNIQLDDLGMQPPFAANGGLRQAPKHSLRTTPPCSGSSGFQSPALP
SSGFHSSGPSHRGSHAGSLAGSVDFSAGGLGQGSLGGLPSSTLGGTLSSSAPVSMDNDPFIQMGFDADFLQRLDDDYEDIGHDLDDLHLEKFGLGDSAGF
HDLGFNDTTFNMTATSAPDQNPLASKLPTSMSYEARYQNVFDHSVAFSEPRASEFRDFGR
>**HOMEO_Ci0003 (CIMG_06050.2)**
MAPPQKPETFMLSNEAQQSLPQDAQVALQQVDNLKYFLISAPVDWAPDQLIRRFLLPTGDYVSCVLWNNLFHISGTDIVRCLSFRFQAFGRPVKNTKKFE
EGIFSDLRNLKAGTDASLEEPKSPFLDFLYKNNCIRTQKKQKVFYWYSVPHDRLFLDALERDLKREKMGQEATTVAVNEPALSFQFDSSQSLYEQLTKAQ
QANSSSFSAHASTAYHSASPMIRTTDSMPPPQLPPAMPIVPEETHDQGIYNSVGLTSATTTMGTGGIKAEQDFGQVQYDRNGVPVPRIHQRHTSMPTYME
YSPAPSFVSSHYDDYGHRGISFEPITPPQHNVQLGSEPAYIANEDTGLYTAIPDIGTTAAFNAMMQLPPSNFAAPQFSTASRTFPSTNVYSVIEGSPTYK
QRRRRSSIPPGITNAIAAATGNGQTHSNPHAAHRPSDLHHSISVQDGDESNPESPPGLTHSYKAEETQKMHSHENSRLGTPLPTLDETQADDHLSLINPQ
PEMPMLTSGEALESSGSSNSRSNVPGPVRRARSATMMELGPYPQKSHSCPIPSCGRLFKRLEHLKRHVRTHTQERPYPCPYCNRAFSRSDNLAQHRRTHG
AQAGQQPSANSNEEDREQQEPEHEATDQEHTTPDTSYLPTTVNMASVSMPSMSIPNLPSMVAPQMISPQLLQQQI
>HOMEO_Ci0004 (CIMG_06089.2)
MSNESQQHIPKRTGLDVLSGAEFTEIQESMGNMNPSDSMSFDLNSIFPYSEASSTHSTEAILYDTDQSVHQHVFSNPTIGYEQPQEPESIGKLSNWSKDA
VEVLKKWLKQNCRHPYPTKQEKAELAEQTELTVTQVSTWFANARRRGRHASADRCLTSTSLSCPDKPRKVSDEQWPSFSPLDRWRNSPPEVEAASLEAIR
SAVARSGTSYLPTGDLQHQSPPEDNDARSLVSSKASGSAVSSSSRSSAYSFDSNHSHGSFGRFYLEKVPRRRHRRQTRPASTLPTPSTKRSVGKRPYQCT
FCTDTFRTKHDWTRHENTLHLSLETFTCAPFGSTYNDPSGGTRMCTFCDCPQPSESHIESHRFSLCQQKPVALRTFYRKDHLVQHLRLVHGVNNLIPSMD
TWKSQVTNINSRCGFCGETFTLWSERNDHIARHFRKGALMKDWRGCRGLDPSVALAVGNAMPPYLIGIECTGIEPFSASWLAAKDARGAEPTDQRTSTAS
KPTPFEYLTARLTGFVNGIQAAGGTVSDELLQKEARCIIGNGTLCGIRSTTVPWQ
>HOMEO_Ci0006 (CIMG_08636.2)
MGTCEASPVDLLGASTCRVESASWSLREQSPIYSNSRHYSKVENTQQRQAYAWPAPALGRDCTGSNERRNGEQSPCHDEIQSDDGPQTMGGGILEEKTSK
RKMKRFRLTHSQTRYLMSEFTRQAHPDAAHRERLSREIPGLSPRQVQVWFQNRRAKLKRLSTDDRERILKSRAVPEDFDMAKALRWPYTNYSNTPASAAT
HCDNSLGRNDVPLVIESIKLSGEEYVTAPSSSPPTYGYYASGPLSVAEDNTSPDNIISNSSANERKLPVISWTYPQMPTPPSESATVPESTNPSEVRSPL

NRKWSGVSLGGISARLSPSKLSPHTMNRSLNTPITPSHSYYEKQLATPVSVGGFHERSASQKEVQSPMASTMPTTPLSVSSEEQVLRPNSYFGLSSFEIS
CPQGASPTMGYTASGVAFEPSPRFVEIPYSTNPVKDMWDMGS
>HOMEO_Ci0007 (CIMG_08965.2)
MDYIHTPFPFGEHNVGPFIDPSVGYGVPASFTQCNPPTDPCFVSYSPSEFPGFPGFYGHPVPFEDYHEYVENLSRPRLTKEQVETLEAQFQAQPKPTSNV
KRQLAMQTNLTLPRVANWFQNRRAKEKQQKRQEEFKRMQAMKSSENLKREEISSSTDARRTQYADKVSGSDHMLGENQVVSTPNDGNLNAEPLGNQSNSS
HPPVKEGVSLPPSRKLDPRGPHETSTTKTVDVAEGPQSREKNALPEKTNDGLTADQLESSAWSISSQLNNALCTEPIDAPNVNSSQPPTEFRSAELWQQE
SDAGNKPIFCVSYSTCEAHSGLSKGRSYPNDEPVMQTFPHTAILDFGATDIRVQRQESCPSCPQKYSSQRNLVQSMSIAERSIEKELQDPQVSPPDGTIF
PRRRDKKLDLAARRKRPRPAAIGIGGPSQALVGPSSMSPTTRTPTWAVSHSLRHVKSSHNLSSSLSPRYGGIRKVSAPLRSPLGMRASLDISGASSSNSD
FMVPPKPSKYVGAGNSYAQTHSYIPHYPTKPLKFMEHVQQLGKIGESSSRQLRNVRFKDLTHHGHNMALPVLHQNSEFMIKEFKHQQHTDMLASHQLASG
QPKINQSLGEFGDKNYCSKSTVPCVCF
>**HOMEO_Ci0008 (CIMG_09071.2)**
MSLKPESQSISAPSGAPAPPVAPAMANAPTNSGAPGPATSVQQQQQQQQQRTSRRPPRKSTLTQQQKNHKRQRATQDQLVTLEMEFNKNPTPTAAVRERI
AEEINMTERSVQIWFQNRRAKIKMIAKRGIETGEDCDSVPESMRQYLALHWDPSKSRNLFNRGYGSGDPMLNAPTSSSKIVISHFSCRSLRVGTWRRVGQ
NTMDLVIFYSPDKSCMTYYINNDSAGYKIEYPFSYIKNIALEPAEPGPTGDATPPKFGGIVIELNRPPNFYMDSSNSGGFYQCGDFTEDQQATKSLVHHL
GGGHPKVLSVQLAKLVSLESFQNRHIHYGFTGFASAPVSPHIIHRPASQPNQMARRQSAIYQENHFGMGLHPPRGHKRQRSRSVPVPVDFSAMQTPMPSF
HVQNPSTQFQPNPNIFAPVPQTHIPVNGVAGALQVDTSAGYNLDFHNYPMSAATASPPEFASPSLFATTAPPDQPQTTHLGAPYNVPFLSPSSMVDHGNV
IAHSASPLSHVSQAEPSIADQSPPLTTTQRSSPAEMFPVSNDPASSLPDDGLLLSEMYSKQNLNVSMPSPPMDDTTFALTLQGLHDQGASDNGVDFHGML
SFDTVDPMSLGADS
>**HOMEO_Ci0009 (CIMG_09634.2)**
MSDTDYTHTGNEPSSSNDADRSSPNTGSFLVHSNKSFTHGQPPKVDNKILGRQRRRRTSPEDYAILEAEYQRNPKPDKVTRANIVSRVSLGDKEVQIWFQ
NRRQNDRRRSKPLPPHDLGSSSTTTTDSQKDRSDNDVDSRPSPSQLSFSSQDQKKSMRPETYSPEVSVEHPDNECIEESGMAHATSSQTTVASTQTEELE
TTSCPDEAVGSFENRADKLDPVSKDVQQRSGCKRKWDEYDVKEVVEAKHARRSLPSQLATPPSLRISLSFDGEAMVRMEGEKTPSPPKPRDSLRISFSAD
GEAVVRSANEPSPSKSACTNARQARFGHLRRSTSAISFPTMRSRIEGKDIKPFGRSRNARTWELYCDDDARTALPNALNTRVDSTSVDNLSRCGPQRENS
KPLGVRSHIPNEIQEPQTPAKKRKKLTRAVSSLARLETGAKAPTSSGAKARKFAHDMKDHTGDSDKENWIPGTQISSVRRRGVQKQTTQARGRRVLGRSS
KQLEASQMGVSRLRRTKSARSAMISEKNENGSAVAGESEETSAFVKEAGSNPEEDLDCVQGLLSLSQGAWR
>**HOMEO_Pb0001 (PABG_02813)**
MAVPMLACLSWFCPGLLTYYQEKGRPIPRIINPRFAEAWQAEPQGQQLAPLRLSVGLDVKHVGESNTVLSDEVIMKLHTAKQVENLNCANASHGPLSLTS
KLAGLLHRAKYHLSDSRVFAIRVEVSERPDSSVEYTTATLRESSLTPIPLSLFFSACPLQILPPATPSALHIQNAPTKHQGKRSAPAAPGRTMEFFDFEQ
AAHEDYVAYDCPAIDESDIVAQYDSLVFYKDLGLPTGASAVQTDEAKGKNEPFISLFDKGVFPVTRATEPCDLCRARGLDCFMAKRGVMRNNGCTCCISL
YRECSFTRTKPHEKGLDTLHVVSEDSYVPVGGFTGKKALRSFKGGAGGSAFEDTDSKRRKTGARFSREALRALKTWLTENAGHPYPTDEEKDELKTKTGL
KRSQICNWLANARRRGKVRPSLRASSPRSTGPIDIPRKQLAPGVDIADLTPLERWKHSPPEHEPASATAIIRAMANAPYIPERNSSSGSRVRSHSRNTGS
SNDGSSFSNLMPAPSVSSYSFETTKSSTSDMSFASAFSHRSSRSSFNSADSKERRRRRHRSVVGQNTFQKARAARIFQCTFCADSFTTKYDWQRHEKSLH
LALDKWTCTPQGGAISLNGAIVCAFCKGTNPDIDHLEAHNFIACQEKSIHERTFYRKDHLTQHLRLMHSVKFSSWMENWKSATTEIKSRCGFCSSTFTTW
KDRVEHLSAHFKAGTDMSQWKGDWGFEPFVQRLVENSIPPFLIAQERNTLNPWVAKPAATKCSHVAERIASYPASISDSSNIPIPEDANCFRRLQIQLAA
YISREVARGIIPTDSELQNEARKVVYGCDDPWNQTCADNPIWLSILKRDSGLCDSPEVENIHLDDLGMQPPFAAPGLSQPPRHKLMAISSNLSNSGFDSS
GIYSPAFDSNGFSSGAPSVHGSFAGSATGSVGVSSTRIDGGWPCLTTSYTISSSAPVTVRFNPTAHIGTDSQYRQYPSDSVDGDLGANFDDMHIDTFEPG
GTTITAKVGAMNSSGHLSFKMPSSVAIDVPAPALSVSGTHIVSGSYEHCFRNIFDDHFDMN
>HOMEO_Pb0002 (PABG_03234)
MFPVSIYTPFAGWYNQQLESRESVTRRFEQFSTVIKGCDCLGADIVGQGRICLKLKVPLDSSPIFEIKEDPAIDSKGDNEIETHNEMRGAEPEVCLTETE
DMDANSEIYSAMPLVSKRLNSGIPLASNSGSAKFEAYDDIDEVGADWDNEIDVELDRNQLWPHENHEERQLQAEEDNHLKTTAELLAEKRKMKRFRLTHN
QTRFLMNEFTRQAHPDAAHRERLSKVIPGLSPRQVQVWFQNRRAKLKRLTSDDRERILKSRTLPDDFNIVQALHFSFVNKDQPSSSTASPSQTFKFCQGA
DTYSPLIIDATRRTREEEYVAAPLNTTSVYGGYFSPSTLSPALLGPEPELFPSTTETNINPICTSISNPRTPASRCMNMLIRSNNFSNSYSRISHTHVPY
LPLHETEARANAGLLGSPLRATISYAEATLDYGAPDASYTELDIHYGDQSYDSFMSQRSEESSSSKSLTGSQLGKREFSKALSARLRTAPAWFPAKIQLK
TEIDKGNGP
>HOMEO_Pb0003 (PABG_03964)
MSYLPHPFPFGGPPAIPVEQTVGLEVLIGHPHLGHYVNSFILPHDPFDIVDYCPSKPASIEEYEYTENLSRPRLTKEQVDTLEAQFQTHPKPNSNVKRQL
ATQTNLSLPRVANWFQNRRAKAKQQKRQEEFERTQASENGEQWESNETSRQEGGLEAQPERPELISTPTQDQLPISSSASPPSQPHTSTTQSECSVSVEK
AVNENMSANAQFYKSEHNAGANKALESNMMHHSSQPRLKNEAVGTATSGIPEWNGHEDDCNSVFLASPQSGGNSFDFTHVIRQQLTPNDNPFEIRIHESP
QFPAFHFNHEPDGWGCHGVPNLMGRQHDAQDLGDDFQPLPFHGLQSPLFPGEQRRGSYSSEHSDLTDIPFHPEINNDLEASPENISQLSLAQLHHHQVEP
CANWRYPEKEVDLAARRKRPRLAAIGTSAISRLYVPSSMSPTAMIQGMGAGHILRHAKSTQNLSPSRNSRYPGIRNTAAARRSPLGITSLAEANRFNPTN
ATDMMSAVPGLVTTALAPPTPLTPEDFQTLLPPTPSDNQYCVSPTDEMGCTRFFPISQPMQVQYQQMGISMPAAASQRTTFQEYTLSVPTNPMSTSLWHG
PSSMSSPETFQMQQPTIHMPQPTHISPIAYGNSLDSGNPSFVEGMASQSASPQCTIKSCSTPPSCPEGSTSGPHKVTEFLIQEFPQQQEAHRRAAQQLPP
QRPMNYTFSNHTPNDFYA
>**HOMEO_Pb0004 (PABG_04053)**
MTSNPLSSANAPITSSVHQNRVFRRPPRKSTLTQQQKNQKRQRATQDQLATLEMEFNKNPTPTAAVRERIAEEINMTERSVQIWFQNRRAKIKMIAKKGI
ESGEDCDAIPESMRQYLALHFDPSKSDARNLFNRGYGTEMHQSASSGKTVIHHLECGSLRIGSWCRVAQTAMDLIIFYCPAKGIVTYYIHNDSAGYKIEY
PFAFIKNIVLESGDTVPGPDGTGPRTRGLVIELNRPPNFYMDLQNNGGFYQCRDFTENQQATKSLVHYLGGAPKTLSIQLANLVSLESFQNRLVQYDFNG
YAVSAPVSPHLLHRPASQPNQLVRPTSSMYHQENHNSNNHFGMNLHPGRAHGHKRQRSRSVPALMDFASMTPIPSFHIQHPSTQFQPDPNIFAPIPIPQS
RIPHNHIANNDLLIDTSASYAVDFRAFPQPPMSATTVATTSTTTTATTTTDTSSEFANPQFFPTNAPQAQNQQSQQHQQSQSQPQHLGTPYSLPFLSQPS
PTTGPMLDQQPGLLRPSTSPLSHPYVNVSQVEPMIANASPPLSTLHGTGMGADVDEEMFSLSLSAEHMGGFPDSVEGNVGSDGANVVRGDGDKESIMLSE
IYAKHKLNAHMDLGMGGGMGLNMGRMGMGVGMNLPMHGHDAGQHSPPMDDDSFVLAFQGLQDHEQHDDHHDIEIEIDENENESGHVRGHSPVHEGDYHGL
LPFENGNGNGTVDLGSLVAGVSAGEG
>**HOMEO_Pb0005 (PABG_04066)**
MSRKVKTEEESKNMPVSYLSEFRSGGQTQTLPPFRDLLPQHLHEEIDSTPFYSPTSYHLPGTSYVSSAFHPRSTRGANLSRSLSTTKIDSYDGYLLRTDR
FSKDQSQGGVAPLKRQASDTEILSATNPTSHFSSGGALPPIRNLQPLPANIGPFSRVNVDTKPPALSLEYHSNSNPHTHSFGSKAPGTYLPASRPFLVIP
TNTLSHSEPARISQPYESSRYGPYQQSFRPELDYSPVSAGSLNDRNYGAQGEPIDPKNKKRRGNLPKSVTDVLRAWFHEHLDHPYPSEEDKQMFISRTGL
TISQISNWFINARRRQLPALRNQVRASEPDRNGHRQSPLSDNEQTSSQSITSSKSTKPKSAAAIPPPPLVGVSKSAQTFERPGSLQYPTLVSRELDHPED
AKRANPRDQSPALDDL
>**HOMEO_Pb0007 (PABG_04981)**
MNNPEQSSPPEGAKSSTMPSGNATNNNYPFLVHSQKTLTQNLPPRVNNKALARQKRRRTSPDDQKILEAEYHRNPKPDRAKRAEIVNRVTLGEKEVQIWF
QNRRQNDRRRSTPLHPEDFRPSKCSTSDQDANNNDNTATSGGPSTQLSRPGQETANQLGNVPREWISPQSSFGSGTGSDCGGTLDSSQMRASQITNVSQR
GELNDQVVSPEEQPGVYKEGPVIPTNEPCHIPSANRKRSRSEPQTPNANSKSEHSPPTSSTFIPPSLRISLSFDGEAVVRKEGEITPSPQKPRDSIRISM
SADGEALIRGANEESPTKGHSSLLNFTRPALGGLRRSISALPLGSLRTTNDQRDPKPFGRSRDSRNWELHCDTDARTALLGSKNSLSSLASRTGKLARRK
SDLGQPRALMQRTNFPNTLPLSREPRGKRKKLPRAMSSVACLESGYKSLPSKTGDGSLEYHFGDSDKENWIPATQISASRRSPSSQRTRRGVLQNPRVNN
EKGSSQSVNGSNKMQTSYRGQHPNSTRGGEGGGIEELCRGPDDVSQLISGPNQDEDLDCIQGLLSLSQGAWK
>HOMEO_Pb0008 (PABG_07873) Ste like
MYSQHGASMAPPQKPETFMLSNEAQQSLPQDAQVALQQVDNLKYFLISAPVDWSPDQLIRRFLLPTGDYVSCVLWNNLFHISGTDIVRCLSFRFQAFGRP
VKNTKKFEEGIFSDLRNLKSGTDASLEEPKSAFLDFLYKNNCIRTQKKQKVFYWYSVPHDRLFLDALERDLKREKMGQEATTVAVNEPALSFEFDSSQSL
FEQLTKAQQANSSSFSAHANTAAAYATHSTSPMGGGRATDSMPPPQIPQPAMSVGPEDNGSNNQQGMYHNSVSMSNTQIMNNNTAMIKPEQELQFTYDRN
GLPISRIHTRHTSMPTFMEYSPAPSFVSSHYDDYPGAAGARGISFEPITPPQHPLHNLGPGLGAESAYIANEDTGLYTAIPDLGSNHSTSFTSAMMMQLP
PSNFTVTGAGPGAGAGGHFSTATRMFPSANVYSVIEGSPTYKQRRRRSSIPPAISNAIATAVTGNGQTQSHSHGQAHSYTHAHAVSRPSDLRRSMSSSVA

PVPVLESEESSSSHDSNSHSPPGLTNSYISSSTVNTKDLLHELSRTGTPLPSLEENVQDDEAGLVGVMGAHHHSNGHSQHGPQGQNEGDNAVGVGRQCDR
PGPVRRARSATMMELGPYPQKSHSCPIPSCGRLFKRLEHLKRHRRIHESQQQGGGQPSLLANFSDEDHENENENEQEQDDFSSPSSVDLNGNSNNNNNHS
QNQSQNPNSHNNTSNPNTSNGSGSGNGNGNGNGNGNYMVNLSNVTSMPMSMPSMSMSIPMPSSMVNPQVITPQLLQQHI
>**HOMEO_Pb0009 (PAAG_00038)**
MARLNTEDIYNFDETGFAIRFCATIKDQTLQEGWFDELSNDWRLDDWHSSTSYSGIGMAVILPQNLIRPPVIPTAEALIPESMPQLQAPPHCTKCHTAFV
NIPMLGWEDKPAAGRSIVSYPQTSAGSTNKYPYSFGSALTEYPSQIATSHWDGQTLMFFPWMKLVREMTQFKPTVKNPTRSVPNYSSHRHKENGVMYAHS
SDLGGKEVIETNDKEDDIIEKRCNGRYFDHHNSQDFPFQSTVSQNSRLGIFHNPLSRKRTLH
>**HOMEO_Pb0010 (PAAG_00406) Ste like**
MYSQHGASMAPPQKPETFMLSNEAQQSLPQDAQVALQQVDNLKYFLISAPVDWSPDQLIRRFLLPTGDYVSCVLWNNLFHISGTDIVRCLSFRFQAFGRP
VKNTKKFEEGIFSDLRNLKSGTDASLEEPKSAFLDFLYKNNCIRTQKKQKVFYWYSVPHDRLFLDALERDLKREKMGQEATTVAVNEPALSFEFDSSQSL
FEQLTKAQQANSSSFSAHANTAAAYATHSASPMGGGRATDSMPPPQIPQPAMSVGPEDNSSNNQQGMYHNSVSMSNTQIMNNNTTMIKPEQELQFTYDRN
GLPISRIHTRHTSMPTFMEYSPAPSFVSSHYDDYPGAAGARGISFEPITPPQHPLHNLGPGLGAESAYIANEDTGLYTAIPDLGSNHSTSFTSAMMMQLP
PSNFTVTGAGPGAGAGGHFSTATRMFPSANVYSVIEGSPTYKQRRRSSIPPAISNAIATAVAGNGQTQSHSHGQAHSYTHAHAVSRPSDLRRSMSSSVA
PVPVLESEESSSSHDSNSHSPPGLTNSYISSSTVNTKDLLHELSRTGTPLPSLEENVQDDEAGLVGVMNAHHHNNGHSQHGPQGQSEGDNAVGVGRQCDR
PGPVRRARSATMMELGPYPQKSHSCPIPSCGRLFKRLEHLKRHVRTHTQERPYPCPYCNRAFSRSDNLAQHRRIHESQQQGGGQPSLLANFSDEDHENEN
ENEQEQDDFSSPSSVDLNGNSNNNSNHSQNQSQNPNSHNNTSNPNASNGSGSGNGSGNGNGNGNGNGNGNYMVNLSNVTSMPMSMPSLSMSIPMPSSMVN
PQVITPQLLQQHI
>**HOMEO_Pb0011 (PAAG_02161)**
MEYFDFEQAAQEDYVAYDSPVIDESDIVAQYDSLVFYKDLGLPTGASAVQTDEAKGKNEPFISLFDKGVFPVTRATEPCGLCRARGLDCFMAKRGVMRNN
GCTCCISLYRECSFTRTKPHEKGLDTLHVVSEDSYVPIGGFTGKKALRSFKGGAGGSAFEDTDSKGRKTGARFSREALRVLKTWLTENAGHPYPTDEEKD
ELKIKTGLKRSQICNWLANARRRGKVRPSLRASSPRPTGPIDIPRKQLAPGVDITDLTPLERWKHSPPEHEPASATAIIRAMANAPYVPERNSSSGSRVR
SHSRNTGSSNDGSSFSNLMPAPSVSSYSFETTKSSTSDMSFASAFSHRSSRSSFNSADSKERRRRRHRSVVGQNTFQKARAARIFQCTFCADSFTTKYDW
QRHEKSLHLALDKWTCTPQGGAISLNGAIVCAFCKSTNPDIDHLEAHNFNACQEKSIHERTFYRKDHLTQHLRLMHSVKFSPWMENWKSATTKIKSCCGF
CSSTFTTWKDRVEHLSAHFKAGTDMSQWKGDWGFEPFVQRLVENSIPPFLIAQERNTLNPWVAKPAATKYSHVAERIASYPGSISDSSNIPIPEDANCFR
RLQIQLAAYISREVARGIIPTDSELQNEARKVVYGCDDPWNQTCADNPIWLSILKRDSGLCDSPEVDNIHLDDLGMQPPFAAPGLSQPPRHKLMAITSNL
SNSGFNSSGFYSLAFDSNVFSSGAPSVLGSFMGSATGSVGVSSTGIAGGWLGLTTSYTISSSAPVTVGFNPTAHIGTDSQYRQYPSDSVNGDLGANFDDM
HIDTFEPGGTTITAKVGAMNSRGHLSFKMPSSMPIDIPAPAISVSGTNIASGSNEPCFRNIFDDHFDMN
>**HOMEO_Pb0012 (PAAG_02895)**
MSYLSHPFPLGGPPAIPVEQTVGFEVLIGHPHLGHYVNSFILSHDPFDIVEYCPSKPASIEEYEYTENLSRPRLTKEQVDTLEAQFQTHPKPNSNVKRQL
ATQTNLTLPRVANWFQNRRAKAKQQRRQEEFERTQASENGEQGESNETSRQEGGLEAQPERPELTSTPTQDPLPSSSSASPSSTPHTSTTQSECAVSLEK
AMNENMSASAQFNQSEHNAGANKSLESMYHSSQPRLKNEAVGAATSGIPEWNGHEDNCNSFFLASPQSAGNSFDFTHVNRQQLTPNDNPFEIRIHESPQF
PAFHFNHEPDGWECHSVPNLMGRQHDAQDLGDDFQPFPFHGLQSPLFPGEQRRGSYSSEHSDLTDIPFHPEINNDLEVSPENSSQLSLAQLHHHQVEPCA
NWRYPEKEVDLAARRKRPRPAAIGTSAISRLYVPSSMSSTAMIQGMGAGHVLRHAKSTQNLSSSRNSRYPGIRKTAAARRSPLGITSFSEANRFNPTNAT
DMMSAVPGLVTTTLAPPTPLTPEDFQTLLPPTPNDNQYCVSPTDEMGCTRLFPMSQPMQVHLESPPATPLSLSVLSQLQYQQMGISTPAAAPQSTTFQEY
TLSVPTNPMSTSLWHGATSMSSPETFQMQPPTIHMPQPTHISPIAYGESLDSGNPSFVEGMASQSASPQCTIKSCSTPPSCSEGSISGPLKVTEFLIQEF
PQQQEAHRRAAQQLPPQRPMNYTFSNHTPNDFYA
>**HOMEO_Pb0014 (PAAG_05540)**
MDNTAALYKQETTETARCGEGQLCGTTHHEQGSRTIVAVKLDFAIYLYPWMQSCVAEIWGAFYLDQHILDLLQFMAEFPANSRFPSPACLSKAFDICLLH
HNIKLFPHMTKLRLRVQVSLQLKSLPKTSVLAYCPSLPDGRQNLEESVALQGIKQGQGCDGGLLTFSADLQVSSGVTTRALDYIEVVNFQSLLNPGKRDA
SHQMLRIDQAFEAGNVNLHHPQYRRTVGRCNALIHSLATAGPGSHYELLYRRCSTKNIKVSLAQILQLFENLFGHLNEVRFLILHDLDIDGAGQDDFGAA
SVRLESTKQCAFQARNSQPSHFISLVGETKRKSKAYLLSCKADEPLTPRFEATEIIRHILGIVKAMFGYCGLFEIIRPYEPNKSRPGHNAVRLTTSSTFQ
RNALGPWLPLKQLRRCLLAVAPSPNSAHGTPTSGPNEVCNVREAWKKEAPDERKWRVFVSIPPPILTNPHGKTMTAPLRWPEDQTTSLPPLRIHPVRAVA
HYKYHTNPMPLNRSSYLVQGLENCKCGVSMSQALYSFWLLSSLTHLNLGSGLKKLDGRKGSQTLVLRLYDQFFVSTLIPEKCSALSPNRKKGPSGFQPSL
WSALHEKRILTINLRPYRLPLHPTLKLLSEHNLQTFVIVRRNRKILEVNYIHLTAQLQKFTTLSIEEFSAGARSLGEYVSMWGISPQHVIAGSYNKLGVI
TSRIGRFGK
>**HOMEO_Pb0015 (PAAG_08226)**
MASSTTEPVPNSTATTISNSNSTSMTPNNTTTMRSNPLSSTNASITSSVHQNRVFRRPPRKSTLTQQQKNQKRQRATQDQLATLEMEFNKNPTPTAAVRE
RIAEEINMTERSVQIWFQNRRAKIKMIAKKGIESGEDCDAIPESMRQYLALHFDPSKSDARNLFNRGYGTEMHQSASSGKTVIHHLECGSLRIGSWCRVA
QTAMDLIIFYCPAKGIVTYYIHNDSAGYKIEYPFAFIKNIVLESGDPVPGPDGTGPRTRGLVIELNRPPNFYMDLQNNGGFYQCRDFTENQQATKSLVHY
LGGPPKTLSIQLANLVSLESFQNRLVQYDFNGYAVSAPVSPHLLHRPASQPNQLVRPTSSMYHQENHNRNNHFGMNLHPGHAHGHKRQRSRSVPALMDFA
SMTPIPSFHIQHPSTQFQPDPNIFAPIPIPQSRIPHNHIANNDLLIDTSASYAVDFRAFPQPPMSATTVATTSTTTTATTTTDTSSEFANPQFFPTNAPQ
AQNQQSQQHQQSQSQPQHLGTPYSLPFLSQPSPTTGPMLEQQPGLLRPSGSPLSHPYVNVSQVEPMIANASPPLSTLHGAGMGADVDEEMFSLSLSAEHM
GGFPDSVEGTGGSDGASVVRGDGDKESIMLSEIYAKHKLNAHMDLGMGMNLNMGMGGGMGLNMGRMGMGVGMNLPMHGHDAGQHSPPMDDDSFVLAFQGL
QDHEQHDDHHDIEIDIDENENGSGHGRGHSPVHEGDYHGLLPFENGNGNGTVDLGSLVAGVSAGEG
>**HOMEO_Pb0016 (PAAG_08242)**
MSRKVKTEEESKNMPVSYLSEFRSGGQTQTLPPFRDLLPQHLHEEIDSTPFYSPTSYHLPGTSYVSSAFHPRSTRGANLSRSLSTTKIDSYDGYLLRTDR
FSKDQSQGGVAPLKRQASDTEILSATNPTSHFNSGGALPPIRNLQPLPANVGPFSRVNVDTKPPALSPEYHSNSNQHTHSFGSKPPGTYLPASRPFLVIP
TNTLSHSEPARISQPYESSRYGPYQQSFRPELDYSPVSAGSLHDRSYGAQGEPIDPKNKKRRGNLPKSVTDVLRAWFHEHLDHPYPSEEDKQMFISRTGL
TISQFSVMQAGFLTDENRAKPVVGKYLKLGKYDSPRFGSAAGLCKCPIWPTPDLSNTSYANFLRKTILPCKLFSVNLQSKISAAAIFPPLVGVSKSAQTF
ERPGSLRYPTLVSSELDHLEEDKKGQIPGTNHQL
>**HOMEO_Pb0017 (PADG_00736)**
MEFFDFEQAAHEDYVAYDCPVIDESDIVAQYDSLVFYKDLGLPTGASAVQTDEAKGKNEPFISLFDKGVFPVTRATEPCDLCRARGLDCFMAKRGVMRNN
GCTCCISLYRECSFTRTKPHEKGLDTLHVVSEDSYVPVGGFTGKKALRSFKGGAGGSAFEDTDSKRRKTGARFSREALRALKTWLTENAGHPYPTDEEKD
ELKTRTGLKRSQICNWLANARRRGKVRPSLRASSPRPTGPIDIPRKQLAPGVDIADLTPLERWKHSPPEHEPASATAIIRAMANAPYIPERNSSSGSRVR
SHSRNTGSSNDGSSFSNLMPAPSYDWQRHEKSLHLALDKWTCTPQGGAISLNGAIVCAFCKGTNPDIDHLEAHNFFACQEKSIHERTFYRKDHLTQHLRL
MHSVKFSSWMENWKSATTEIKSRCGFCSSTFTTWKDRVEHLSAHFKAGTDMSQWKGDWGFEPFVQRLVENSIPPFLIAQERNTLNPWVAKPAATKCSHVA
ERIASYPASISDSSNIPIPEDANCFRRLQIQLAAYISREVARGIIPTDSELQNEARKVVYGCDDPWNQTCADNPIWLSILKRDSGLCDSPEVENIHLDDL
GMQPPFAAPGLSQPPRHKLMAISSNPSNSGFDSSGIYSPAFDSNGFSSGAPSVHGSFAGSATGSYRQYPSDSVDGDLGANFDDMHIDTFEPGGTTITAKV
GATNSSGHLSFKMPSSVAIDVPAPALSVSGTHIVSGSYEHCFRNIFDDHFDMN
>**HOMEO_Pb0018 (PADG_01921)**
MSYLPHPFPFGGPPAIPVEQTVGLEVLIGHPHLGHYVNSFILPHDPFDIVDYCPSKPASIEEYEYTENLSRPRLTKEQVDTLEAQFQTHPKPNSNVKRQL
ATQTNLSLPRVANWFQNRRAKAKQQKRQEEFERTQASENGEQWESNETSRQEGGLEAQPERPELISTPTQDQLPISSSASPPSQPHTSTTQSECSVSVEK
AVNENMSANAQFYKSEHNAGANKALESNMMHHSSQPRLKNEAVGTATSGIPEWNGHEDDCNSVFLASPQSGGNSFDFTHVIRQQLTPNDNPFEIRIHESP
QFPAFHFNHEPDGWGCHGVPNLMGRQHDAQDLGDDFQPLPFHGLQSPLFPGEQRRGSYSSEHSDLTDIPFHPEINNDLEASPENISQLSLAQLHHHQVEP
CANWRYPEKEVDLAARRKRPRLAAIGTSAISRLYVPSSMSPTAMIQGMGAGHILRHAKSTQNLSPSRNSRYPGIRNTAAARRSPLGITSFAEANRFNPTN
ATDMMSAVPGLVTTALAPPTPLTPEDFQTLLPPTPSDNQYCVSPTDEMGCTRFFPISQPMQVHLESPPATPLNLSVLSQVQYQQMGISMPAAASQRTTFQ
EYTLSVPTNPMSTSLWHGPSSMSSPETFQMQQPTIHMPQPTHISPIAYGNSLDSGNPSFVDGMASQSASPQCTIKSCSTPPSCPEGSTSGPHKVTEFLIQ
EFPQQQEAHRRAAQQLPPQRPMNYTFSNHTPNDFYA
>**HOMEO_Pb0019 (PADG_02002)**
MTSNPLSSANAPITSSVHQNRVFRRPPRKSTLTQQQKNQKRQRATQDQLATLEMEFNKNPTPTAAVRERIAEEINMTERSVQIWFQNRRAKIKMIAKKGI
ESGEDCDAIPESMRQYLALHFDPSKSDARNLFNRGYGTEMHQSASSGKTGRQSPVSQIKLIHHLECGSLRIGSWCRVAQTAMDLIIFYCPAKGIVTYYIH

NDSAGYKIEYPFAFIKNIVLESGDTVPGPDGTGPRTRGLVIELNRPPNFYMDLQNNGGFYQCRDFTENQQATKSLVHYLGGAPKTLSIQLANLVSLESFQ
NRLVQYDFNGYAVSAPVSPHLLHRPASQPNQLVRPTSSMYHQENHNSNNHFGMNLHPGRAHGHKRQRSRSVPALMDFASMTPIPSFHIQHPSTQFQPDPN
IFAPIPIPQSRIPHNHIANNDLLIDTSASYAVDFRAFPQPPMSATTVATTSTTTTATTTTDTSSEFANPQFFPTNAPQAQNQQSQQHQQSQSQPQHLGTP
YGLPFLSQPSPTTGPMLDQQPGLLRPSTSPLSHPYVNVSQVEPMIANASPPLSTLHGTGMGADVDEEMFSLSLSAEHMGGFPDSVEGNVGSDGANVVRGD
GDKESIMLSEIYAKHKLNAHMDLGMGGGMGLNMGRMGMGVGMNLPMHGHDAGQHSPPIDDDSFVLAFQGLQDHEQHDDHHDIEIDIDENENESGHVRGHS
PVHEGDYHGLLPFENGNGNGTVDLGSLVAGVSAGEG

>HOMEO_Pb0020 (PADG_02012)

MQTGYFTVCNPEPDSSSPEDDPRGIFSRYLGVHRVRGLRAAKERFHQNNPTQTLSNMPFTLTHLERGLSVFWDYLGLNWRRYLAHHSATQRQIPDPIDCQ
LNLKNNLTLTVSFDLAFDLDQPYQNIPGFEPVHCRSNIVRMIHNSEWLPLSKLLSNVRLASNANHHRHKLSDQQLRIRMSRKVKTEEESKNMPVSYLSEF
RSGGQTQTLPPFRDLLPQHLHEEIDSTPFYSPTSYHLPGTSYVSSAFHPRSTRGANLSRSLSTTKIDSYDGYLLRTDRFSKDQSQGGVAPLKRQASDTEI
LSATNPTSHFSSGGALPPIRNLQPLPANIGPFSRVNVDTKPPALSLEYHSNSNQHTHSFGSKAPGTYLPASRPFLVIPTNTLSHSEPARISQPYESSRYG
PYQQSFRPELDYSPVSAGSLNDRNYGAQGEPIDPKNKKRRGNLPKSVTDVLRAWFHEHLDHPYPSEEDKQMFISRTGLTISQISNWFINARRRQLPALRN
QVRASEPDRNGHRQSPLSDNEQTSSQSIPFYDAMSDLNLFYDSWLSSFFSFRTYFCQFSVMQAGFLIDENRARHSNVLGPYSSQRWFLGSDHPEDAKRAN
PRDQSPALDDL

>**HOMEO_Pb0021 (PADG_02268) Ste Like**

MAPPQKPETFMLSNEAQQSLPQDAQVALQQVDNLKYFLISAPVDWSPDQLIRRFLLPTGDYVSCVLWNNLFHISGTDIVRCLSFRFQAFGRPVKNTKKFE
EGIFSDLRNLKSGTDASLEEPKSAFLDFLYKNNCIRTQKKQKVFYWYSVPHDRLFLDALERDLKREKMGQEATTVAVNEPALSFEFDSSQSLFEQLTKAQ
QANSSSFSAHANTAAAYATHSTSPMGGGRATDSMPPPQIPQPAMSVGPEDNGSNNQQGMYHNSVSMSNTQIMNNNAAMIKPEQELQFTYDRNGLPISRIH
TRHTSMPTFMEYSPAPSFVSSHYDDYPGAAGARGISFEPITPPQHPLHNLGPGLGAESAYIANEDTGLYTAIPDLGSNHSTSFTSAMMMQLPPSNFTVTG
AGPGAGAGGHFSTATRMFPSANVYSVIEGSPTYKQRRRRSSIPPAISNAIATAVTGNGQTQSHSHGQAHSYTHAHAVSRPSDLRRSMSSSVAPVPVLESE
ESSSSHDSNSHSPPGLTNSYISSSTVNTKDLLHELSRTGTPLPSLEENVQDDEAGLVGVMGAHHHSDGHSQHGPQGQNEGDNAVGVGRQCDRPGPVRRAR
SATMMELGPYPQKSHSCPIPSCGRLFKRLEHLKRHVRTHTQERPYPCPYCNRAFSRSDNLAHNNNNNHSQNQSQNPNSHNNTSNPNTSNGSGSGNGNGNG
NGNGNYMVNLSNVTSMPMSMPSMSMSIPMPSMVNPQVITPQLLQQHI

>**HOMEO_Fo0020 (FOXG_00297)**

MADPTKSPSFAATPDKHAPPAPRFHDIAVTPQSLPPLADASTNSAVSATDSEKHPKGKRKRTTAKDKMVLEEAYSSNPKPDKQARLDIVQRVSLSEKEVQ
IWFQNRRQNDRRKSRPLSPGEVAALHHGIMHPSTFDPITHTTTPSKPERPFPVSERSISRFGDPISVPPRHFDRTPSMPRYHSDLVNSTPITLGHDSFRY
YSDATPNRIDPTLPHEARDVSHSLSSSISSNVGYLSNRWNAGATSFSTPAAFTRSGDDSFRFDTFPPSSCASDRVIATPLSQSQPKVHLSTSLDGKAELV
SNQGSPSRELPPRPSSTTPYLPEERQRGLQRGLQRSSSAVTLPPLSELTSSLPPSLVRGRSRDVHAWEKCADAENRDELTAQAEYESNGSAAAAINILRS
TSGILQPSNAKRNAPMTRPQRPHQAKKAKLDPTGSSISRLDADLDEREKADREHGKVKVSMLVSPTGGDSDKENLSPDEETSSPDPYHRRPLPPAPKHAG
DNPRRVGRALQEQKSPNLLANRANMAPTRPRSIVKEGLEIFQDRMKPVLSSREQDMVRGSISPSKKPDMDCVAGLLSLSQGAWR

>**HOMEO_Fo0021 (FOXG_01706)**

MSRTTETKPRLSKEEVEILEAEFQKNHKPNSTTKKALAESMRVDNSRINNWFQNRRAREKKEKNIREYAAKQRMDKDTTANESGVHSDDDHLSDRVVSSA
PFPAPRPLEIRSTDVSSPEHESENDASHSDFGAGSSPNLSSQPTPEPVSASLNASLPSYSQYPQLIVPNEDGEPTPSLSQQCFPRLSTSPIQEQYVYSSN
NLAVDQSSQSSMGLKPSPSMDIASRRNRRPPQLAINASRSYSASGPRTGLDMGRRADVGHSMRRVASATGVGRISKPCGGPRSPYFERNPEALMQLNRSP
NFQSAATIAPPTPNTPVVANQQGLCEATPASTVAYEEKYPMDLAIHDPTLRTPPTTPGVMDQLYSNESVYQVAVADEPLVTPGLAPYPNEFEVPGTSSQV
PNYVSQGCSSQPQTPSYGAPMGPTYFGFAGGNAEYNWSDDASLSAHSSPGQSQQSVNFMNMTPSSFTYSDK

>**HOMEO_Fo0022 (FOXG_02103)**

MYPQHSTMAPQKPETFMLSTEAQQALPHDAQVALQQVDNLKYFLISAPVDWQPDQYIRRFLLPTGEYVSCILWNNLFHISGTDIVRCLSFRFQAFGRPVK
NSKKFEEGIFSDLRNLKSGTDASLEEPKSAFLDFLYKNNCIRTQKKQKVFYWYSVPHDRLFLDALERDLKREKMGQEATTVAVSEPALSFQYDSSQSLYE
QLTKAQQANSSSFSAQQSAFSQSQSTSPVMRAMDSMPPPPTMMPQSMPPLAEGMDAMVPYGTMAVPHPMAQAVPVKREPDFTRVQYNHNGVPITQGHQRH
ASMPAYGLEYSPAPSFVSSQYEDYSNRGISFEPITPPQQALGISAEPAYIANEETGLYSAIPDHMASMNGLNGMVQLPPSNLAGPQFSRPYGTNNVYSVI
EGSPTYKQRRRRSSIPQSMSAITTPAATAQAASHTHTHRPSELRRSISASVGPVAEGDESADNSPPGLSYSTSTVSVNTQHHQNMSRHGTPLSTVEGSPA
AHSMGMHQQEFPHLAREEFSGDVNDQRRSVPAPNGAVRRARSATVMEVGPYPQKSHSCPIPTCGRLFKRLEHLKRHVRTHTQERPYICPHCSKAFSRSDN
LAQHKRTHGREDGGDGSLNLSGEEDEDFSGDDHLGSLEEASPHSDSAYVTGSLNAAAHGSTPPSSMAPTQSYNSLETLSMPMTMSQPAAINASGMM

>**HOMEO_Fo0023 (FOXG_03759)**

MDFSLDDILFTGGEDAALTLPYDQIDDPAPQSRSPSNSLAEGANIDTMLQTINPQEAFNLQNSEIPDFQIDWNADFQQAKEASETPADDLNQDFSFLNQP
ATDWGIPPPLPASNDPIKTPMTTIDEFFVKNGASRPPVPCTNCRRTRLQCLILQTTVANPNPTKSCSSCVALFRECSLAGQKKRKPSEFETSEPVIGRLH
GVSEHSILGVPATVDEGQSSQGLSMAALSGKRANTRSVRKTRVLRNWYLSNLDHPYPSEEEKVSLSQQSGLSRSQVVNWFANTRRRHRLSSTYSASPGRG
RQGFAPGSPMPHYLRKNMSPLDRWKNSPPDEEPASATAIQNALAAQSSGHSSLDSDAGASDGPGSSASNDSLWLSNLQEASSNSASSCYSYRSRDAAFFS
RSGSSSVVEGPSISRTASSRSRTKKFIAFQCTFCGQSFKKKYDWVRHERSIHLPGLDSWICALPVTPDQSFLVWRMSEDGPQCLFCGENSPSDEHIQAHE
FDTCAERPVSERKFTRKDHLWQHLHKFHGCRKWDGWKPDLSLLQHRQDIIRSKCGFCQVMMDSWGERTDHIAAHFRSGLTMEQWVGGSGIHDPGNMGTED
R

>**HOMEO_Fo0024 (FOXG_03765)**

MNWAFNRPKPKSTSPPHQHFFTLKSAPYGYIHPRIDAMRVDNVDMGDTNDSEHRAPPDATSSGEPFLDLDALAFDPGALESNAYEPTFENGLEGNEFEFN
FDDLWSADMAQLPLAADDTDFTQTHQLQDDTSLQHDGLQRPGADQLAFNFGDLMRNISESPSSTQSPNILTPPIPSPLPPKIGHRFTLDAIRSLKDWFAR
NTDNPYPNEEEKNMLELQTGLTRTQITNWLANARRRRTTTDSGTQTASGKSGKVPSEYTPTRAGTPIPRRRSEKGMHPLQRWVDSPPENEPAAVSAIAQT
ESDKNRKPKPKRDILK

>**HOMEO_Fo0026 (FOXG_05651)**

MAAGSETEDIFSDLAFLNVEQSGSLDDQIDHWLRAPPAEPIGQESLLSGNTESSELLENDMSLSGLAFDSLNPNILHLPSAPVDTGDYPTTATSSMGEGT
SQSSIQTGKLGARFTQDTVKILRKWFLTHGHHPFPDENEKKILQQQTNLSKSQIMNWFANARRRDKISRSRDDLPCPTKAMDMPRRPDTPAPRRATDSMN
PLERWVESPPENEPASAVDIARAMASSPPACMGPTLRLYQY

>HOMEO_Fo0027 (FOXG_06267)

MSNMDEMEQFVNWDKAIDAFPQPQDGNAFAGLTADNNESIDLVLENVSEDDFSFCALQHFSDNNFPLPDMPAPTMDFTTTAEDISSQFQWDTPPSPCINC
AMSGFSCKKIREGMYKDYCTTCVALKVDCSFAVAPESINTSAAPFDINPFLPSGPQISNTLQEDNQDDSCQSSRNASRNASVSDLGNLGEIFNNKAAPPP
KIGARFSRESVRILKNWLSTHNRHPYPSDEEKEMLQRQTGLNKVQITNWLANARRRGKVQPPRSTSPHYTSSWSGPMDIPQRRGTPALEAMNPLQRWEHS
PPENEPASVSAIARAVTASSSGMSSGLDSPFSLSYADDGSSRSICNQSSVSSLGTSHSSNGSHGSAYSHGSRNSWGSFGSAPFNSHGRRRRRRRASAKIG
KEKTSLSAPLKTFQCTFCTETFRTKHDWQRHEKSLHLSLERWVCSPHGAKAVNPDTGILSCVFCGEANPDDAHIESHNHSACQERTPAERTFYRKDHLNQ
HLRLVHNIKFQDWSMKSWKVATPEIRSRCGFCGIVMDTWTIRVDHLAEHFKTGYSMADWKGDWGFDNPVLEMVENSMPPYLIHYERTSPLPYVATHSPPE
SPRNAYELIKLEMAFFTRNYQDQHGRLPNDEEMMVEGCRIIFASELLSLQGIATRPSWLRDIIMSSDTLQQKARFGPLRGAAENRLASLKINGKDNLFEE
CPMEVQLHEFVKAKRLLGLTAMDDELQEEACRIVGRVEEVSTHPSEAIANWLIRLATSTTNWLAPFRRRAHLPRSEDVVDHICRSTDPTSIDSTIHSYSR
LERELKDYLILQRSMGIEPTDEDLQRQARIIIYEFDDGWNQTAADNASWLEGFKNRHPASSNSSPAFSLQPSVNSTVSGTATTDATLFSSDCPVLLGLGC
DDMFDMGSGPNCPGPYFLNDANCYRRLAKELKRWVAGTMSANNPNRHVPSDAELQHQARWILYDDDDPWNQTAADNAEWLQRFKRDAGILKTDGPGLPMS
DGWALESGGSGFAPPYACPKASLEPFPADAQVSMGQGAKSLPAAIANSYIEKLTSQAARPAEVFCSRELERGLISYVEDHVACKGSMPTDAMLQTRARRI
LDSQTTPADDIDLLSKFKDMVAKKVPQAVAAQDTIASAPAMPSNMELNLSDEDVNNILQDMNFEFDAQDFGVAMEGLQDTGGVSLDMAGFTD

>**HOMEO_Fo0028 (FOXG_07428)**

MPADSDVDLVLSHFDDDDFTSWGRQLLNDDHFQPALADNFTFGSSHDNSLPFDLPWPTIGEDTDLTSHDSILPASLPDVSLSEAAQQQPATFSSAAISHP
THGLHKNGLPPKIGTRFSKESLKTLKGWLSSHSDHPYPDEEEKEMLQHQTGLNKTQISNWLTNARRRRTIRRPRSTSPYVRNTWTGPIDVPRHPNTPAFE
NNTNLNPLERWVDSPPENEPASVTAIAQAVALNSETSLGHNSPGSFASTDDGSNPSLYNVSSASSAGTSSGASFDSMTRQRGRRRRRRPVPKRKDQLSSA
VPLKKFQCTFCTETFGTKYDWQRHEKSLHLSLERWMCAPNGPRILNPQNHQICCAFCGEVEPSDDHIESHNLLACMERQPEDRTFYRKDHLNQHLRLMHN
VKFLDWSMKSWKVTGPDIRSRCGFCGIVMSNWSVRVDHLAEHFRKGETMAAWRGDWGFEAPVLKMVENFIPPYLIDNDRRSPFPYIATAVAAETPESAYE
LIKLELDRFTANQQEPTVPEAQRRELNEVARRQSARAFSGGCSEVTPATVAGQVGAFFLTDANCYRRLFRELTRFVSSAMSPNNPNCHVPTDAEIQHQAR

DDPWNQTAADNAEWLLRFKRDVGILPSGPGLSLDTNAWNITHGGTGFAPPYAFPNKRMLHSTSSTPASVNIPIGMNESTMTTNLTTTSPGLEKVEIFIGN
NATPFETNQALLDGYIETFSSRYGRPATVFCSRELESGLVTFVEAEITRGAGFPSDEALKLRSREILGSDKTAADDPSLLDKFKEWVKPSCNQRQEQQSV
NISSTLPYDVDINLTSAEIDSILADASFDMSLR
>**HOMEO_Fo0029 (FOXG_08967)**
MNFNNPNLFTDVDLLDGFISDDLTPYMSDSAPPTDLSNEMANLAPTHPVPQNPFTPLSLHGYTQQATPAPPAKVGGRFTRESVRILKNWLATHQNHPYPR
EPERRMLQEETGLTKTQISNWLANARRRGKIPSSASSPRHGDTPAMDIPPRPGTPAVRNTSDMDPLQRWVDSPPEDEPAAVTAIARAVASVRPQSGSTSP
YTRLSRTPSVRSVGTSRSSSISSVNSHTSRSSLGLNELFSRGSSRRRRRAYKEERRSLAAPRKAFQCTFCTETFRAKHDWQRHENSLHLPLERWVCSPEG
PRGQKADSPEIRCVFCGHVDPDDAHIETHNYSACKNRPVQERTFNRKDHLNQHLKLVHNAKFAEWPMRQWKAPPPAIHSRCGFCNLVMETWIDRVHHLAD
HFKTGKTMADWKGDWGFEPHILELVENSIPPYFIETERNSPFPFEGSRALVETPRTAYELLKLELAYFMQNHFYKHARMPTGDEMQLEACRILFAAEPLS
HGDLVCPSWLRDLVFSNVQISQQAQFGPMRSNAESRLSSLEISGKNNIFEGCPFETQLQEFVQAKQLLGL
>HOMEO_Fo0030 (FOXG_08975)
MAHQADPTGDLFIDFADFQNAAEAETISPGILQTSTAPPKIGNRFSSESVKILRNWFAAHERHPYPTIQDVQDLQEQTSLNRQQVTNWFANARRRAKSKK
TNKDEKRSQNESPPSAEYISVYFLYRNVQDEIRLAAS
>HOMEO_Fo0031 (FOXG_09086)
MATEIEHPMALGNVPEVTTPNSTSSPSVPTPVSASMKKETPSPSASAANNASRRPPRKSTLTQQQKNQKRQRATQDQLTTLEMEFNNNPTPTATVRERIA
EEINMTESSRRAKIKLLAKKSLETGEDIDSIPESMRAYLAMQAMESGKGLGGSYLGRTGLLPFGHGNMMLGGDQGAVIHHLTCRSLSIGKWTRVGQNTMD
LIIFYSPDKCTMTYYMNNEQAGYKIEYPFSSIKNIFLENGEGDPTKLGGIVIELNRPPNFFMDSSPTTNGFFQCGDFTEDLQATQCLVHHLGGNPKVLSG
QLAKLVSLESFMNRHNPHPYNDPHVLSVSAPVSPTARPQVAAQLSNPHVGMYQEQWGIHQMHSGMRPGPGHKRQRSRSVPGPVDFAMFQNQPMPSFYIQP
PGEMPPPPQHNPHIFAPIPQPPGNNMAPNLRIDTQAGFGLDMRQYPMSATTASPAEFPPSPGFFPPGPEAPQSSYNTPYSNGFLSPMVNPDTGVPTSVSP
LPFNSPGEPSILEQSPPMSMMGRPGSADLYPMNDGSCAVSEDGASLNEMYSKHTINLPMHTTSPGFVQHQQADLDMDQLVQFDAVDPSSLSPEAMPQAHQ
GN
>**HOMEO_Fo0033 (FOXG_09421)**
MMRIGQLIKDHESQDTLFVVYYGGHARIDESRQSTWCAWSAIQTLLERSLSDVLILLDCCAGAASATFPSGASITETISASSWDAIAPDPGRYSFTNALI
EVLQEWRVRAFSAAMLHAEVLARLKHPRPITINGKYFEARSTPVHFMMTSNHKAPSIEMSRMSRGDNLPSPELLPIPAMVHETGRAADSTTPVSRNDYMF
TEPNEDTPHVMISLALEDDQRLDINAWEQWLGAFPAMAKYVKVQGVFKSHSTMLLVSMPVSIWDLLPEDHATSFVAFIRSNNLMTQKPRNQSVPTLVPAN
RYPVENDSASFISGVSGTTFVPTELTGLTGQMGAFRDPAYGRQTGTVVRSTLPPIQQLSPLHMPSQPGSPPQSLPQRSMRPMHSTTSLTTLQRQQSSSSL
GGSGNLTRQMIMNQQQALRRTTFGSDVPEPKKFSPHVEKRLEEYYQTEPLPNDGQKAFFASNLGVEPWHVEVWFHHRRERDAFSQRFAALRVEDSKAGAH
EGPRMILPANLSELLDISLPGQSLLLDLRSSTEFQRSHIRGAIHLRAPQSFLRPASLDMIERAFPDAQSRRTFSRWQQARCIVFYSRGLEYPWECPSADI
LLEKLWACGWHGRCFILKGHYREFSDSFGKHVYRAQDSDAEKGPPKEAPISEGMPSNERELAALFARLETEDQTLHLSSSPGHNEERTTALVEQEKALES
EFQTHFPALFKKAQDVHGVGRSDDETFVTKAQMVEYLDRGLTKIRDGQASQAEATVYEPGHSKLAADSYFDRTNVNRESDEYVEVPRGDEVASDGSPQMT
SKGKEIRAVSSPDEITRRGRGGGLLNKVFRRT
>HOMEO_Fo0034 (FOXG_09424)
MSMLATASPSPHPSSFGMPRPWETNRCPDYSLRPRTENDKVALPSIRQAFPELQLQTQPPHDLNTKPPSTGPPLGAPPLTAASPQYIHSPNSSKRRRLSM
EREVETERVRQVPRLCYSPDRASPRQISPHLPIQGGSQENWAAPTRTSPFLTNGSNSHSAPMEASERAESRPTLPSLPPPRSLERELPVGRGTAPSDGYR
PPQQSMSHSRTPISEPGVSPYRENGYGYPYHHPTRYQSLSTGSAHSYDRTPFTPGTYNTPYQDFVRFGDMSSASLSGDNKQRKRRGNLPKETTDKLRAWF
VAHLQHPYPTEDEKQDLMRQTGLQMNQISNWFINARRRQLPAMINNARAETDAMTGARGGDLKVLATTERGDFDHSKREPAGPLSDGEGATYDEELEALS
QRRPGTIGRGSV
>HOMEO_Fo0036 (FOXG_13545)
MEDSLPNRSGGSSTPPPDMKLKTPIRTVRVPQLQALPQGVPPPPTALETEDYYGLWHNTPRLIARSNYPDDQWPGLCNNWSQVEDYHPLKVLWSDPEGIL
QKELTGALEYTQWNCFETYRIGSALQENQVKLLIIVEAGSTSWYHAWIVTRMHIRQPPVMDLESSESDDCSPFARFAEPVSPRVSDEISQEEEEPGWCRS
TLSSLVHVAGILAASFGSELWIMFVTLGLGVGLGRFGSGLAADKFGVLGVFVPVLVATSLTLFTWISVRTVPSIETSTNDSTTVFPSEFPDGRDLLDQPG
FSDLGFCSFQSPFSDEINPQLLYPVSGIGDSLGPGDSPEVNSIPPFGPTETLAPIASNVRAEASTISNPPPKTGKRLSLNSVRILNKWLSNHTHHPYPSV
RDVEAIERQTGLTRQQILNWFANARRRKKFNPLETTDPSSAEASPRDIPLRRPPTPTVQQSPLERWENSPPEDEPSTMAAIARAVSGASGSIDQPHDLID
YERNSPLPFSTQQGAPYSSTSAFELLQLELDYFYANYTDINHSIPSDETLHFEACCIIFGAEMNYQHPSTATSWLRDLIMGTQDIVNKARMTPMKSAARS
RFSELKIHSKKDIFEDCKLETSLRQYVDMLQLLNLEIGDVELQKEACSIINHMPDASPMFSQLLIGLVYGSTSWLASFRERVGLPPSEVVSSNTQEGLVP
PLLSSGGLPAISSNDHEHWRPPSGSNSPPISLGQKIVSLNDGNMYRGLTRDLTRYVARTISPLNPTSHIPTDEELQYQARWIMYDSHDVWNQTPADNLDW
LTEFKRDSGFF
>**HOMEO_Mo0010 (MGG_00184.6)**
MNMFQGGGHQAGYFVPQYNAMGQAAPHQHQERHQAQQRQQHGYPSYPYTNEHLMVLAQMQHQRTMMGQVHDASLSPTSKQDQPKPRLSKDEVDLLEKEFQ
KNHKPTSARKREIAELLKVDLPRINNWFQNRRAKEKGIKKTQEFAARQAAEQAEQQSESGSINKDEQDGEGSDGESTDNDHTLAQPSTAPFPADNASINS
GLEEDSTVSTTNSHTPIEPNVMTKEDLVRDGDEYPSPQSLPFQSTDQASDFNFQHNFVAMSEDQGFSSDYSHSGLPLNGMPAQFTHDGFSVSDQLSFVGQ
QYTSAVDNDAIIAPTPSFPSQLLGRLEFQDMDPHQAAPSSFDLQGMQVESPTAMTTIPDDGSPNSMPMSPPTPSDLRFKSPPPPANLASRRNKGVPAQLN
ATALRSYSYGPKTGLDLSKRADCPSPIRRIASATGPMPGRIQKSLTLGTGSSAPRSPMYLERTKDALIRSFSNTRSPVPLQPINTSLSPMTPGEYYGMPA
ATQGTRENTVSSSASDDEQSFGIGANGVQPNASALFSTLKTPPGTPGFNNGFVSDQQPPANQVNTFDAAWNFNPQDEPLVTPGLGSFGSEEFAMAPPAPG
YIGGSSQPPTPSYPHSIGPTYGGSFPWGSAAMFNGSRVGMPGSNTEYNFPESFVPDTSDISPSLSTKASKQFQFTQNVTPRDYNTGVEK
>HOMEO_Mo0011 (MGG_01730.6)
MPIVPEEDELVMDANFDDFINWDKAEPTLANVSSSMDFMPTTADATLGQFNGLDLDPPFLNFSHLGADNNILLDNELLEPSLGQPNLDFRDHFTDPCLHC
RANGYDCKMICEGTHKNCCTSCIAFRVNCSHKAEGAFSGGTESATPETPESKADAIGRSTGSGSAKTATRLSKDSVKVLKSWFANHSKHPYPTEEEKESL
QRLTQLTKVQITNWLANYRRRNKFARPRSTSPSVRNLSLGGMDIPQRRATPAPMDRRLRPSHYENMNPLERWKNSPPENEPASVNDIARAVSSASTAVSS
GFETPRSFNLTDDSRSLGHGSSVSSLGTSRTSQSSAAGSFASAYSHGSRNSFGSFNSLDRGRRRRRRRALPNMSSQPTRSSLTQPTNMYQCTFCPQTFKA
KYDWQRHEKSLHISLESWVCAPKGAKAFNPETSRIECVFCSEPNPTQDHLETHNYSVCQEKGVSERTFYRKDHLRQHLRLVHNTKFMNWAMSKWEQKHDE
LESICGFCGLRMTTWTARVEHLAGHFKAGSSMAEWKGDWGFSPFVLDKVQNAIPPYMIHEERISPWPYVASHPMPETPIHAYELLKTELVYWTQTSREQN
STVTDDELVHEACRVIYGAEVLSHKGIASAPSWLRDVLMSNETIAQRARHSPIRSGADSHVSILKINGKDNIFEACPLEHKLLEYVNSRTLLGLTPTDGE
LQLECCKILGHFEETSSSPSDLVGNLLVRLVMGSKSWLADFRLRARLPRSEDMIDEDRRPTDGKSLDSSIHNFSRLERELAQHVRSQRAKGIEPDGPDLQ
RAARIIIYGNDDGWNQTAADSPEWLGAFGERMQEAGSRAVLKLDDPLHHLFDPDAELIALQNAFASQQSSKDSPTTAITTSESAPNVSSPVGRCARSVPF
FIHDLNCYKRLKKELARWSASVMSPNNPNQHVPSDAELQHQARWILYDDDDPWNVTAADNFEWLQRFKRDHKIISDDSGPGLPDSVGWNLHQGGTGFAPP
YAYPAVAIEGTGGCGSGEGTVEIPVSVDAKLFQADQRAVDSYLQGFKARYPRPPAVFCSRELEGGLTEFMESQQVTMGAPPSDEMLRAKAREILGTETTA
ADDSVLLERFKAVFQPNNAVINTSEAEVDFSLMSGMQLPLPEVGAAFAMQAAETTNNGFDGSMMYGFGIQGTMDLVQSPSQLFDM
>**HOMEO_Mo0012 (MGG_04853.6)**
MATEINMPMGPGPLGEAGSLKIDTGSNMEVSIPSPVSAAPGELNETPSPSTPANPNSASRRPPRKSTLTQQQKNQKRQRATQDQLTTLEQEFAKNPTPTA
TVRDRIAEEINMTERSVQIWFQNRRAKIKLMAKKSLETGEDIDSIPESMRTYLAMQAMESGKGFPGAFLGRGMMPYGHGNMLMAGEQGGPSKVVIHHLTC
RSLSIGKWTRVGQNTMDLIVFYSPDKCTMTYYINNEQAGYKIEYHFSCIKSICVENADDPTKVGGIVIELNRPPSFFMDQSPTSNGFFQCGDFTEDQQAS
QCLVHHLGGNPKVLSGQLAKLVSLESFMNRHNTMAYHPDPMAHGGMPVSAPVSPTNRPSSQPNFAQPHVGLFQESQWGISPAHHVMRGPGHKRQRSRSVP
IAVDFSMLQTPMPSFYIQHPGEAQPQPHSPNIYAPVPQQPHALSPAGPGLRIDTQAGFGLDMRQYPMSATTAPSPSEYNSPSFFSQAPENTPLPASNFNT
PYSSTFLSPMMNATNLNVPQSVSPISFSGGDPAIVDQSPPMSMLGRSASADIYHGGDSSAISDDGHSLNDMYSKHAITLPMHPPHSPAFVEPSQAELDMN
QLVQFDTVDPSSLSPESVHQGIGGQ
>HOMEO_Mo0013 (MGG_06285.6)
MDSHQHHLHHNHPQQSQTHQDEPYVTLRTRAPRGDGASMSSADSTYGSGAGVSTPSATHDTSRVNFHVKHLETFAKSLEDSAGRAFPNRGLSMQRYSKVQ
VLLLHWKSDDLFVLPELEDLEQCMREDYSFETDIFAIPSENSHLELMLKVGSLIKDHESTDTLFIVYYGGHARIDESRQSTWCATRHSGSPWLQWSAIQT
LLERSVSDTLILLDCCAGAASATFPNGKSITETISASSWDAIAPDPGRYSFTNALIEVLQQWKVRTFSAAMLHAEVLARLKHPRPITINGKHFEARSTPV
HFMMTSNHKAPSIELGRIVPENRRPPSPPDEPEATMPLPSAPISSGRGGSVEGAGTEPTEHRPHVMISLALEDNQQLDLDAWEQWLASFPALAKYVKVQG

VFKSHSTLLLLSLPVMIWDLLPEDHACNFVAFIRSNNMIKPSAETNDTRIADNVSISGTTQAEDMIPEDLRAGPLVGGRHLPYDGQRTLTAAPRDYIRYI
PSSLKGHGAQQPRDNTYEKISRNEIMNTSRSSKWTVYHPESVPEAPRFNSHIVARLEEYFNNDPEPSNAAIEYYASNLGAQTNDISLWFYHRHKKERDSY
SQSKRKLAVEPPPPREEAIRMILPGHLNRLLEIYPSQVLFLDLRPPTDFQRSHIHRAANLRAPVSFLEHSTIEMIEGAFSDETSHASFSKWYSAKCVVIY
DRVIEFSWECPTAEVLYDKFRSKGWTGRFFVLKGPYREFSASYEKYIVGAKTSTAANEYLESLEHTPPTTADQVVEYQDQYEEWLRLVENEDRVPSANLT
PAKQSERLRAMQQNQEALENEFQSQFPELYRQSVGKLPQQDPWPTDRKERIEASQGPLVAPLSRALDKMHEAGMSPPPAPPPPLADRKGSSYSDKLEGSS
GRSGGYPPDKPVGGGRGGYSGDKMDVEQMEDEDMDGGGPGDLPGGQAAVGVGGGSESSKKRHQQQQPFWKRLRSSRS

>**HOMEO_Mo0014 (MGG_07437.6)**

MLVSRQCESEQNQWPFGKPAPSTRMSTHYEPPLSTQPDWPGSYPSFLPPGDNIFGQSYEHVSSSTDNAQQSASQPRSTVTENNAVGASDKSPINSNLPGL
GDRRSFDPLGLRQKQPSPIQEQPEEKGDMYAHKSSQSNQQEHRGASGEHHGLRLNTDSLMSAPAGGQGQDLLTTRLGANEGHGVKEEEDDGLDDEDMLDE
GDEDGGDGSSQPQTAAERTAQRRKMKRFRLTHQQTRFLMSEFAKQPHPDAAHRERLSREIPGLSPRQVQVWFQNRRAKIKRLTADDRDRMIKMRAVPDDF
DNVQALHSPYGAVHTLGTPISSPVDFSGSSYAEHMMRPLMVDVRRGDAEDHLSPTGLSPAFGSIGFNASANMGNSDMLSPISSATGERYGYPGHLSSPLS
AAPRTSNPFNRQDAGMQMHNHHQRQGIRSLQPLQLRETMSRSRSDSLQSPLRSSMSWKGDSIDYSTYHGPQSPHLPGRHPSIYQSSSDSIGTPSTMAGYD
SSTYTGSTVHSPTHMTYPTFQSSSIQNSTQNRTNRIRSVSAAATMQPLSLDLRSSPYRPPGQGLAAQSPTAQTRTASTSHIGGTSSSYSASFPSAPLTAP
VDFSLPRTSSYRSQTEYSVPQMSAPIAPPNDFSQAFQSIGGGAGRTPIRDGSSGTTLHPIGGGAGSQAVGSVCSPPNRSPDYTTSSDAALKRKQSFGGGA
GAATSGSAPYGTAH

>**HOMEO_Mo0015 (MGG_11712.6)**

MDEPNSPGSSNQEASFTVSPDSSMQANGDEHHPRAEDRERHPKGRRKRTNAKDRAVLEDAYSKNSKPDKNARLDIVNRVSLNEKEVQIWFQNRRQNDRRR
SRPLSDQDLAAYRNGGIKIVSADGHTGISFSQHCEQAEHDAARSPAQTTSTTPTGSPDLGPWMPGRYHQFTMEVPVPAKTPSHHSVSRPPADASHHSASA
KSFSFSQSGSFVSNQRWSTGASFSTPSSFKRHTDGGDDSFRTDPFAPSSCSSAASAPMLPLPRTHSRSDSRSNSAVRLSMSLDGKATLVSPSPERASTTY
KYDPRVEPIPISIPSLKRSSLNRSQSVTLPPISVLTGSLPGPGPLEGASPLPPRLTRGRSRDVHAWEFAADNGSDRDDELTQQAKDEASGSATAAISLLR
TTSASAGSVRASSPVLQPNGSKRNASVRPPLGPNGSGGAMQPFKRARLGRAQSSVARLQSPVSQFSVGRLIDHADSPHRSAGEDREGDDASAVKKMDVSS
ILSINGNESDKENWSPEGKDVTTTAPAAADRIIPPVTFPSMSTSRKALPGGGLSNTRRNIFLMDGRRSPLSNTRAYTAPGGVRAARRGHKAGSSIEIFED
HEDDLRGEEEEMVTTEGRRAGGGGRRDDEIARFMSGEVSPSKKPDLDCITGLLSLSKGNWR

>**HOMEO_Mo0016 (MGG_12865.6)**

MSTLAMAAPSPHAPFKKEYFSNWDDRRQADYAPSRPRSDPERIALPSIRQAFPDLLRLRVIPQDGQTSTPSSTTSPISGPPGTLTPPEYVHSPTSQNKRR
RLSFGDDQEERVSQVPRLYTSQSQAYQRRDNRPLSPVTLSGGPRSSTSDSWAGSSRTSPFLPGTSTSTLRSPAAIEQNDPMDRQRPTLPSLPHLNFDRSP
MESQSHMHHHHQQHPHQQQQQQHSHHVRAMSGDEYMMEQHRGMVQQHPHSAVEPGYRQPAPSGYPYPYHHPSRSQSLSIGAMPPMDRMPFSPSGYSAYHG
DYMRMGDMGGMGFNGDNKQRKRRGNLPKETTDKLRAWFLAHLSHPYPTEDEKQELMRQTGLQMNQISNWFINARRRQLPAMINNARAESDALAQGRGLSD
GSKILLSTERSDYDSDKRGSPISDDGASHLYHDDMHRRAVGMKRGSV

>HOMEO_Mo0017 (MGG_12958.6) Ste Like

MYSHPHNAGVAAAPQKPETFMLSTEAQQALPHDAQVALQQVDNLKYFLISAPVDWQPDQYIRRFLLPTGEYVSCVLWNNLFHISGTDIVRCLSFRFQAFG
RPVKNSKKFEEGIFSDLRNLKSGTDASLEEPKSPFLDFLYKNNCIRTQKKQKVFYWYSVPHDRLFLDALERDLKREKMGQEATTMAVSEPALSFQYDSSQ
SLYEQLTKAQQANSSSFNAQQVSFPPSQSTSPVMRAMDSMPPPPQMMPQPMPQSMAPLADGLDAMVPYAAMGMAPGMPPQPAVKREPDFNRVQYNQNGVP
INQGHQRHASMPAYGLEYSPAPSFVSSHYDDYGNRGISFEPLTPPQQAMGMGAEPAYIANEETGLYTAIPDHMNGVNGLNGMIQLPPSNLAGPQFTRSYG
SNNVYSVIEGSPTYKQRRRRSSIPPGMSAIAAATAAATAAHRPSDLRRSVSASVGPVAEGDESLDNSPPGLIYSNQPMSMANHQREAMEQMSRHGTPLST
VEGSPGMHNVNLEQQQYPMPSEDMTSPMDDRSRPMAQGGPSVVRRARSATVMGSEVGPYPQKSHSCPIPTCGRLFKRLEHLKRHVRTHTQERPYICPYCS
KAFSRSDNLAQHKRTHDRADGGEGLILSGEDEEEYSGDDHLGSLEEASPTSEGGYVTSSLNSAMAHSNTSQHPGSNAVSPNPGPMSHAPTYNSMQTLMQP
MQMSQPQPINAGGMM

>**HOMEO_Pa0010 (Pa_1_12070)**

MDMDYMDPYRRYAVQMQGYHGIQQQTQPQDHQYPYWSQHMVAYYQQHQQRAVMMGQGGMHMSKQTEPKPRLAKDEVELLEREFNKNPKPNSSTKRELAEQ
MGVEVPRINNWFQNRRAKEKQMRKTAEFEAQQAREKEASEVKESGDQEQGTVTEFYGLSNQHQPLGLSTAKFGGSDDGTDSDDGASGPQPLIESAGASST
VTPGGDTPVSPGSDYVHVKYEHVKSPVHRSQDTSDLDQPSTAMSAFTTPQQEINFQQPTPFSFRQANPELLDGLSGHELHRVQSQDHIDTGETSHFGSFP
DRDYFASPPIPRFPSEMIPENLVSAETELLQRRVSEENLVKCEALSPTSLPESPLTLSDLRFKSPPPPADIAGRRKLRRPAPLGPSSLRGGAGPKAGIEA
PRRSETASPMRRISSATGGLGGRVQKSFMGPGGPRSPFAMERNKEALLQSLQDGQSPAMASLNSAMSPLSPGGHNQSAREGTVGISSSDEEAGFAYGSLG
AVGGFSMYKSEATMKTPPGTPGLQMGMQDAYFTGSMDHAWNFAPQDEPLPTPSLCSHGGSELEFSMAPQMPGYVASQPVTPSFPPQSMGPTYNGFFGPSL
AQTEYHFPDSYATEPSARSSPTNMPRSKQFQFAQNITPQDFSADKS

>**HOMEO_Pa0011 (Pa_1_2230)**

MDHQAPLPRASSPSPAPRAQSPAVSGDEQQPSSPTTANALPAIPQQSSPEYRPEFLRSVGENERHPKGKRKRTAAKDKAILEAAYNANPKPDKAARLDIV
KRVSLNEKEVQIWFQNRRQNDRRKSRPLSPQELAALRYGGMQILSSDPAPYNTAFSSDITNTSPLQSLSRPEQEPTSPTQPDRPVSQAGEEAEPVAEVPR
ETSKWEEPRDNAKELATPAPKPRPAHDQSSALSQSFSASVGYLSNRWNTGNSFTTPAPVTAGRDEPFSWVTPMMQPITVSPNPGYSLESFSSSCPPAFSA
GSILPPPSTQPSRFRISMSLEGKAEVVASTISPPRPIAAPPTPDMLQSLRSIRRPNLQRSHSASPIVTLPPISVLTSSLTSHSPLPPRLTRGRSRDVHAW
EFACDAENREDALTAQAKNESNGSAIAAISILRSTSSTGGSPFQQSSSAKRNATISKATPRPGAAKKAKLGRASSSVARMQSVLGLSEKANHDNQQVVSS
TSEKIKVHARHSPSGHDSDKENWSPDEDGNPRTPYYSHAQSIMGSATTGRRPLPSSATRSEKDYRKHPRRTPTNNKTAPNDRANTAPVKGRGQRRDKRAA
QDSVLEIFEDEEAENRSPASSRAALDDEVERFMRGNVSPSKKSDVDAVAGLLSLSQGNWR

>HOMEO_Pa0012 (Pa_2_4990)

MLVTRQSAPEQSSWSIGKFETAFHKSAESLRMSNSYDAVTQSDWQGQYSFLPPGDNNIFSQSYEHVTGSVDHADSQAQPRSVAAPSNALDIEAPKRESPP
LGHHRLDPLGLRQPKQPSPIPEQPGNQGEQYAQKATESAHEEPLASTETPGMSLGSNPLSSVSSTGQGPEVGPGQPGNESQTVIKEEDEEVLEDEEMIEG
DGDGDAQPQPQTAAERTAQRRKMKRFRLTHQQTRFLMSEFAKQPHPDAAHRERLSREIPGLSPRQVQVWFQNRRAKIKRLTADDRERMIKMRAVPEDFDN
LGALHSPYGAVHGLGAPMASPVDLGGSSYDHMMRPLMVDVRRSDGDEHLSPTGLSPAFGSIGFNPSNSLSSPDILSPLSPTSTDRYGYSSHMSSGPLSGG
PRTSNPFARQPSIDSSMQMHSHHSRQQIRPLQPLHLRDTMTRSRSDSLQSPLRSSMSWKGDSLDYTTYHGGNPSPQLGGRPHGLYQQDQVGGSSGSGLGG
YDSSSYSGSTVQSPTHMNYPNYQSSSLQQSQQRGSRLRAASASLPLGLDLRTQYRSSVGSGSMQSATHSPGPRTTSTSQLGGVSSSYTASFPSAPLTAPV
DFSLPRTPGYRSTGGDYSMPQMSAPIAPPNDFSQAFQASMSNSSSRTPIRDSFGGGHGPLGLGQQSQSSVDRNDDYSQDPLGMKRKRSFTAATSSANSGP
SVYGATS

>HOMEO_Pa0013 (Pa_2_6460)

MATDVVKQEEGLTLHTPQLSTSPCSTKSSLPASNATTPTKDMKMETKPGQSASRRPPRKSTLTQQQKNQKRQRATQDQLTTLEIEFNKNPTPTATVRERI
AEEINMTERSVQIWFQNRRAKIKMLAKKSLESGEELDTMIPESMRQYLAMQAMESGKSIPGFFGRPGPFPYGHQGMMGAEQGGQGKVLIHHLNCRSLTIG
RWTRVGQNAMDLIVFYSPDKCTMTYYINNEQAGYKIEYPFSYIKNIYLNNSDDHHAGITIELLEAPFFYMDSATTSTFIQVNDFTEDLQASRCMTHHLGG
NPKALSGQLAKLVSLESFMHRHTAPPPPPPPMFDQLHTLSMSAPVSPQARPSSQPNFAQPHVGMFQETQWGIAAQHHTMMMRGPGHKRQRSRSVPGPIDF
QTMQLLQNPPSFHITQPEGQPHTQNPHIFSPIPQQPNMLGPIGPNLRIDTRAGFGSLDMRQYPLSATTAPSPSEFSSPNYFASQAPEPNGLPAGSFTPYS
GTFSPMVNPASLGVPPPSISPLSFNHPDPAIVGESPPMSMPPMCDGSAISDDGSMNDMYPGNKHTMTLPLHPHSPFMEQSQSEIELNQYMDLKHYDVDPA
SLSPESVQAQ

>HOMEO_Pa0014 (Pa_3_4650)

MPVIPEMEEYVNWDMAALPTTGEGPDFTSSSANMATVPALGAVGATEHLQDLDLALENAEGDDFSFWALEHFENNISPTLDGTLDANTCIGLKGLDRTGP
FEEDLDLPDVPCTNCQLGGYQCKRIPEGQYKGYCTSCIALCSECSFAETTGAGRAVGFPSNPWPIMGDHPMGIPQEEAHAGAALSGSQPAAATENTTSEP
ARATSKTRTGVRFSREELKILKNWLSTHSRHPYPTEEEKEMLQKQTGLSKTQITNWLANTRRRNKNAVAQRSTSPGVRTWTKPIDIAGRRGAASFELMNP
LQRWQVSPPENEPASVTAIQRAISTSSTLQSGLSSPYSSVHFTDDGSGRSICDSAISSANTSHSSGSFASAYSYGSRGSLGSGGSSMHRGRRRRRRKAAA
PAAANMTNSTPLRQPLKTFQCTFCTETFRTKHDWQRHEKSLHLSLERWVCSPDGPVTFNAESDQMQCVFCGHANPDEAHIDSHNHSACQERALAERTFYR
KDHLRQHLKLVHDAAYRSQSMEGWKVTTPEIRSRCGFCGIVMDTWSFRTDHLAEHFKRGKSMADWKGDWGFEDKVLDMVENSIPPFLIHDERNSPDPFEA
SHPPFAAKNAYELIKAELRAYIDDRPQGQEAPSDGELLSVVRALLEKTRTISRPCVGSTGSWLQDLLLAAPSDRPTRPSYKSSLQQLKITGKGDIFELDP
LELELEVYVKARRLLGLTAMDRELQSEAVNIIRRMDESSSDPSSDIVQFFTRLIYASTSWLTCFRARAHLPRSEDVVDVPQRSKDPKKIDAGIHNPSRLE

FELAEYVREQRSLFGEGFVPSDDDLQKRARVIIFEYDDGWNQTAADDAVWLAAFKNRHVFGNGTNDSSAYVPLDLSHESMFPNPLQLQLDVSDPAGRTRI
PSISNSSSSRSGSGSGGGSGSGSISTPSPPAGFRANLLGDVNCYQRLARELKKYVASAMSPNNPNCHVPTDEELQHQARWIIYEEYA
>HOMEO_Pa0015 (Pa_7_1730)
MYSQQNAAMPAPQKPETFMLSSEAQQALPHDAQVALQQSAVKYFLISAPVDWQADQYIRRFLLPTGEYVSCVLWNNLFHISGTDIVRCLSFRFQAFGRPV
KNSKKFEEGIFSDLRNLKSGTDASLEEPKSPFLDFLYKNNCIRTQKKQKVFYWYSVPHDRLFLDALERDLKREKMGQEATTVAVSEPALSFQYDSSQSLY
EQLTKAQQANSSSFNNAQQVSFSQSQSTSPVMRAMDSMPPPQMIPQSMAPLADGMDAMVPYGAMTMAPSMAPQPMIKREPEYPRAQYNQNGVPIAQTHQR
HASMPAYGLEYSPAPSFVSSHFEDYSQRGISFEPLTPPQQALGIPNEPAYIANEETGLYSAIPDHMNVGLNGMMQLPPSNLAGPSFSRPYAANNSFSVIE
GSPTYKQRRRRSSIPPSLSATTAAIATATAAHRPSDLRRSVSASVGPVAEGDESADNSPPGLTYSNSAISLNSQHHREMLEMSRHGTPLSTVEGSPALNP
VNLQQDFSPLNGDELGPLNERPMMQGAPGVVRRARSATVMELGPYPQKSHSCPIPTCGRLFKRLEHLKRHVRTHTQERPYICPYCSKAFSRSDNLAQHKR
THDRGDGVDGNFSLSGEEEEQYSGEDQLESLDDASPTSENGYVAGSLDSALHNHQNQNGMPPSNGPNMHQAPQQYNSLQTLSMPMTISHPQAINAGGMM
>HOMEO_Pa0017 (Pa_7_8640)
MIPELSMQSRPSDANTRVPSSVASPVAGYPAGAITPPSYTHSPNQNKRRRLSVGEDRDERGSQIPRVYPSPQREYHGGRGMSPAIASRSLNETWARSPSR
RSPYASHRNLPSMRESAPIESSDRYMSRPTLPRLPTMNFDQRSATMPRIRGPSSEDDYPDNFRHIMGGHSSSNSGEGYPPHHRSSAYFGYHHPSRVQSLS
MSSVGPFDRTPFSAGGYGSGYPEYMRVGELGGMGMNGENKQRKRRGNLPKETTDKLRAWFVAHLHHPYPTEDEKQELMRQTGLQMNQISNWFINARRRQL
PVMLNNARAESDAMSSAGRNADGKLLTSTERGEYDMGLGKRDSPMSDGDGSAYEDDLESLKRRHAVSMSRGSV
>**HOMEO_Nc0011 (NCU00097.3)**
MATSFYHHPPHHRPQTMHGLNVSSSHNDPRSLPSPGSALPPIRSIIPDFDKLDMDYGRRQSQAQKDTIDGGNSQGYTTDRLPTTGSSPSINVFSQHSHGQ
QDSGARTYLDGPLPRLPPSNAFAQHNGYHQNSEPRRGYPQYGQHQPIYRQDSYPPHGYPQDSYYPPLEHRHGPFPQPGSQQNLYEQRARSERLHYAHPWG
GHSDSAVSPRGGDGGKNKRRGNLPKEVTEKLYAWLYGHLNHPYPTEDEKQKMMRETNMQMSKIIRQRLLLPLLIEQAKAETEAAKHYRPNSSPIARRTRP
VSVSHTRHYSRSGPGRGVPSRRSVSVEPSINHLLNCPESDADVRPPQYATPMSDRGVLDYSSRESSESYEMDSRYQSYGHGHGHGHHASI
>**HOMEO_Nc0012 (NCU00100.3)**
MSYSDSAYGSASESCNPATHDVSRVSYHVRHLETFAQALEDLEDLEKCFREDYRYDTDIFAIPTENSHLELMLKIGDMIKQHEDKKTLFIVYYGGHARID
DSRQSTWCATRNLNSPWLQWSAIQTLLERSPSDVLILLDCCAGAASATFSSGQSITETISASTWDAIAPNPGRYSFTNALIEVLQEWRHRTFSAAMLHAE
ILARLKHPRPVLINGKHFESRSTPVHFMMTSNHKAPSIEIGRLVPDSRRSLSPYQQQHVVTSPQQDDWPLSPRSTYPYLPGMSAPMIGEPNEDEPHVMIS
LALEGDQRLDFSAWEQWLAGFPAIAKSNNLCRELGTTKSPHENEVTARNEEDEQQAMDADTESLMSEMATNYPGDTHRIDVQAASTPVWPPEQIGDPTDQ
ARDLTREAIAHLEDCFRKDPQPSETIIDVLAFTLNVHPNKIKVWFDQRRQHEQTTLNLQGLHYRSSTDQTPATKEGSARMILPGHLNTILTIYPHRGAVL
LLDLRSSTSFEKSHISSAINLRCPVSFIQHASLEMIEDTFTDVSSRQSFNRWYQSRCVVLYDSRIEFDWECPIANALLTKFRGNGWKGQCFILKGHYREF
ADSFDKWITGKGKSTAEFSSGKLLESETSYYNDVAEEEEQRQRRDREYEGWLKEFDESGEGLGGRRITELGPVKREERARAVAQRQIELERELEARFPAL
WRKVVAIRGQCEGRAGDEGESLYSRHRGGPPTTIQTDEIGDGSVSPPQSPPPPPFSPIADMSVQASSSGQFPMYDKAQLVEPLVSGLQKMREASGLSLGL
MSQDPAFAMSSSGHSQYAGDRGLGSTSHAYAPEKSGSYDQGGSTDGCINNSDGVVDPVSESWQGFSGGMEMASGTVSPMPPEWQDDSVHVPTTHYEVSMN
ALTAYVVHSGYFTRACLGLYYAKFHLIYGHMSSW
>**HOMEO_Nc0013 (NCU00340.3) Ste Like**
MYSSQHANMAAASQKPETFMLSTEAQQALPHDAQVALQQVDNLKYFLISAPVDWQPDQYIRRFLLPTGEYVSCVLWNNLFHISGTDIVRCLSFRFQAFGR
PVKNSKKFEEGIFSDLRNLKAGSDASLEEPKSPFLDFLYKNNCIRTQKKQKVFFWYSVPHDRLFLDALERDLKREKMGQEATTVAVSEPALSFQYDSSQS
LFESLTKAQQANSSSFPAQQLAFQSQSTSPILRAADSMPPPQMMQPMPSQMMQPMGQNMGQQMSQTMSQSMAPLSDGLEAMVSYGAMTMGPAMTCPQPMV
KREPDYGRVQYTQNGVPITQVHQRHTSMPAYGLECSPAPSFVSSHFEDYSQRGLSFEPLTPPHQQMGVIGEPAYIANEETGLYTAIPDHLNAGALNGMMQ
LPPSNLAAPSYPRSYASSSISQDHARGYSPMAAYTVIEGSPTYKQRRRRSSIPPSVLSATTAIAAGTPGVYKPSDLRRSVSASVGPVAEGDESADNSPPG
LTYSNPAINMARQQHHEMLELSRHGTPLSTVEGSPALNPMNISMGQSYGPLNDDELAPMSERQMSQGHPGVIRRARSATVSELGPYPQKSHSCPIPSCGR
VFKRLEHLKRHVRTHTQERPYVCSYCQKAFSRSDNLAQHKRTHDRGDGSEGTSNLSGEEEEEYSSPTSEGGYVHASGAPSNGNSSTPQSTMYNNLQTLSM
PMTISEPQPINTGEMMRNELEF
>**HOMEO_Nc0014 (NCU03070.3)**
MTLTWKLQKVCSTSACIDVKTLHSCFGMLKARQDSLPHFFSSFGRITNLPLAHLRGVDELGTMDPELLKYYQQQYYPGMGFAGPQHTQHHGPQYQVWSPN
SYQWTVLQQQRAAMMVGPGSLHPSKQTEPKPRLAKDEVELLEREFAKNPKPNTSLKRELAEQMGVEVPRINNWFQNRRAKEKQMRKTAEFEAQQARERAE
SESKPSGDQDQGAVTEFYGLSNRHQPLGLSTASIGSEDGANDDQSDRQRPNLEQSDLALAGNASAAATQSNASPSGSNSDGFAPADYNTPHSSGPRGYGL
VSHATPTTVSNSYGSASNHMQYSQPAPYSYGLNGPPRPVDGLPIATTMPQHNSLLGEANQFGSYTERTMFMHPSDGPFTSQMGGQDGIGNGTADHPTVRM
SIEGAINSENMSPSSGTGSSPVIADLRLRSPPPPSDIASRRNLRRPAPLGPLSLRTDCVGNGPKTGIDVAPRRMDNARTIRRMSSATGSLTGRIQKSLAL
NSGGPRSPFPIDRKKEALLQGIQNVQSPPIMASLNSAMSPMTNGGSDDEQAYTFGSCGAVSGMSSMPPFKSEAGIKTPPGTPGLGVHFRDPFAYSAPADN
AWGYVSSDEPLPTPGLCSNGGSELDYSMAPQQQLSGYVTSQPVTPSFAAAIGPTYNNVYGTNGGSHLARNTEYTFPESYQPESSSRCSPAGQLRAKQQQF
QFAQNVTPQDFSHEK
>HOMEO_Nc0015 (NCU03266.3)
MDTLAPTPSTPSPQLSSAVPGLAHEVEHEELSQSTSQNQVFESPLLRESQTPAPQLSSQLSGEVERHPKGKRKRTTAKDKAILEAAYNANPKPDKAARQD
IVNRVSLNEKEVQIWFQNRRQNDRRKSRPLSPQEIAALRYGGMQILSSDNTLPVYTSEPENTSPIQVVSSLDQEYSSPREVASPSKLDDHETEQNHEDTL
HEDTLPLPEPQSQPQEEQQSLPHPVYATPAQKQRHAGDDDSLLMSQHLPSSVGYLSNRWNYPAHSHVLSTPISFDRGDDSFNNFPPSSCSSSTSALRPRL
STGLDGKAEVITSPPQNPASLPPSELQSFDSACRPSYQRGPSESPVTLPPISTITSSLPSSQNAAPLLPRLARGRSRDVHAWEFACDAENCEDALTVQAK
HESRGSAIAAISLLRSTTSSTAGGSPLQLSRSNKRNASISRMTPRADSSTKKPRLTKGLDATGRLETPNNNNTWAGKRQSFTADTPADFSHNTKPKSSSL
FISPQGNESDKENWSPDENGNPRFSYHPPSSAIRPTNRRLLPSGPLSSSIHDKATNPRRTPARHALIESSPSFSLSGNRTNTGPLDGGGRGQHYRGGDKR
GGGGSELRIYEDFVVATTEHNTPTASRRAPPFEDDGSDEVERFMRGEISPSKKGDADAVAGLLSLSQGNWR
>HOMEO_Nc0016 (NCU03593.3) Kal1
MATDIINQETLAPPSMKQEAPSPTSSMTPLPTPTSTSCPKDSVSPSPSTPANGNKNHNQSSASRRPPRKSTLTQQQKNQKRQRATQDQLTTLEMEFNKNP
TPTATVRERIAEEINMTERSVQIWFQNRRAKIKLLAKKSLESGEDMDSIPESMRQYLAIQAMESGKGLGYFGRTGLLPYGHQGMLGGEQGGQGKVLIHHL
TCKSLTIGKWMRVSQNSMDLIIFYSPDKCTMTYYINNEQEGYKIEYPFSYIKNIYLNNGDGDHMGGITIELLHPPLFFMDAAGQASGFFQVGDFTEDQQA
SSVLTHHLGGNPKILSGQLAKLVSLEAFMHRHSPNHMFQEMHHPMALSAPVSPARPSSQPNFAQPHVGMFQENQWGINVHQNNNMMMRGGPGHKRQRSRS
VPGPIDFQTLQMLQQPPSFHITQPEHQQAPVHNPQIFAPIPQQPNMLGPLNPNLRIDTQAGFGSVDMRQYPMSATTAPHSPAGFNSPNYFAPPDNGNMAG
SFTPYSATFSPMINPSSLGVPPPSISPLSFDHPAIMSESPPMSMPPICEGSAISDDGSTHDMYQPGKHSYTLPMHPHSPFAEPNQAEMELNQYMDLKHFD
VDPASLSPESIHHTQ
>**HOMEO_Nc0019 (NCU05257.3)**
MPTIHEMEAFINWDGIDPASMPGTRHPNDLDLALENAADDDFASWALQHYEQNNLLGLGETATAGDSIVAFEDSFDMPSSPCNHCQANGYQCKRIREGSY
KGYCTGCVALNRVCSLGLVDQPAAPPRNPGSTTSVEEQDRPSTPAPPIALAATKVNNRFSRESIKILKNWLSIHQKHPYPNDEEKEMLQKQTGLSKTQIT
GWLANARRRRGKVMGAPRSISPGVRTLSTNLDIPQRRPQLELLNPLQRWQVSPPEHEPASVTAIARAVTASTTTLSSGSPHSNNFNLTDDGSNRSLCAAS
SASSFNTSISSGLSFASAYSYGTHDSLGSYGSSMNRGRRRRRRKAAPVPSEKRHSLSAPLKTFQCTFCTETFRTKHDWQRHEKSLHLSLERWVCAPEGPR
ASNPENGQVSCVFCGLANPDEAHIETHNYSICQEKTQEERTFYRKDHLRQHLKLVHDVKFVNWSMEQWKATTPEIRSRCGFCGIVMDTWSIRVDHLAEHF
KAGQTMAEWKGDWGFDNPVLEMVENAIPPYLIHDDRNSPNPYTATQESSETARNAYELIKSELMYYLTKERDIMGRVPTDEELQVEACRIIYAAELQSNQ
SETAIPSWLRDLLLSSEPLALQARMGPIRSANESRQAVLRINGKSNIFEEDPMENELHEYVKARRLLGLTAMDSELQYEACNIIGRMEESSSHPSEDVAN
FLLRLIYASTSWLADFRQRAVLPRSEDVGDEARRSTDPSKIDSTIHNYSRLERELAEFLHLQRSMGIEPTDMDLQTKARIIIYECDDCWNQTAADNSDWL
AAFKQRHVSPEASAVALATCEPLTISSVSRKGFTPSMDMATWMGSSCFNGPRNTSLGSIGTPTFNIAAGVDGHIGKPHNAVKIGPYFFNDANCYRRLARE
LGAYVAAVMSPNSPDCHIPSDEELQRQARWILYQDDDPWNQTAADNAEWLRRFKRDVGLLTDAALPGLPECTQWSATQGGSGFEPPYLFPNPHAQATTVE
TDIPIQMKEAKRMFVAEKETANKFVRGFKTRWQRPAVVFCSRELEKGLIKFVTTCVRGGSDGGGGGGGFARGMFPSDEAIRAKAREIQKLSTTSADDVVL
LEKFKNMMRERLGLMSASSSSLGSTPDFPSSVGAAGMGLMQGQSSSSVDTSAVPSPVTAAGFTITGTTTTATTSPGMGMSMDLGLADMGNMSNMGVAMGM
TNLTDWSSSSTGMMMGLGTSMDASCTNMGMGMGMGMSMDMNLDMTNTSMSMMNMSTSMPATHMGMLAAGGGGGGGRGQDDHNMMMMMMFPGNEMDDLLQE
SNFGFSSNDDDLAVMGGIGQL
>**HOMEO_Nc0020 (NCU09556.3)**

MSTETPNISLGSHPLSSVSSAAHEPKLSTAQSVNEAQSTIKDEDDDVLDDEEMIDGDVEEEPQAQAQPQTAAERTAQRRKMKRFRERLSREIPGLSPRQV
QVWFQNRRAKIKRLTADERDRVMKMRAVPDDFDNVQALHAPYGAVHGLGTPLTSPVDFAASSYADHMMRPLMVDVRRSDTNDHLSSTGMSPAFGSIGFNP
SNSLNNPDTLSPMSPPSTDRYGYSNHLSASLSAGARSLNPFARQPGLDASVHMHNHHSQHQIRTLQPLQLRETTNRSRAETLQSPLRTSMSWKGESLDYT
TYHGSNTSPTMNSRPQNLYHQDHMNSTSTSNLGSYDPTHYAGSTAQSPTHLSYPNYQSSSLQNNSRRNSRLSASSASLPFGLDLRTQFRSAVGANSLQST
AHSPTSTRALSTAQLGSGGAPSNYSASSFPSAPLSAPADYSLSRTSPYRPPPSAGATDYTIPQMSAPIALPNDFSQAFQASSMSNGGGSSRDTQSLRDNF
GASVLGLGHGHGHNNPASHHRTTDDYSVNDPLGMKRKRRYSGTAPTGAGAQRIGAYGAAV
>**HOMEO_Cas0016 (orf19.3201)**
MNSEIESSLTLLKSVEKLVQATSVYKNEDNEEIFLQLKRERQENSNLHEETETLFHESLDRLMKLSSIAGSKVQYELSLNNLYNLLLRTRKQEEEEAFIF
PTVSTEEFALEQITFEVSDQMDDDKDSEDDILIKGEEIKKSKKKRQRLDNSTKEFLEKVFEKNKQPNRRERELIAEKHGVSLSQIRVWFTNKRMRKKEPK
LKAKSSSNST
>HOMEO_Cas0017 (orf19.4000)
MSPDSISSLVNQDLSTPTPPTSLSSTSTNSNTNASSGQKRIRATGEALEFLISEFETNPNPSPERRKFISDKAQMNEKAVRIWFQNRRAKQRKFERQMLR
KETDSPGNYAGIYNTYTPNPPTVTTDMTNFNVNGSAGGATADFNDKLKNISSIPVEVNEKYCFIDCRSLSVGSWQRIKTGFHQSNLLTNNLINLAPVTLN
QVMSNADLLIILLKKNLELNYFFLAISNNSRILFRIFYPLNAVVKCSLFDNNYYQNNNTNGTNSSDDNNISEIRLNLCQKPKFLVYFFNGSNTNNQWSIC
DDFSEGQQVSCAFAANDNSLPSNGKNQSFSNTIPHVLVGSTLSLQYLSQFILQHQQRQRQQQRQAQPQPQPQPHNQFDFNSQPFETIPTSAINNNFNTTK
TVNSSSMQGFVPGDFQDLPAIYESISNSNHTTTTDLKDTNATTTTTNKHTPSSTTFTPQNLNGSNQSQTNVTYTNNYNESPFSIASTTNNNNTNSYRSNS
QSHNPIFSDQLFYESSESASTNSPQFAMKKMNSETKLYINGNVSSNSSTNGPPLDDDNNLFDGVTRFTTTETSPEDDIIGMFTSQAHEPSAFELANGVTL
SGSSYTHINNGSLTKSDKTFTGLSETSNNNNNNTNGINFVDDFHVGNEFGDIDFEHHYSQDHHQQQQKNDNNNGNTNLDSFIDFEN
>HOMEO_Cas0018 (orf19.4433) Cph1
MSITKTYNGDPTSLVPTQLVKESLRLIEDLKFFLATAPANWQENQVIRRYYLNHDEGFVSCVYWNNLYFITGTDIVRCIVYKFEHFGRKIIDRKKFEEGI
FSDLRNLKCGADAILEPPRSEFLEFLFKNSCLRTQKKQKVFFWFNVPHDKLMADALERDLKKEKMGQRPTTMAHREPALSFHYDESSSLYTQLGKHMETQ
KRINDAATSSTSNTATTLTDTGVSSGLNNTTSGGGSDSATSTHNNNEASTKPSNGSEKSSPEYTTTARGRDEFGFLNEATPSQYKANSDYEDDFPLDYIN
QTTQNSEDYITLDANYQAGSYANMIEDNYDSFLDATLFIPPSLGVPTGTAATATTSNQVAFNDEYLIEQAQPIRTPLPPISSSTISGLLQPKSAAKFFSL
QSANGGEEFFPAYQNDPSTANAGFVPPISAKYATQFATRQVATPTYIKAIPQTGAAAATGNGGQPQQYYDQATGNAFYPAEIPVLYNVVHPESEYWTNNS
GAVATTAAATAPMYDASGFPIPINQSYMVMNEHEMVPYQYMNSNGAMIGMIPPHQQQQQQQQQIAMGYQSMLRQQQQQQQQQQQQQPSSTMTKKKKQIHS
FNNNKSLSSNGGGITKKSHDNNNHSKVKTLYGSLNDVVNSKVTKVINKEEVKQSQT
>**HOMEO_Cas0019 (orf19.6514)**
MKLDQLINQAAPQPVPTVTRLLSIGLSSSSSTGVTNSGNAPLLPHNHNQFNYYPKTATTSSVHNQRVNLPPISDILQQAQHYLPPPPQQQQQPQQPSQSV
SSYRQAQAQLPPLPLAATANSNSNYKYYDYYQSPNSDHQQRSYTSSSSTVPSPRIPHSDNTVPSYQQPMMVRNNSYPPIQQQQMQTQYHQQPQYLGVGGD
SYFRNRTPQPPVINHNHNNGSNSTLSSSSMSSPLSSPYDSQNHTSDFSATGTPTDNFMKHASGASATNGGVNNNRKTRNNLPKEITFILLQWLNDHLNHP
YPSSFEKNQLMISTGLNQQQLSNWFINARRRKIKLLKQQQRLNF
>HOMEO_Cas0020 (orf19.7017)
MMMHLQQTPTKRSILPPITPLTNSNNGSKLSLPPLSSILPTAPSSHYRDHPLTTSTPYKAPLPSINTSAAYQSPSMTYGSYIPTPESATSLVTSSNKRSF
SVLDDSSIMDTSILEERKLKASTSSSVASTSSSSPTLSSPASTPTDNKAYAFISHSLATFPLQEPSIDNAPLARRKRRRTSPHELNILNQEFALGTTPNK
SRRIQIAKKVSMTEKAVQIWFQNKRQSIRKQLNLEKEITVLPPTSNYPPFQQHQPEQISTLPPPPPAQVQPVQPVQSVQLPIQAQSTPLPKPASISPLPI
TNSYTSMKFKFMPSKSKVTRKLQECKALGDITNLQ
>HOMEO_Sc0024 (YCR039C) MATalpha1
MNKIPIKDLLNPQITDEFKSSILDINKKLFSICCNLPKLPESVTTEEEVELRDILGFLSRANKNRKISDEEKKLLQTTSQLTTTITVLLKEMRSIENDRS
NYQLTQKNKSADGLVFNVVTQDMINKSTKPYRGHRFTKENVRILESWFAKNIENPYLDTKGLENLMKNTSLSRIQIKNWVSNRRRKEKTITIAPELADLL
SGEPLAKKKE
>HOMEO_Sc0025 (YCR096C)
MRSIENDRSNYQLTQKNKSADGLVFNVVTQDMINKSTKPYRGHRFTKENVRILESWFAKNIENPYLDTKGLENLMKNTSLSRIQIKNWVSNRRRKEKTIT
IAPELADLLSGEPLAKKKE
>**HOMEO_Sc0026 (YCR097W)**
MDDICSMAENINRTLFNILGTEIDEINLNTNNLYNFIMESNLTKVEQHTLHKNISNNRLEIYHHIKKEKSPKGKSSISPQARAFLEQVFRRKQSLNSKEK
EEVAKKCGITPLQVRVWFINKRMRSK
>HOMEO_Sc0027 (YDL106C) Pho2
MMEEFSYDHDFNTHFATDLDYLQHDQQQQQQQHDQQHNQQQQPQPQPIQTQNLEHDHDQHTNDMSASSNASDSGPQRPKRTRAKGEALDVLKRKFEINP
TPSLVERKKISDLIGMPEKNVRIWFQNRRAKLRKKQHGSNKDTIPSSQSRDIANDYDRGSTDNNLVTTTSTSSIFHDEDLTFFDRIPLNSNNNYYFFDIC
SITVGSWNRMKSGALQRRNFQSIKELRNLSPIKINNIMSNATDLMVLISKKNSEINYFFSAMANNTKILFRIFFPLSSVTNCSLTLETDDDIINSNNTSD
KNNSNTNNDDDNDDNSNEDNDNSSEDKRNAKDNFGELKLTVTRSPTFAVYFLNNAPDEDPNLNNQWSICDDFSEGRQVNDAFVGGSNIPHTLKGLQKSLR
FMNSLILDYKSSNEILPTINTAIPTAAVPQQNIAPPFLNTNSSATDSNPNTNLEDSLFFDHDLLSSSITNTNNGQGSNNGRQASKDDTLNLLDTTVNSNN
NHNANNEENHLAQEHLSNDADIVANPNDHLLSLPTDSELPNTPDFLKNTNELTDEHRWI
>HOMEO_Sc0028 (YDR451C) YHP1
MESRNTVLPSLPNIITGTSNSPFQLHTLPNTNFPSDDQGDIRLPPLAASAHIVRPVVNIYKSPCDEERPKRKSPQAVDFLSQRVTTSMTPLSKPKKLSSH
SPFTPTVRVCSKEQPPQSMHSYKKVNILTPLSAAKAVLTPTTRKEKKRSFAFITHSQETFPKKEPKIDNARLARRKRRRTSSYELGILQTAFDECPTPNK
AKRIELSEQCNMSEKSVQIWFQNKRQAAKKHKNSGNTSHCKVHSNDSMSMISYSDAALEITSTPTSTKEAITAELLKTSPANTSSIFEDHHITPCKPGGQ
LKFHRKSVLVKRTLSNTGHSEIIKSPKGKENRLKFNAYERKPLGEVDLNSFKN
>**HOMEO_Sc0029 (YGL096W)**
MGTSIVNLNQKIELPPIQVLFESLNRENETKPHFEERRLYQPNPSFVPRTNIAVGSPVNPVPVSSPVFFIGPSPQRSIQNHNAIMTQNIRQYPVIYNNNR
EVISTGERNYIITVGGPPVTSSQPEYEHISTPNFYQEQRLAQPHPVNESMMIGGYTNPQPISISRGKMLSGNISTNSVRGSNNGYSAKEKKHKAHGKRSN
LPKATVSILNKWLHEHVNNPYPTVQEKRELLAKTGLTKLQISNWFINARRRKIFSGQNDANNFRRKFSSSTNLAKF
>HOMEO_Sc0030 (YHR084W) Ste12
MKVQITNSRTEEILKVQANNENDEVSKATPGEVEESLRLIGDLKFFLATAPVNWQENQIIRRYYLNSGQGFVSCVFWNNLYYITGTDIVKCCLYRMQKFG
REVVQKKKFEEGIFSDLRNLKCGIDATLEQPKSEFLSFLFRNMCLKTQKKQKVFFWFSVAHDKLFADALERDLKRESLNQPSTTKPVNEPALSFSYDSSS
DKPLYDQLLQHLDSRRPSSTTKSDNSPPKLESENFKDNELVTVTNQPLLGVGLMDDDAPESPSQINDFIPQKLIIEPNTLELNGLTEETPHDLPKNTAKG
RDEEDFPLDYFPVSVEYPTEENAFDPFPPQAFTPAAPSMPISYDNVNERDSMPVNSLLNRYPYQLSVAPTFPVPPSSSRQHFMTNRDFYSSNNNKEKLVS
PSDPTSYMKYDEPVMDFDESRPNENCTNAKSHNSGQQTKQHQLYSNNFQQSYPNGMVPGYYPKMPYNPMGGDPLLDQAFYGADDFFFPPEGCDNNMLYPQ
TATSWNVLPPQAMQPAPTYVGRPYTPNYRSTPGSAMFPYMQSSNSMQWNTAVSPYSSRAPSTTAKNYPPSTFYSQNINQYPRRRTVGMKSSQGNVPTGNK
QSVGKSAKISKPLHIKTSAYQKQYKINLETKARPSAGDEDSAHPDKNKEISMPTPDSNTLVVQSEEGGAHSLEVDTNRRSDKNLPDAT
>**HOMEO_Sc0031 (YML027W) Mcm1**
MSQETKMLPSLSSLLSGTEISSSPVSPSFTNPRTSFHLDDRGTIKLPPLNTSINRPRSVESALRHTVTSLHENSSAYGDDMLKHTQSDSALSSQLNSSQE
TVDESHENLLLTPLNSKKRDYSVSSKKNDILTPLSAAKSIIIPSASKEKRRAFAFITHSQETFPKKEPKIDNAPLARRKRRRTSSQELSILQAEFEKCPA
PSKEKRIELAESCHMTEKAVQIWFQNKRQAVKRQRIATSKSTTIIQTVSPPSPPLDVHATPLASRVKADILRDGSSCSRSSSSSPLENTPPRPHHSLNRR
SSTPSIKRSQALTFHLNPQKKTLTPVKTSPNSRVNKLINSIDHSPSKAKRPVSNPSGSPKRKRKFGFKIVDQQPLKDLDPNAFRG
>**HOMEO_Sc0032 (YNL081C)**
MVVHILGKGFKGKEVIKIALASKFYGIGKTTAEKICSKLGFYPWMRMHQLSEPQIMSIASELSTMTIEGDARAIVKDNIALKRKIGSYSGMRHTLHLPVR
GQHTRNNAKTARKLNKIDRRGIHTFSQAKVQHNPSLWSCIFGK
>**HOMEO_Sc0033 (YPL177C) Cup9**
MNYNCEIQNRNSKNVDNQVSLPPIQVLFNSIEKRSMPELAFSNIEYSHGNLRSSTEEQNYPAPVLLPQHHSIAYPAINSGGTSTTATPTASTVETSKTSS
SAMDTQSQYGSSKKSKSASDDAKPCYKSAPIYEIINKEKDAGAQYNRPFSDFVESKSRRKQNSGRRSNLPKETVQILNTWLLNHLNNPYPTQQEKRELLI

KTGLTKIQLSNWFINVRRRKIFSDYYTLVNSIPNDNANNTPVERVQNVSAYHNTLSATNNTMYDATSTCSTDYELSKRFAHAPVTRRKKLIDRLEELKKL
SNPDMN
>**HOMEO_Sp0003 (SPAC32A11.03c)**
MRSYSNPENGGQINDNINYSEKRPTMLPENLSLSNYDMDSFLGQFPSDNNMQLPHSTYEQHLQGEQQNPTNPNYFPPEFDENKVDWKQEKPKPDAPSFAD
NNSFDNVNSSKLTNPSPVQPNIVKSESEPANSKQNEVVEATSVEKAKENVAHESGTPESGGSTSAPKSKKQRLTADQLAYLLREFSKDTNPPPAIREKIG
RELNIPERSVTIWFQNRRAKSKLISRRQEEERQRILREQRELDSLNQKVSQAFAHEVLSTSPTSPYVGGIAANRQYANTLLPKPTRKTGNFYMKSGPMQS
SMEPCIAESDIPIRQSLSSTYYNSLSPNAVPVSSQRKYSASSYSAIPNAMSVSNQAFDVESPPSSYATPLTGIRMPQPESDLYSYPREVSPSSGGYRMFG
HSKPSSYKASGPVRPPNMATGHMRTSSEPTSYDSEFYYFSCTLLVIGLWKRLRASPQDLMCFYSPPKKLFAYLIQFQGIQYRIEYSFFVIESIHVFRVEE
PLLNELSATASSRDKPAPNEYWLQMDIQLSVPPVFHMITSEGQGNCTDFTEGNQASEVLLHSLMGRATSMFQMLDRVRRASPELGSVIRLQKGLNPHQFL
DPQWANQLPRQPDSSVFDHQGRNPPIQGLSHDTSSEYGNKSQFKRLRSTSTPARQDLAQHLLPPKTNTEGLMHAQSVSPITQAMKSANVLEGSSTRLNSY
EPSVSSAYPHHNLALNLDNTQFGELGTSNISYPLSAPSDVGSLPRASNSPSRPVMHPNTQGINTEIKDMAAQFPNSQTGGLTPNSWSMNTNVSVPFTTQN
REFGGIGSSSISTTMNAPSQQLSQVPFGDVSLATENSVPSYGFEVPSEESVYAQARTNSSVSAGVAPRLFIQTPSIPLASSAGQDSNLIEKSSSGGVYAS
QPGASGYLSHDQSGSPFEDVYSPSAGIDFQKLRGQQFSPDMQ
>**HOMEO_Sp0004 (SPBC21B10.13c)**
MSLSDSPSKSGNTGKDLISNNEAKNHEDEETHQKKRRRRTTDAEATLLEQYFLKTPKPSLIERQELSKKLKSSMTPRELQIWFQNKRQSLRRSNCLSRNR
LEGTGENSLLRRKSTLTLCETSTGQAELFFQSWPLHSQSVVGEMIHHEQDDYNKENKQQKVVDTTKDISRGSNGNEDSAAHQELEECARSLVELQQQCND
H
>HOMEO_CnDJ0001 (CND05440)
MEGKNEDMHTPRGPEDASNIADEYPSPERQQQGDMLGHDIHHHLDMRHERDTRDTHSPGQTDMQTELAKQVAAQAIEAAVAHVQANADRQEAEETETCRQ
GSNGNERDIHMREEPHMSNSQHHPRQPVFPPSRQPFTALSPTGVATSIAGPSTLRRSPLPDTFPRPSAPLSANDQIAILRESYARNPNPDRKELERLAAR
TGRPWNKVREYFRQRRNKLRGLEQLEKMEEPGRASGWLQITYRSAPVTSQVPQLTLYNSYRHRFDPYSSSTPLLDALGDNEAQGRKDREVDPEEWERGME
GLVEPLRAGSWLLSSFQSQPGTNGSNITQTDLYTSYAARFSSLLTGVSGTSNGNAHLNGHHGLSDEEAELRNHAESLKAFEDAGLGDTQGEEDQPPTVEE
SDQNEPSPASFPAEPNPLPQAPRESRLLTPVELINLTRMTFPACEPCVDASGRFVIKGLERREGLEPGRKSREGEMFPFALMSEKQPGEEFVKVMKRKLA
SLHPEGTERRSAGESKRKREDVLTEEDKELIEGLKRFRGSKLGEQVRDVCVSQ
>**HOMEO_CnDJ0002 (CND05810) Ste like**
MGSNKHLATGDSHGWPIQSASPSILHRGMTPTTSLPPPTNLAPRPLTERESHLVKHLSRLQFFLATAPTRWMGTGERTNSSFQDNSLSSPHPNLNRFLLP
NGEYVACVFWNGLYHITGTDIIRALVFRFKAFSRPVRNMKKFEEGVFSDLRNLKPGTDACLEEPKSPFLDLLFRNGCIRTQKKARFSVPHDRLFLDALER
DLKREKMGLEPTTIVVGEPARSFRYDPRRSLFEQFAGKQPGLEESVNSSTRDTPAEQLANHDQGATVPKTPLILPPNNLISSSSLPSSIIQNNSIIVSTP
PSPENILNNTEDQLRCLTTASSVLFPRSLLKGSPAYKQGRRKALRDNKQSTRQDSVTISDCDTGDDESGSESERAGRVERNGFERDLALTSQSSAPTLNL
SEDSHATMAITASESTRYISFPSLQSTSGTTLSSHELPSQMLSRQPWATNIIPTTAAATVEQLSANYDQLSAANAFNMAPRLSLQPLGPLIPGRPVLQNS
TVKGFSCPLLSCGRLFKRLEHLKRHVRTHTQERPYECSRCAKRFSRSDNLTQHYKTHERQDRGGRDRSERMKTEASEGADDDITTYLEAQVDAMAGSAHV
YATATDSFVIGEESKSSAVSTEVSPPNRGNQTSISLSSASLLGSHVTNDISAHFLPAGAPHGLPVDVEWPRLGVPLNAISVGEATHDTLNLIKRHRSMTP
SLPPSGRIIGSTRALHSSPYRNNPLYKPYNPYNPYSTNAAIPNGHSFTRAASLDPSVFQDRAAVLSHFVSSKQHTPSQTIPAMCSDRNLYTTPEDLPAHP
VGVDARENAIFITGGGESRLAASSGHQTDYQLSNGLGEASWEMRMRNDSVP
>HOMEO_CnDJ0003 (CND05950) SXI1
MLSTCDSSSQSIRRSLQDDLLDFQETFAKALGKREVHDLESVVQKGNKLVTAIKWSSSRDLLDKNTASLAYAVASSVKLIGSSALELEGGREDAIGEAML
RIESLLLDEGQSRKRQQRNHVPRLKRYCRGSSSESSIAWPKSTFDNSPKPSEPPIDHTVVRLWFLDNLAYPYPTAQQKDFLAKTAGIQRSQVDSDLTNYR
RRAGWTDIMNVWCGGDRNAMKKLMNRVEKGKEQRKKILDAVQGCRDYLTMKENNKVGDWIKEITQNSAFKYRFTGSLPDISAASPCSSIRPCTPRSFSGS
SATSSTSSCSEVSEASAIITIPRKRRYTGDESISPFKRVQGQTVEVSGSYEPWSFADLQPMPPSPGAAQEDIDNEFVAPTTYFISATSLSLSSPAEPLTK
PRLAC
>HOMEO_CnDJ0004 (CND05950)
MLSTCDSSSQSIRRSLQDDLLDFQETFAKALGKREVHDLESVVQKGNKLVTAIKWSSSRDLLDKNTASLAYAVASSVKLIGSSALELEGGREDAIGEAML
RIESLLLDEGQSRKRQQRNHVPRLKRYCRGSSSESSIAWPKSTFDNSPKPSEPPIDHTVVRLWFLDNLAYPYPTAQQKDFLAKTAGIQRSQVDSDLTNYR
RRAGWTDIMNVWCGGDRNAMKKLMNRVEKGKEQRKKILDAVQGCRDYLTMKENNKVGDWIKEVSVLILIFRSSHYFGFPCRLPRPSANKPHRSPRTPPSN
TVSLDLSPTYQPPLHALAFDPALPVPSPVVVPPLAHLLAPKSPRLLPSSLFLERGVTPATSPFLPSNVFKVRLLRSLVHTNLGPSPTCSLCLLLPVPPKR
TSITSLSPPPLTLSVQLLSHYLHLPNLLPSRA
>HOMEO_CnDJ0005 (CNG03450)
MSARQQVPFRELSQEAKMSTSPPLRGIPIDRLSTRSFGREESMSKSSDNHKSVLLSPIARSSSSSPVPRPSHKLSEVVEAVEDKGKKTSQSLPAASLPLI
FRSSPTLPPLNTRNPSRDSSPSSRTGPPPHPTVFHVEHGAFPRKSKEHGHDCPPARPYFPPWYSHPLGYPHPHSSSERHYSSLSRIHTHPEEYYSIHMPT
DRYYNAPLHPYYDLEDYYPPPSPFPAHVYARGKEVYHRIPQPSRPRLHIHPYAFPWSNEGDVRFYSGEGKSNGSQPSHGLGQGPVEGSSDRKLSKGTLSS
PASATSTSAATVMSFKSPRKRTNDVQLAMLSDVFQRTQYPSTEERDELARQLGMTSRSVQIWFQNRRRAVKVDQQSAIQRAEAEARAAEATLRGLPAPPF
LTGSVPGGSGSRRNSDSELEDNTEVDAVMKRERKSPDAGMEG
>**HOMEO_CnDJ0006 (CNI01380)**
MRLSHSHPMTVPPAPLHLLQGHPKEMLELLHQSRGSWDPHFIYRANRAATGAWGAWNSRDEKELSKEYGFEPVKAFWFTGEKGAPDEKPKPRQDGGPVIL
HFHGGGYLCGTAAETDLTSSICKALVSYSPVHHILSVDYRLAPVGPWPLPLLDAISAYHYLVKIEGIPEQDIVIGGDSAGGHLAMALTRWLRDEGDHVGL
SMPRGIVLMSPWGDLGFTNAWGADEYKYNADSDTIDDTFGPFACSLLLRALPLSILYSSPYLSPASSTLSATSLFNNFPPTYIVYGGAERLAKSTETLYS
RIQLARRAADKVLVPDRLFASPDAVHDFVIFPWMAREASQVYEDLDKWLRELLTTDICSPAAASKESLKQPKKITDQCRLTRQRTLESLRSHKSPRMCAA
PDSGMLELVQDMQEEGMSMIEIPKLDLGSTAIT
>HOMEO_CnDJ0007 (CNJ00280)
MSVPAPQQGHPNSLDGYRGYDQQRQPVTGPSNYGRETYIPVSNGYAAYPPNQAGDGSQPARIPIQPHIPPEPHTYQRSYQSQPAAYMHPYAADMHQGMPS
YSYGADVAPTYQFGSLPAAPQSLLPQQSTRPMTAHAGDTVPFGEDGPKDGNDTLNTLEGLQVKHRRRTTPEQLKVLEFWYDINPKPDNQLREQLAAQLGM
TKRNVQVWFQNRRAKMKGLAKKEAEGQESKKSPENQEGTSSAIGPSPPLTDSAVSSSHFNLLPPPASVNMGRRASLANGEAAKIEIFVAKRAAAQKREEV
LYNVGDAGANSPALGQAHAARRGSVPYPTPLASVPPASTSPLSPKFSPAIRGPSTLHMAAVRNNTRRSSIPGVSQLITSGPFTPPRVVSNQHQTAAQSKG
TRELSPIQDHEVYDTYDAEFQPWSSDFPPNMHLPPAEGFHDGRPFSHDSPLPNPAFSFGSAPQGLDMHGIASMGQLDERQQQMFMMMQQRGRLGSIASIG
TMGTETGTEGGESSKGEWLVDGPEGFDPDTRRASAPADLLHQIGLMGFAALPNGAPAPIRPSPLNAHFVPDSFQSTPPYYPPSTSSSSTYSFPLHPSSDS
LSIESPTTAHFEAPKPGSRGEQAHQPQTQSQLQQNHRNLEPGNRMPSYTSTTASHWPNSDVSPYSNHSISYTGYQDGWNPSPQQQAFQQQHLTPSGEHTP
NMGVGVGGGADAGADAGMSIGLGHPSNPHLGHHRDPSPAQQLQVAASVDEKTPTADPPLPQPLGHPGSNSNSLDSRNGQEGGVTGGNEEGKDQFLYFSES
GGHDAVNVLV
>**HOMEO_CnDJ0008 (CNK00090)**
MVDLSALSIPQFSSLSPSDGFSPLKHAFGPSRPLSIAPSDSSSEPLTVSPAGTKTSWALISYSSCCPDNSQDIIDRSPFPTSTNFTSSFSMSQSQQNNDT
YSYRMTPNTGTSQWSQSPTADNSFISSPLTKSIAFQNGLTGISPMFATFNTTSIPNASYPTAAHPINPSCPMPVRGYSASSAVHSPNRRRSSTLVTLSSP
SPMTPPFNHYPYPPLHISYPSTPSSVSARNLFRQPSIPAFSENKRIFHPSPAGKTPATGAKLTQLPYEHGYEGGSQFGSGNMLGGMGMNMETGNPLSLNM
FPQMNTGFRPGNGQEFKLPRFKPTKEQLEILIKSYEENKTPDGPTREALAKKLGPDVRPKTLQIWFQNRRSKSRAKERDAANIPKPLQTNSPTIKPSAQG
HEKGKSGPTRSTSGGPGEMKQGGVNIERLNSLIHDDDPSLSILPITVLSIAKWTRFLTPGTGNICPDLAASIRFRSASTPSHPSPLLPTLHLYVLHTTIF
RIDIPLSASVISNLQATNNPSVITDAVAVRFELGRGQVRFACWIDEEGAGWKEVGDFTGGEAGAGGRVELTGPASVSITSAAFEINKSLLRPTTTDITTS
LFYSPTTPHEYRLSNSYFQFPHLRAHLYPPKAYQCLASPHQCQHSVSLQSNLNPTFLFLTSQPYRHAAPSTFKCQRRYRHDVPLPSRATSCYTSSSRTSE
TAVLLTTDLSDICIDQRSFEPSPVSFIGNSGHSQTPVTGSFNGNATVLGTDQLWESPGGLSDSTSLTLNINEFELVEKK
>**HOMEO_CnDJ0009 (CNL04500)**
MNHFSHAQHIHVVLFFIIIIAFICFPHNTILPEPTMSVCGNSPTPPPPPPPNAIHMHPPQVTPSAASHTSHPTHHTPDARAQQYSIRRAYSTPSIAFPLP
HQAPPSSALTHASYTSNMSENITRYTPGGTPQSSASRPGNGNSKFPPSFDSPTPHGARYNSAVRMDGIEGAEMLESGDLAGIQPPATFPLPEYPASFMRA
EPVPTEETEELQRFLPNDGSVRKYRYGGASCNPWDYMLGDVPDADYDHPLSSRPARYGPDAKHGCKVRRRFTKRELEALEVLWSIAKSPSKYERQRLGAW

LGVKTKHITVWFQNRRQEEKRYSRDGHHDAPPPSRSNRGTFDPVTGKWRPVPASCISGLQPPPDDKIAVVRAISLGDVTRDMWLNKYPSSSGRGMTASAR
VSPTPMSLAATSRGRTIKNAHTTPLLPRNQSRSLDQVLQARESSFGTGAQKRYRRGSGEFVIKGEGQDRIKEILSLMPSDPPSMGLAESDVEESDEDDGG
IDEDVEKRKRAKQAKASSTLAGLGRATPYDVLASSSRAKLLAKPISEHSSRNPVLSQLNPNLSHFAPPSNLRKHTLESVATNQPTKRHRSRVTGHPNPNF
HAGSRTKDFNRSVSTSALPRSSRVAEGQGSSSSSYLPSQLKTPNLGYTRSHSVSSSSSRVITPEDVKDKREGPQMRGQARGQEKDQEVIGAAEMLLQLFG
GS

>**HOMEO_Mgl0001 (MGL_0883)**

MHPELDFLDECLCTLRHVRGTSTPVLPSPVMDAPDAQLDLQSVHSQSFVAALRLRPVSNDVYDALIDLYERVLEQLKRMYMDHFHDAQMRWGSARQRVLP
QIQRLFEVQCQMAARDMQKAILSLVDDRLKTFAADANAPHETHRPHSVRATAILERAFAHAPNITQAEKYKLAQATGLQPRQVTIWVQHACTDNPLQFQN
RRNRRAHARRAAIVDSGLVSPEPELPPPPRLEAPPPPPSVAAARVGQTDDGKDDMLVEGEESGASSLSDWASATTVPSDSLSFDAPIRPFGSSSPAQLAN
STFSTSPLSTTSSLILHASQPHMSPQMQTQAQMTPVPQAHTQTQAQGHTQLPIQVASHPPPPSIPLQSQASMSMTPFQAPASPATLSTAATATNTTTRAS
SLSSTTTTCMPSASTAPIVSLDQLLDLDNARQCLVFSPLDLLPRLDFDDLRLDVPTIENWLGLPSTPGVSSLHVPPISAGAMRSAKLALSQPLNAPDELM
GRAADEGWSLSEQLTPLWNVPTDAHRLSDLSLRIDGTMLRRDSVMKAERMAPPPSAPLTTQSYPLPLSARWNHDAIAPNVLTWDLSRGISSTLVP

>HOMEO_Mgl0002 (MGL_0884)

MLTQEEVRQKLLEFEYTLLDALTREVDESYYSHLRQLTEIVRREFALQQEQGTLTNATLQLGARVSDRLEQLGHILHEQRTQMQRIHSNASQELVLILRS
PIPRSRASGSLRDRRDPTLNAKHMRDWFLRHLGHPFPSREDKEQILAETNACIRDRTLRLKYTQIVLWFINTRRRSGWTAFLRHYARGDKVKLFNLAQAI
ENEEGGTHETRQWSAGHAITLTPASAVQANKPMLSDPNTSSGLSLQSLLPNMDDIARRAMKREWSNIVDRVRIGAKERIGDWVDEVISAPSTASSSSQSR
RHSTRRTTP

>HOMEO_Mgl0003 (MGL_1147)

MGGEQESQEGAPRLDVERLSASSSQGLPHEETGKVQFHTGANCAPAPSSSSPSNQCRLPPIDTPRPLPPHYKYPSRAPLSRLPLFKSNSLPVFTNNYNTS
FGRDIGSTEADAGSAQSGLLRGNAGRYQMRNLLDSPSSFTREHETRFAPDPHRVFSPVYQDMVSRRMHPYASTERKNWDNPRCLSRTSAFNDSNMDESIE
CLKRRLSYQPSLYTHAWNRSFRPTDQFSADFQFRRPNLRYSRMPGRQYMSWTENSHRGYPGSFKDSIGTPFDEWHQHPFYHNGPTLLQTPQERLSANSSV
SKTLSDETSTHGPKSPSATMPATFSTASRPPDNDAHGHSVSTSSDRSPVHDTEGLKYPSVIKPAPPGFSHDQHTSQFFTPKNQYQMANSSEQPVPPKRGG
KLPKHITDMLKTWLLDHADHPYPTEEEKRAFCDFTGLDICQISNWFVNARRRILVPQSSRAAPAAPAPS
