# Supplementary material for: Identification of Homeobox Transcription Factors in a Dimorphic Fungus Talaromyces marneffei and Protein-Protein Interaction Prediction of RfeB
Source: J Fungi (Basel). 2024 Sep 30;10(10):687. doi: 10.3390/jof10100687 (PMC11508405; doi:10.3390/jof10100687)
Supplement: Supplementary file 1 [file jof-10-00687-s001.zip › Supplemental Data S2 and Figures S1-S5.pdf]

Supplemental Data S2.

1. Amino acid sequence

The modeled sequence was highlighted in yellow

>tr|B6QI79|B6QI79_TALMQ Homeobox transcription factor (RfeB), putative OS=Talaromyces marneffei (strain ATCC 18224 / CBS 334.59 / QM 7333) OX=441960 GN=PMAA_096690 PE=4 SV=1

MSSIDSQPAVAGSNVATPTNSTPSPSMTSPAASAPSSSASRRPPRKSTLTQQQKNQKRQR
ATQDQLVTLEQEFNKNPTPTAAVRERIAQEINMTERSVQIWFQNRRAKIKMIAKKSIETG
EDCDSIPDSMRQYLAMHFDPHKPGARELFGRPNGLGGLSNGYGMETTPGKIVIQHFTCRT
LSIGSWRRIGQNAMDLVIFYSPDKATMTYYINNDSAGYKIEYPFSYIKSVMLDNGDLTPN
ANGMPTRPGGLVIELNRPPIFWMDCSNSGGFFQCGDFTEDQQASRVMTHHLGGHPKVLSV
QLAKLVSLETFQNRLDFNYSVSAPITPPQDIHRPASQPNRFTLAQVGIYPDSHLSVNQMP
RGHKRQRSRSVPAAIDLSYLHTPIAPFPLQHPQVSHMAPNPNMYAPIPQNPNALHALGND
LRINTAATFVDPQSYPMSATTMSDFAVASPSFFSAAPPTESIAITGNPGDQFNMPYVSPS
PMLDQSKMMNQPPTSMANFSHADPLIANHSPPLSTLHNTISNDMFNFGADQQQGMTEDGF
VLSEMYAKHHINQFPDGPSFDFSMNALSETQSPMPSDMHGFETIQTTSA

>tr|B6Q5R4|B6Q5R4_TALMQ C2H2 transcription factor Swi5 OS=Talaromyces marneffei (strain ATCC 18224 / CBS 334.59 / QM 7333) OX=441960 GN=PMAA_023500 PE=4 SV=1

MLVSPSSGMQRHQRQHRRQNSIPVALEATKPPHLPAAAMQRYSMHKRGMSLNQPSANLQP
NPLSTSQSQYTNVLREAQQQRTSQLNQQSYFDEPQNQILSNADSQRLETRGLNVYTNQYS
NENSPINACMSSGGLNIKNTNIRNRQLKPAIQPMQQQQQQPGQETMIFAENQLIGDGTWN
AYLVNQSMLAQMYDLRRASVQSDISQQPYIPSTPPKQVQSNYVPITPDTTPFRRGTEFAP
FMQNVHASPIKNIVYQTQPAYMQRAKSLQGVPGSNYAEPKIDVPSPPNTAPVDYDCYDLL
TSQESDFESTEFQHVPKSMSIKSEDHESYNTQILSTTNSFQSSPEIAYMPLPNSSLIKAP
KVPISVATPSKPSSTKSNSPTPDFSPSKTRLSPKVASIDSLNLDARVQASITETGITIDE
IAAYISGPDPVDGKWVCIHPGCDRRFGRKENIKSHIQTHLGDRQYKCDHCEKCFVRGHDL
KRHAKIHTGDKPYECLCGNVFARHDALTRHRQRGMCIGGYKGVVRKTTKRGRPKKSRPDM
EERQDKAARTREKVSGNSATISMSSSDSSCSSPPSDGFESMSIQASSPLEDMAMFESANY
CLLSEVFSFTPPASPGFSTGHHSSPGQSSRSYSPLSDVGSMSRTSSRHPLEDISEEIPDL
PPISEAAGCFDPEPENSLQTDLPSSAIVPALTHSTAGSDIDIFINNNSSFSDFGPESGIP
NDMDLFSGKSVGVNDSDFFLDFNDDHTTDAFF

> NP_010177.1 Pho2p [Saccharomyces cerevisiae S288C]

MMEEFSYDHDFNTHFATDLDYLQHDQQQQQQQQHDQQHNQQQQPQPQPIQTQNLEHDHDQ
HTNDMSASSNASDSGPQRPKRTRAKGEALDVLKRKFEINPTPSLVERKKISDLIGMPEKN
VRIWFQNRRAKLRKKQHGSNKDTIPSSQSRDIANDYDRGSTDNNLVTTTSTSSIFHDEDL
TFFDRIPLNSNNNYYFFDICSITVGSWNRMKSGALQRRNFQSIKELRNLSPIKINNIMSN
ATDLMVLISKKNSEINYFFSAMANNTKILFRIFFPLSSVTNCSLTLETDDDIINSNNTSD
KNNSNTNNDDDNDDNSNEDNDNSSEDKRNAKDNFGELKLTVTRSPTFAVYFLNNAPDEDP
NLNNQWSICDDFSEGRQVNDAFVGGSNIPHTLKGLQKSLRFMNSLILDYKSSNEILPTIN
TAIPTAAVPQQNIAPPFLNTNSSATDSNPNTNLEDSLFFDHDLLSSSITNTNNGQGSNNG
RQASKDDTLNLLDTTVNSNNNHNANNEENHLAQEHLSNDADIVANPNDHLLSLPTDSELP
NTPDFLKNTNELTDEHRWI

>NP_010430.1 DNA-binding transcription factor SWI5 [Saccharomyces cerevisiae S288C]

MDTSNSWFDASKVQSLNFDLQTNSYYSNARGSDPSSYAIEGEYKTLATDDLGNILNLNYG
ETNEVIMNEINDLNLPLGPLSDEKSVKVSTFSELIGNDWQSMNFDLENNSREVTLNATSL
LNENRLNQDSGMTVYQKTMSDKPHDEKKISMADNLLSTINKSEINKGFDRNLGELLLQQQ
QELREQLRAQQEANKKLELELKQTQYKQQQLQATLENSDGPQFLSPKRKISPASENVEDV
YANSLSPMISPPMSNTSFTGSPSRRNNRQKYCLQRKNSSGTVGPLCFQELNEGFNDSLIS
PKKIRSNPNENLSSKTKFITPFTPKSRVSSATSNSANITPNNLRLDFKINVEDQESEYSE
KPLGLGIELLGKPGPSPTKSVSLKSASVDIMPTIPGSVNNTPSVNKVSLSSSYIDQYTPR
GKQLHFSSISENALGINAATPHLKPPSQQARHREGVFNDLDPNVLTKNTDNEGDDNEENE
PESRFVISETPSPVLKSQSKYEGRSPQFGTHIKEINTYTTNSPSKITRKLTTLPRGSIDK
YVKEMPDKTFECLFPGCTKTFKRRYNIRSHIQTHLEDRPYSCDHPGCDKAFVRNHDLIRH
KKSHQEKAYACPCGKKFNREDALVVHRSRMICSGGKKYENVVIKRSPRKRGRPRKDGTSS
VSSSPIKENINKDHNGQLMFKLEDQLRRERSYDGNGTGIMVSPMKTNQR

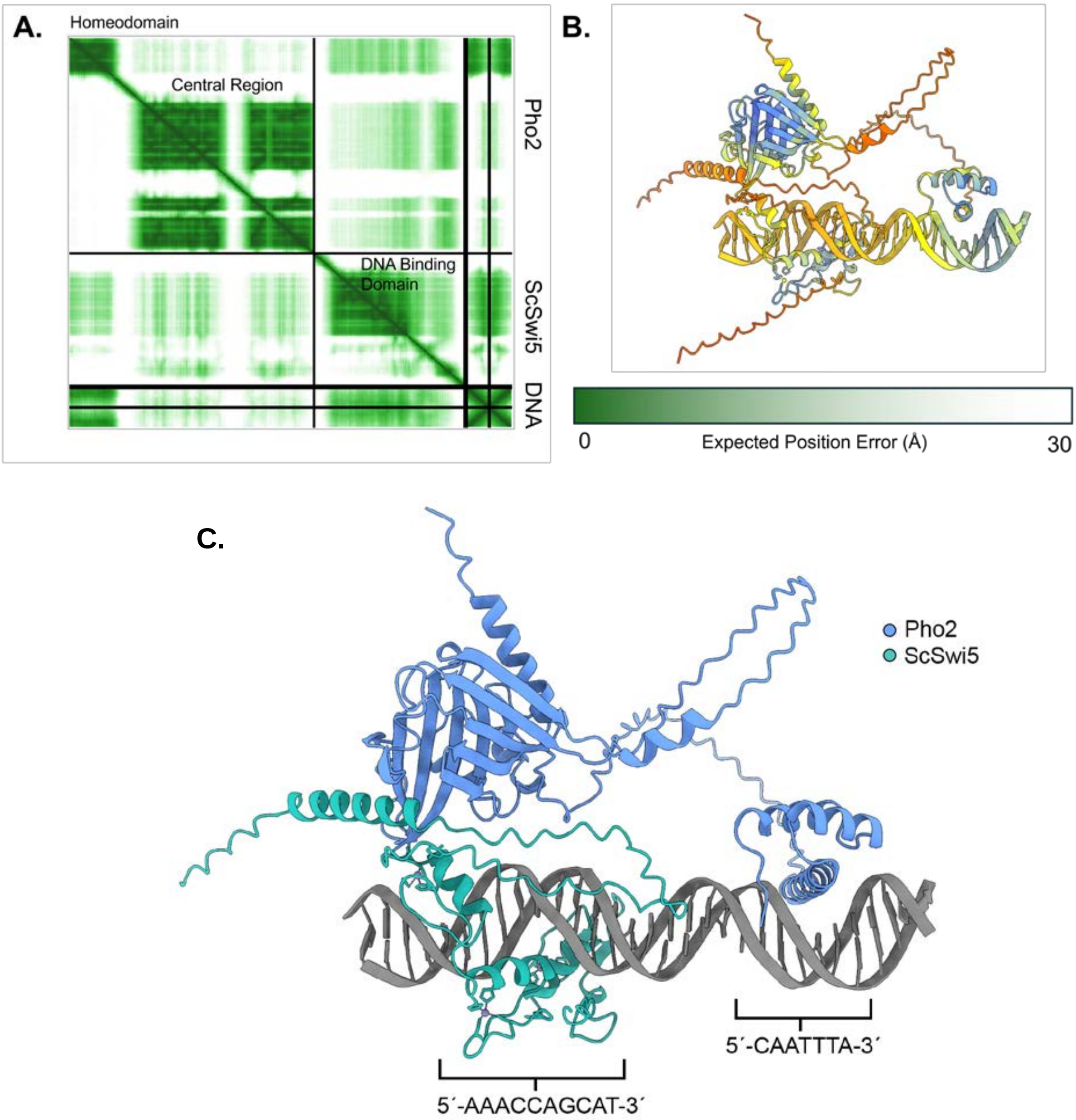

Figure S1. Summary of predicted quality. **A**. plDDT score. **B**. predicted Pho2-ScSwi5 complex. **C**. The cleaned structure of Pho2-ScSwi5 complex.

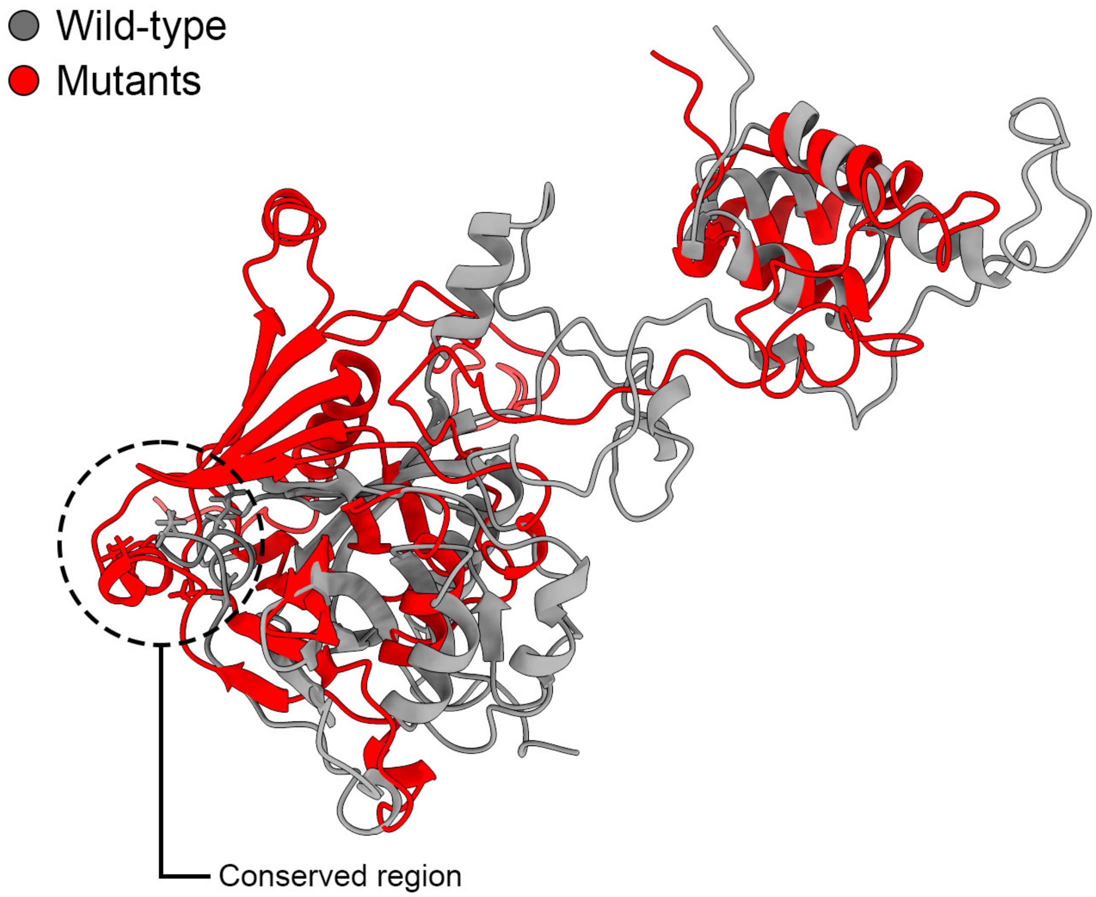

Figure S2. Superimposition between wild-type and mutant Pho2 from 500 ns molecular dynamics simulations.

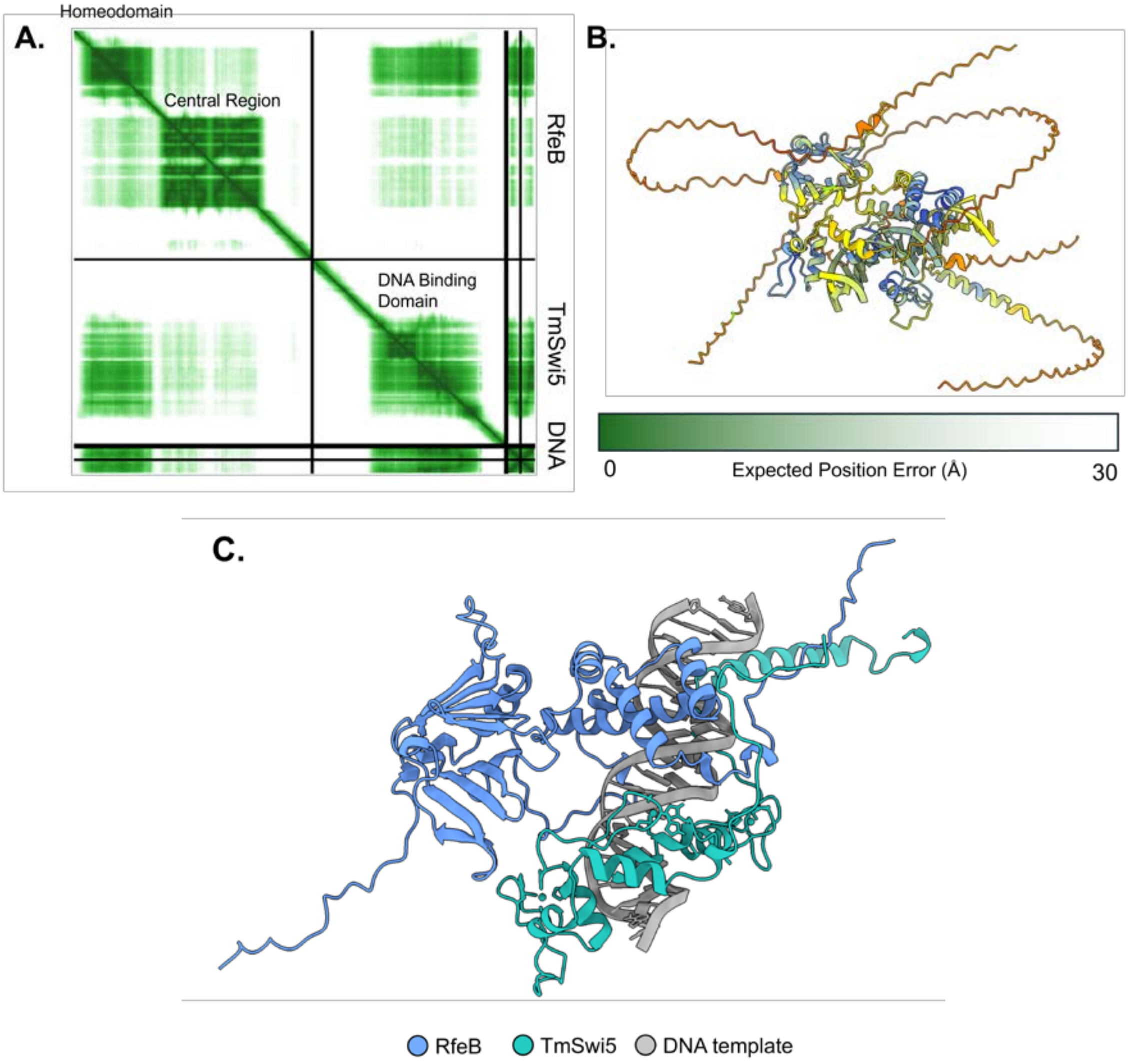

Figure S3. Summary of predicted quality. **A**. plDDT score. **B**. predicted RfeB-TmSwi5 complex. **C**. The cleaned structure of RfeB-TmSwi5 complex.

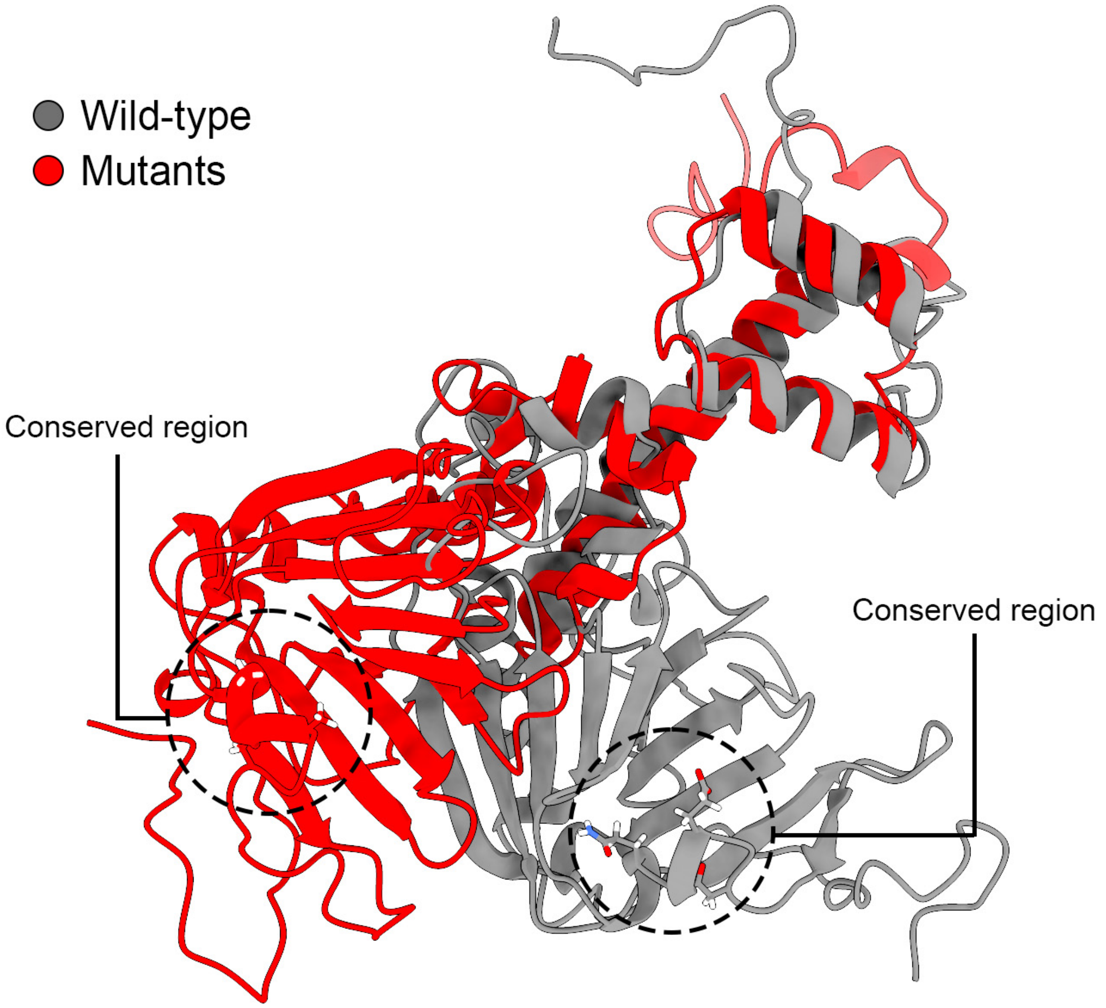

Figure S4. Superimposition between wild-type and mutant RfeB from 500 ns molecular dynamics simulations.

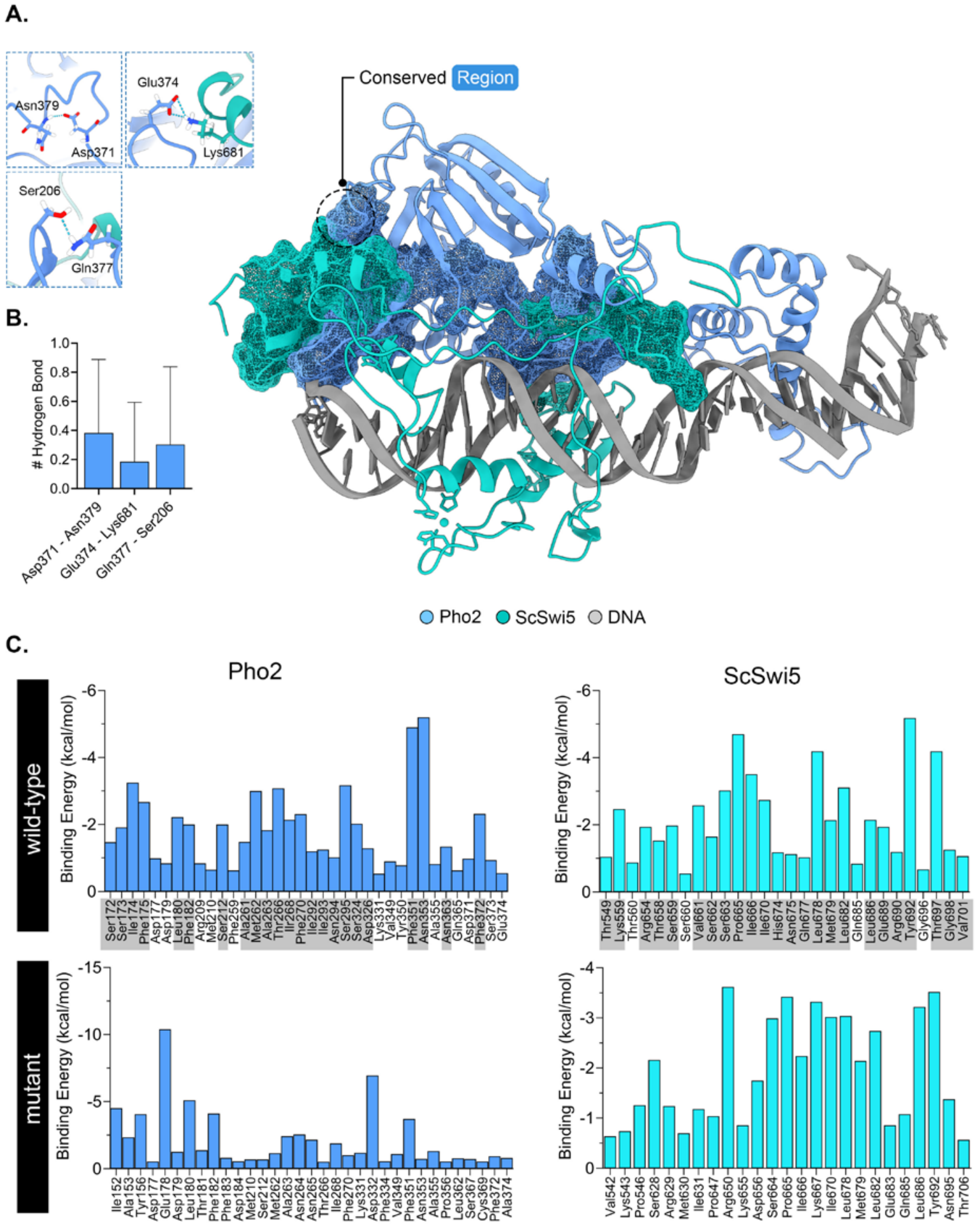

Figure S5. Determination of key residues that contribute to protein binding and stability. Residues essential for intra- (A. and B.) and inter- (C.) protein interactions were determined. Binding sites of Pho2 that directly contact with ScSwi5 were identified and illustrated. B. Hydrogen bonds formed by the conserved IR residues (Asp371, Glu374, and Gln377) were determined. C. Per-residue energy decomposition was calculated and

compared between the ternary complexes formed by the wild type (top) vs mutated Pho2 proteins (bottom). Highlighted in grey was the key Pho2 and ScSwi5 residues that interact with each other (binding energy less than -1 kcal/mol).
